# Multi-bioinformatics revealed potential biomarkers and repurposed drugs for gastric adenocarcinoma-related gastric intestinal metaplasia

**DOI:** 10.1038/s41540-024-00455-0

**Published:** 2024-11-04

**Authors:** Gøran Troseth Andersen, Aleksandr Ianevski, Mathilde Resell, Naris Pojskic, Hanne-Line Rabben, Synne Geithus, Yosuke Kodama, Tomita Hiroyuki, Denis Kainov, Jon Erik Grønbech, Yoku Hayakawa, Timothy C. Wang, Chun-Mei Zhao, Duan Chen

**Affiliations:** 1 Department of Clinical and Molecular Medicine, Norwegian University of Science and Technology (NTNU), Trondheim, Norway; 2 Department of Surgery, St. Olav’s Hospital, Trondheim, Norway; 3 Department of Surgery, Namsos Hospital, Namsos, Norway; 4 Laboratory for Bioinformatics and Biostatistics, University of Sarajevo - Institute for Genetic Engineering and Biotechnology, Sarajevo, Bosnia and Herzegovina; 5 Department of Tumor Pathology, Gifu University Graduate School of Medicine, Gifu, Japan; 6 Department of Gastroenterology, Tokyo University Hospital, Tokyo, Japan; 7 Department of Digestive and Liver Diseases and Herbert Iring Comprehensive Cancer Center, Columbia University Medical Center, New York, USA

**Keywords:** Gastroenterology, Systems biology

## Abstract

Biomarkers associated with the progression from gastric intestinal metaplasia (GIM) to gastric adenocarcinoma (GA), i.e., GA-related GIM, could provide valuable insights into identifying patients with increased risk for GA. The aim of this study was to utilize multi-bioinformatics to reveal potential biomarkers for the GA-related GIM and predict potential drug repurposing for GA prevention in patients. The multi-bioinformatics included gene expression matrix (GEM) by microarray gene expression (MGE), ScType (a fully automated and ultra-fast cell-type identification based solely on a given scRNA-seq data), Ingenuity Pathway Analysis, PageRank centrality, GO and MSigDB enrichments, Cytoscape, Human Protein Atlas and molecular docking analysis in combination with immunohistochemistry. To identify GA-related GIM, paired surgical biopsies were collected from 16 GIM-GA patients who underwent gastrectomy, yielding 64 samples (4 biopsies per stomach x 16 patients) for MGE. Co-analysis was performed by including scRNAseq and immunohistochemistry datasets of endoscopic biopsies of 37 patients. The results of the present study showed potential biomarkers for GA-related GIM, including GEM of individual patients, individual genes (such as RBP2 and CD44), signaling pathways, network of molecules, and network of signaling pathways with key topological nodes. Accordingly, potential treatment targets with repurposed drugs were identified including epidermal growth factor receptor, proto-oncogene tyrosine-protein kinase Src, paxillin, transcription factor Jun, breast cancer type 1 susceptibility protein, cellular tumor antigen p53, mouse double minute 2, and CD44.

## Introduction

The prevalence of gastric intestinal metaplasia (GIM) is approximately 15% in those undergoing routine endoscopy in Europe^1,2^. GIM can be found in approximately 50% of patients with gastric ulcers and almost 100% of patients with intestinal type gastric adenocarcinoma (GA), the major subtype of gastric cancer^3^. According to Correa’s cascade^4^, the progression rate from GIM to GA varies from 0.25% to 42% during a course of 5 years^5,6^, and this malignant conversion can occur even after *H. pylori* eradication^7,8^. Nevertheless, it remains unclear whether GIM glands can directly transform into GA or share a clonal origin^9–11^, despite GIM being susceptible to somatic mutations and copy number aberrations commonly found in GA^12^.

Currently, the American Gastroenterological Association (AGA), the European Society of Gastrointestinal Endoscopy and the British Society of Gastroenterology recommend no further intervention against GIM, except for the eradication of *H. pylori*^13–15^. Of note, in patients with GIM, the AGA suggests against routine use of endoscopic surveillance and routine repeat short-interval endoscopy with biopsies for the purpose of risk stratification. Based on H&E staining, histological classification of GIM has been suggested, as incomplete GIM (goblet cells without a brush border) was associated with a greater risk of progression to GA in comparison to complete GIM (with the brush border), but more investigation on the potential benefit of implementing this routine pathological characterization is needed^13,16–19^. Thus, routine endoscopic surveillance with 3- and 5-year intervals is conditionally recommended in patients diagnosed with incomplete GIM or if there are any additional risk factors, i.e., family history and extensive GIM. On the other hand, in countries with a high risk of gastric cancer such as Japan or South Korea, annual endoscopic surveillance is recommended. Observational studies suggest that advanced GIM and long-term use of acid suppression are associated with higher risk of GA development after *H. pylori* eradication^7,20–22^.

Biomarkers associated with the progression from GIM to GA could provide valuable insights into identifying patients with increased risk for GA. This information could help in the targeted selection of individuals who would benefit the most from screening and surveillance endoscopy. While biomarkers such as pepsinogen (I and II) levels are commonly used in Asian countries, their studies and applications in the United States and Europe remain limited^13^. CD10, a brush border protein specific to normal small intestinal mucosa and absent in the colon^23^, contrasts with Das1, a monoclonal antibody that binds colonic epithelial protein^24^. CD10 and Das1 have been suggested as potential candidate biomarkers to distinguish complete and incomplete GIM based on gene expression data and immunohistochemistry^24^. Somatic mutations in GA can be found in adjacent GIM^25^. Currently, the discovery of effective cancer biomarkers poses a challenge due to considerations of predictive efficacy and clinical application. Rarely can gastric tumorigenesis be attributed solely to an individual molecule, such as a gene or protein, as GA progression is believed to arise from the collective interactions of multiple molecules. Thus, bioinformatics advancements could show promise; for instance, reports on progression gene signatures for lung cancer and glioblastoma have emerged from single-cell RNA sequencing (scRNA-seq) and other transcriptomic analyses^26^. Single-cell atlases based on scRNA-seq are increasingly accessible for a variety of tissues, organs, and organisms, offering new prospects from data mining to gaining biological insights^27^. Such single-cell atlases are available for GA and GIM^28,29^. Nevertheless, various sets of genes specific to GA have been suggested. The differences in GA biomarker proposals in these separate studies could be attributed to several potential reasons, including distinct methods for clinical tissue sampling, diverse annotation approaches for cell clusters, and disparate data mining techniques^28,29^.

In the present study, we aimed to utilize the multi-bioinformatics to reveal potential biomarkers for a subset of patients with progression from GIM to GA and to identify the drug targets and accordingly repurposed drugs. Thus, we compared the microarray gene expression (MGE) of GIM, GA and normal tissues within the individual patients, enabling to identify GA-related GIM. We utilized the newly developed computational platform ScType for cell-type identification based on given scRNA-seq data along with a comprehensive cell marker database as background information^30^. We then created heatmaps of the individual patients and of the pathological diagnosis with sub-classifications by gastric histological activity (inflammation, epithelial defects, mucosal atrophy, hyperplasia, pseudopyloric metaplasia, and dysplasia or neoplasia), locations (antrum, cardia, corpus ventriculi, major and minor curvatures), and cell types (e.g. goblet cells, metaplastic stem-like cells, chief cells, and cancer cells). Furthermore, we used bioinformatics including Ingenuity Pathway Analysis (IPA), pagerank centrality, GO and MSigDB enrichments, Human Protein Atlas, Cytoscape, ScType and molecular docking analysis. We found a network consisting of 865 RNA markers that might be associated with the progression from GIM to GA. Some of these RNAs encode proteins in the network could serve as potential targets for drug repurposing as well as drug development for the prevention of the progression from GIM to GA.

## Results

### GIM and GA displayed convoluted gene expression matrixes (GEM)

**Fig. 1:**
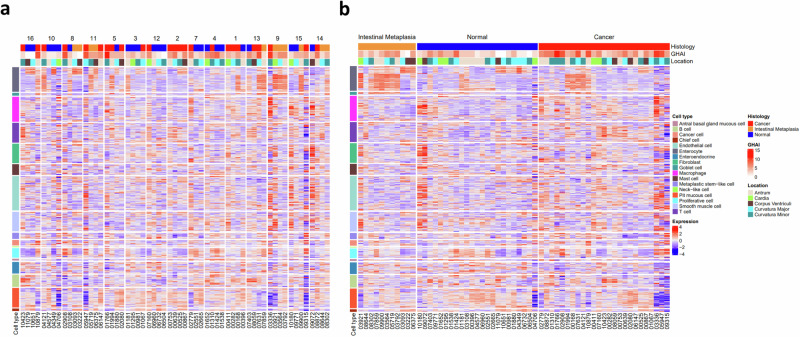
GEM. Of note, the gene expression heatmaps annotated according to histology, GHAI and location of lesions as well as single cell atlas in individual patients (**a**) and in GIM, normal and GA (**b**). Patient numbers (1-16, except 6) and sample numbers below heatmaps were included.

GEM was created according to histology, gastric histologic activity index (GHAI), and location of lesions (antrum, cardia, corpus, major and minor curvature) in combination of single cell atlas (including various cell types, such as goblet cells, chief cells, pit mucosa cells, stem-like cells and immune cells). It showed convoluted profiles in individual patients and in comparisons between GIM, GA and normal tissues (Fig. 1a, b).

### GIM displayed higher levels of gene expression than GA

**Fig. 2:**
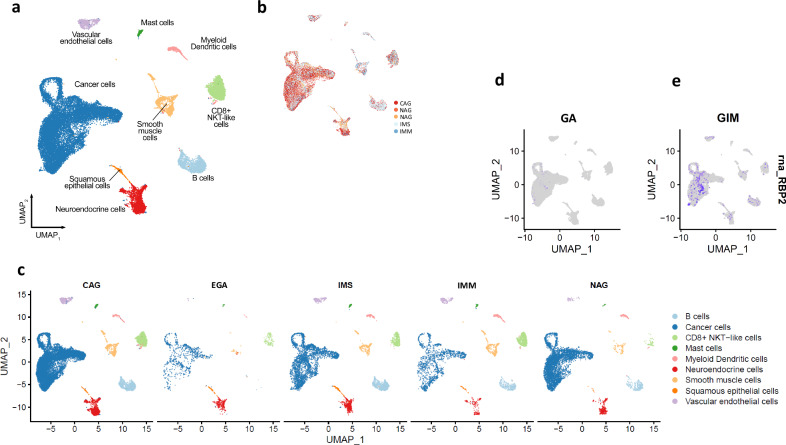
UMAP plots. Of note, cell type clusters (**a**) and annotations according to the pathological appearances (**b**, **c**) and low expression of the RBP2 gene in GA (**d**) but high expression in GIM (**e**). CAG chronic atrophic gastritis, EGA early gastric adenocarcinoma, IMS severe intestinal metaplasia, IMM mild intestinal metaplasia, and NAG non-atrophic gastritis.

Co-analysis using ScType visualized nine cell types, including cancer cells and tumor microenvironment-related cells, such as vascular endothelial cells, smooth muscle cells, CD8 + NKT-like cells, B cells, myeloid dendritic cells, mast cells, squamous epithelial cells and neuroendocrine cells (Fig. 2a). Furthermore, it annotated the cell type-related gene expression according to five pathological appearances, including chronic atrophic gastritis (CAG), early gastric cancer (EGA), severe intestinal metaplasia (IMS), mild intestinal metaplasia (IMM), and nonatrophic gastritis (NAG) (Fig. 2b, c). EGA displayed the lowest gene expression in relation to cancer cells, vascular endothelial cells, smooth muscle cells, myeloid dendritic cells, B cells, CD8 + NKT-like cells, and mast cells among the five pathological appearances. GIM, including both IMM and IMS, displayed similar distributions of cell-type gene expression in comparison with NAG or CAG. Differential expression analysis between GIM and GA showed gene profiles and identified genes that were uniquely expressed in cancer cells, particularly RBP2 expression not in GA but in GIM (Fig. 2d, e).

### GIM exhibited different signaling pathways than GA and displayed GA-related molecular network

**Fig. 3:**
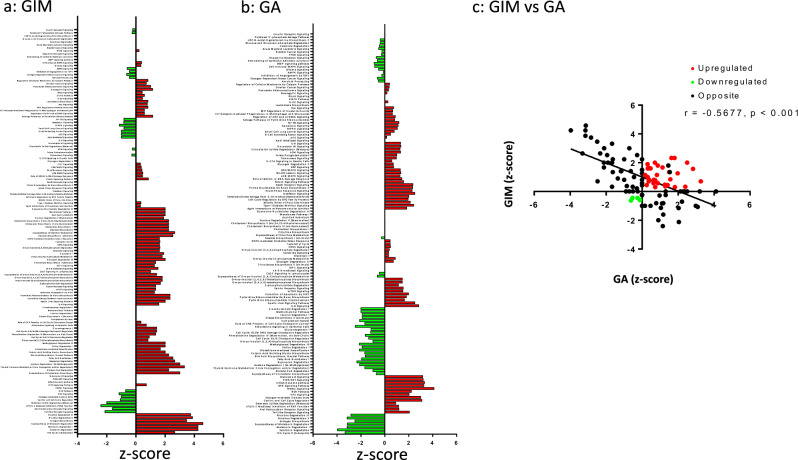
Study design. Canonical pathway activation and inhibition in GIM (**a**) and GA (**b**). Note: Pathways with obtained z-scores included [z-score > 0: activation (red); z-score < 0: inhibition (green); z-scores from −4 to 6 in (**a**) and from −6 to 6 in (**b**)]. Pearsons correlation with linear regression between GIM and GA (c). r = -0.5677 and *p* < 0.001 in (**c**).

Pathway analysis showed 110 canonical pathways that were either activated or inactivated in GIM or GA (Fig. 3a, b and supplementary Table S1). It should be noticed that there was a negative (albeit weak) correlation between GIM and GA (Fig. 3c). In particular, activated pathways in both GIM and GA were regulation of cellular mechanics by calpain protease, neuregulin signaling, IL-22 signaling, Rac signaling, MIF regulation of innate immunity, Fcγ receptor-mediated phagocytosis in macrophages and monocytes, regulation of elF4 and p7056K signaling, salvage pathway of pyrimidine ribonucleotides, HGF signaling, ERK/MAPK signaling, Wnt/β-catenin signaling, p38 mark signaling, role of BRCA1 in DNA damage response, sirtuin signaling pathway, NRF2-mediated oxidative stress response, γ-glutamyl cycle, CDK5 signaling, D-myo-inositol-5-phosphate metabolism, glycogen degradation, ephrin receptor signaling, mTOR signaling, IL-8 signaling, and apelin liver signaling pathway. HIPPO, p53 toll-like receptor signaling, aryl hydrocarbon receptor signaling, LPS/IL-1-mediated inhibition of RXR function, EIF2 signaling, and the NER pathway were inactivated in GIM but activated in GA, whereas fatty acid β-oxidation, nicotine degradation II and III, estrogen biosynthesis, melatonin degradation I, the superpathway of melatonin degradation and serotonin degradation were activated in GIM but inactivated in GA.

Microarray gene expression (MGE) revealed 19,223 out of 20,918 gene IDs and scRNAseq mapped 17,921 out of 22,910 gene IDs. A core analysis was run on each dataset with the criteria of adjusted *p*-value < 0.05 and an absolute log2FoldChange of 1. Co-analysis of MGE and scRNAseq was used to filter the genes in scRNAseq dataset and showed that 1,158 genes were expressed in both datasets (excluding the genes that were present in more than one cell cluster). Furthermore, 865 out of 1158 genes had equivalent IDs in IPA (Table S2).

**Fig. 4:**
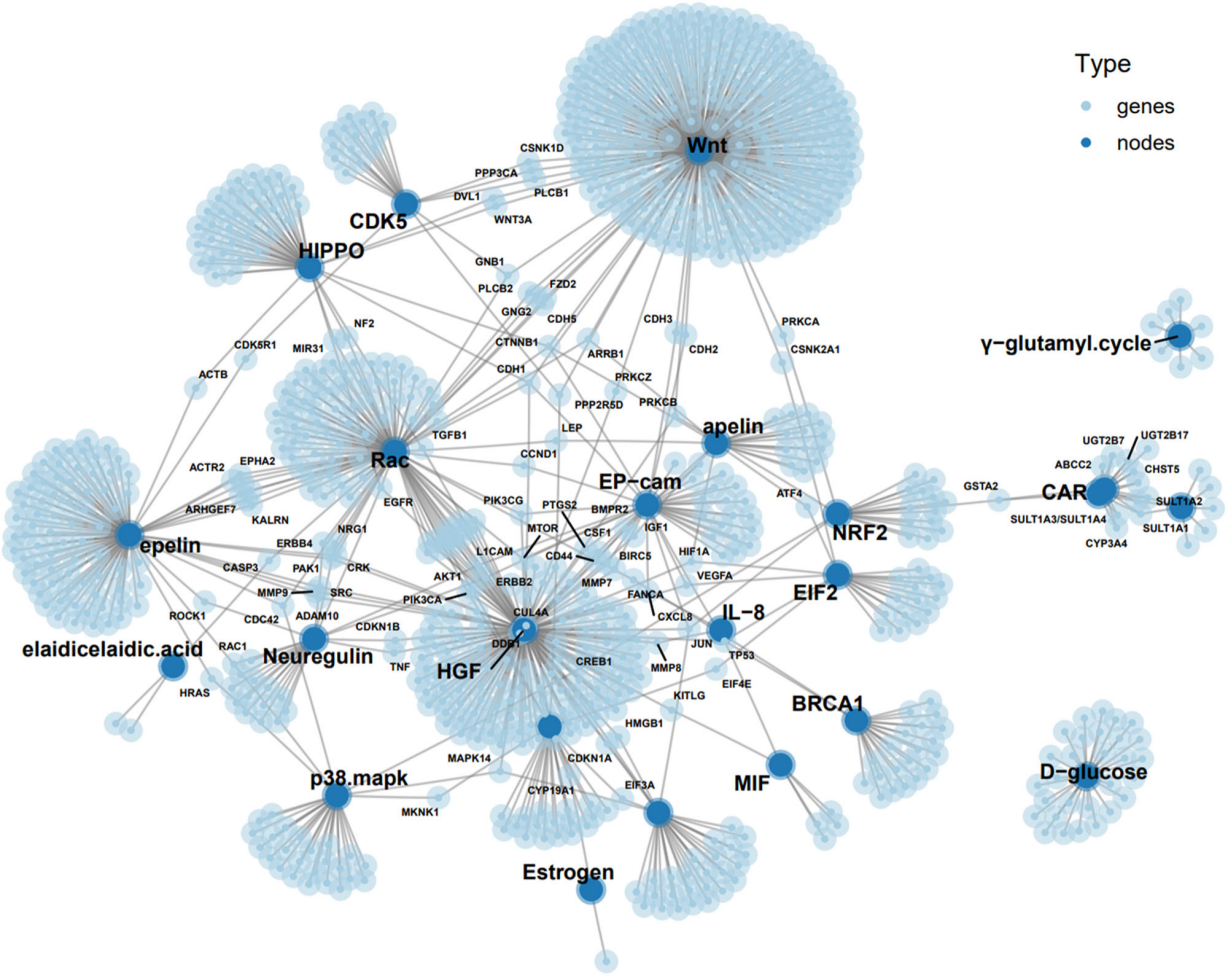
Network of molecules highlighting 23 proteins/genes/metabolites characteristic to GA-related GIM, particularly in connection to stem cell-related Wnt-HIPPO genes. Note: sizes of nodes and genes reflect numbers of interactions.

Pathway enrichment analysis using a combination of three databases, i.e., Protein-Protein Interaction Networks Functional Enrichment Analysis (https://string-db.org/), Protein, Genetic and Chemical Interactions (https://thebiogrid.org/) and IntAct Molecular Interaction Database (https://www.ebi.ac.uk/intact/home) was performed. A biomarker network of the 865 genes in connection with the genes/proteins and metabolites was created (Fig. 4). Of note, this analysis reveals not only protein-protein interactions but also genetic and chemical interactions (e.g. elaidicelaidic acid and D-glocose). The most influential node was Wnt signaling pathway which has directly connections with HIPPO signaling, hepatocyte growth factor (HGF) signaling, CDK5 (pro-apoptotic signaling), Rac signaling, and EpCAM signaling and indirectly connections with other signaling and metabolic pathways (Fig. 4).

**Fig. 5:**
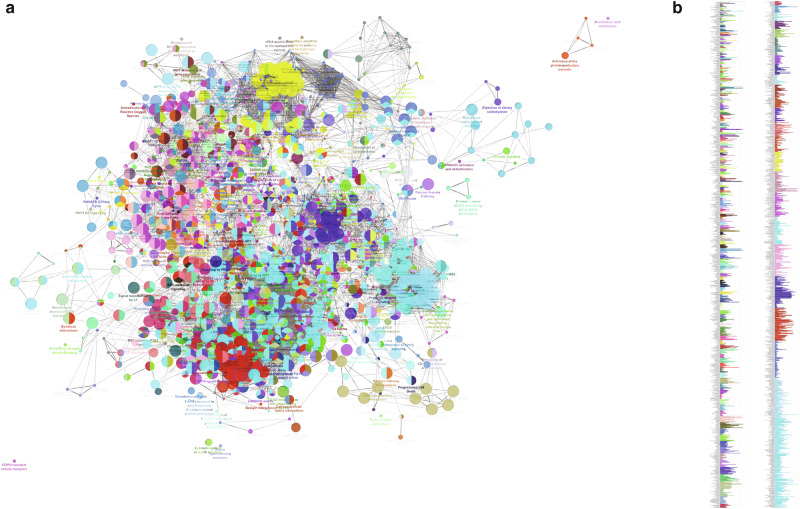
Network of signaling pathway characterized as ‘Team’ for GA-related GIM. Note: sizes of nodes reflect percentages of team-related genes per pathway and links presents the connections between pathways (**a**); percentages of gene/team (**b**) (for detailed percentages of each team, see Table S3).

Furthermore, functional enrichment analysis of the 865 genes using Cytoscape (including NetworkAnalyzer) was performed. It revealed a comprehensive network of signaling pathways or terms (Fig. 5, Table S3) and topological coefficient (Table 1 and Table S4). Of note, there are overlaps, to a large extent, between the significant pathway and the gene ontology term. The Cytoscape analysis confirmed (with *p* < 0.01) the involvements of Wnt and Wnt-related signaling (Wnt signaling in cancer, Wnt target genes, Wnt5A-dependent internalization of FZD4, Wnt mediated activation of DVL, Hippo, Yap1, RUNX3 regules Wnt signaling, catenin Beta 1 (CTNNB1) and biding if TCF/LEF: CTNNB1 to target gene promoters). It was noticed that ‘lactose synthesis’ was nearly 100% involved (Table S3).

**Table 1:** Selected key nodes with topological coefficients

Term	T_n_	*p*-Value*	UNIQUER_ID
Cytochrome c-mediated apoptotic response	1.0	0.0307	HSA:111461
COPI-mediated anterograde transport	1.0	0.0323	HSA:6807878
Membrane Trafficking	0.95	0.0171	HSA:199991
Vesicle-mediated transport	0.95	0.0099	HSA:5653656
Laminin interactions	0.93	0.0298	HSA:3000157
Apoptosis	0.92	0.0322	HSA:109581
Programmed Cell Death	0.92	0.0487	HSA:5357801
Disorders of Nervous System Development	0.87	0.0330	HSA:9697154
Asparagine N-linked glycosylation	0.84	0.0281	HSA:446203
Intrinsic Pathway for Apoptosis	0.75	0.0364	HSA:109606
Apoptotic factor-mediated response	0.75	0.0281	HSA:111471
Signaling by RAS mutants	0.52	0.0459	HSA:6802949
Paradoxical activation of RAF signaling by kinase inactive BRAF	0.52	0.0459	HSA:6802955
Signaling downstream of RAS mutants	0.52	0.0459	HSA:9649948
Signaling by RAF1 mutants	0.52	0.0351	HSA:9656223
Axon guidance	0.50	0.0167	HSA:422475

T_n_: topological coefficient (T_n_ = avg (J(n,m)) / k_n_.).

*p Value: Corrected with Benjamini-Hochberg.

The stability of network in resisting to external interference and attacks depends on key nodes^31^. The network topological analysis revealed potential key nodes. Of note, cytochrome c-mediated apoptosis, COPI-mediated anterograde transport, membrane trafficking, vesicle-mediated transport, laminin interactions and apoptosis were among the top key nodes. Other key nodes included disorders of nervous system development and axon guidance, Ras mutation and RAF signaling (Table 1). (for a completed list of pathways/terms, see Table S4).

### Computational predictions of potential drug targets and drug repurposing

**Fig. 6:**
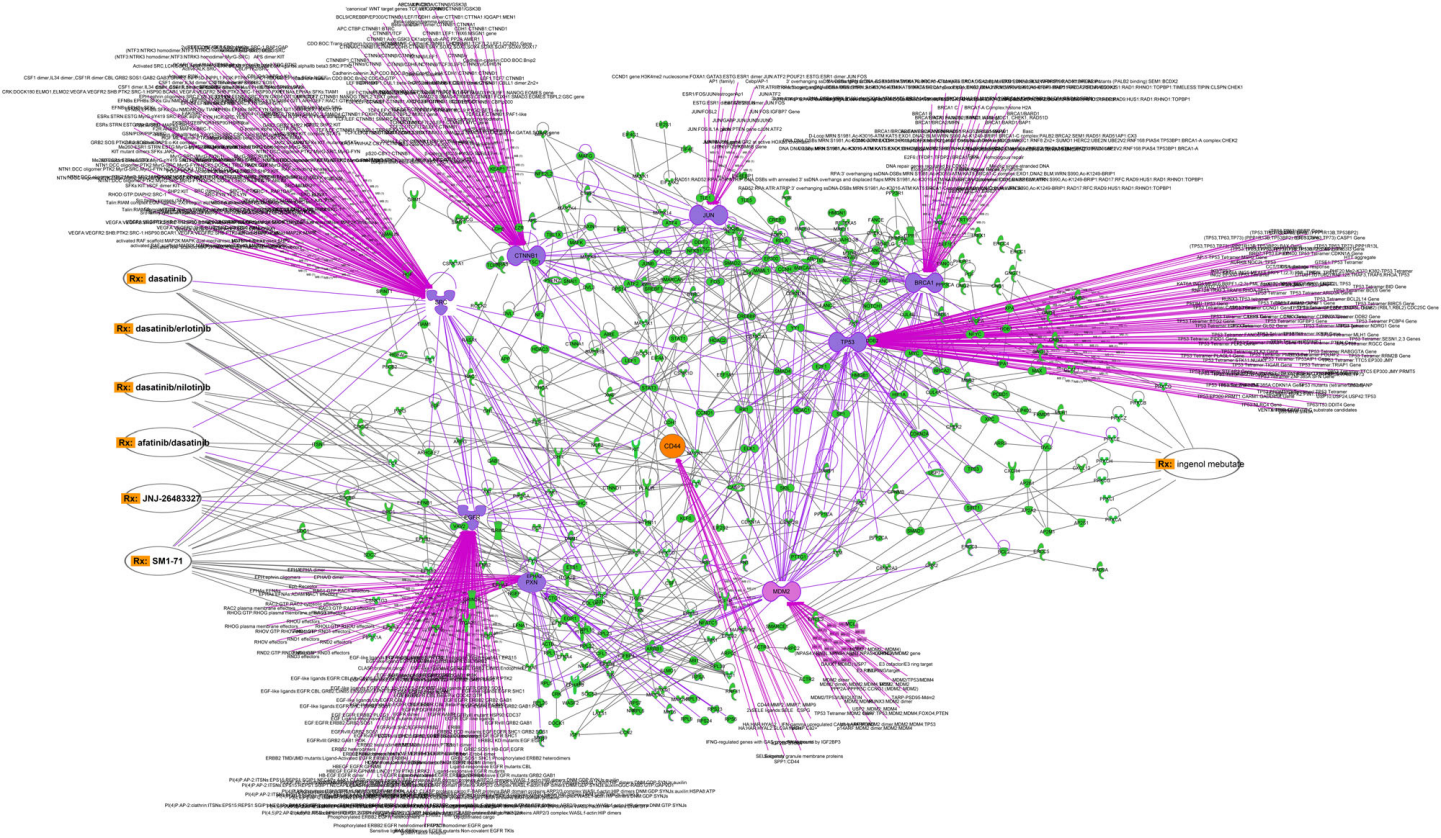
Protein-protein interactions. Note: Hub proteins in GIM-GA “niche” (indicated in dashed line) and links with the repurposed drugs (Rx, indicated in orange) revealed by IPA analysis.

Protein‒protein interactions (PPI) analysis using IPA showed 330 out of 865 GIM-related proteins having PPI > 3 were identified, particularly, including epidermal growth factor receptor (EGFR), proto-oncogene tyrosine-protein kinase (SRC), paxillin (PXN), Jun proto-oncogene (JUN), breast cancer type 1 susceptibility protein (BRCA1), tumor protein P53 (TP53 or p53), mouse double minute 2 homolog (MDM2) (also known as E3 ubiquitin-protein ligase MDM2), and CTNNB1 (catenin β1) and CD44 (Fig. 6, Table 2 and Table S5). It should be pointed out that CD44 was selected because CD44 is an important biomarker of stem cells, particularly in connection with Wnt signaling^32–36^. It has been known that Wnt/β-catenin pathway and mucins play important roles in regulating neoplastic transformation and malignant growth, including GA^33,37,38^. In GIM, CD44 had PPIs with MMP2, MMP7, MMP9, IFN_ϒ_, SLC9A and CSPGs (Fig. 6). IPA drug repurposing of approved and investigational drugs/compounds (e.g., existing at www.clinicaltrial.gov) showed the following targeted drugs: dasatinib with or without erlotnib, nilotinib, afatinib on SRC, SM1-71 on SRC, JNJ-26483327 on EGFR, Afatinib on EGFR, and ingenol mebutate on protein kinase C (PKC) family (Fig. 6).

**Table 2:** Hub proteins with drug-repurposing targets

Protein name	Degree	Drugs
EGFR	92	JNJ-26483327, Dasatinib, dasatinib/erlotinib, afatinib/dasatinib, dasatino/nilotinib,
SRC	124	Dasatinib, dasatinib/erlotinib, dasatino/nilotinib, afatinib/dasatinib, SM1-71, ingenol mebutate (via STAT3)
PXN	48	SM1-71
JUN	30	
BRCA1	66	
P53	119	
MDM2	77	
CTNNB1	83	
CD44	13	
Multi-proteins		Ingenol mebutate

The hub proteins are ranked by their degree of connectivity within the network.

**Fig. 7:**
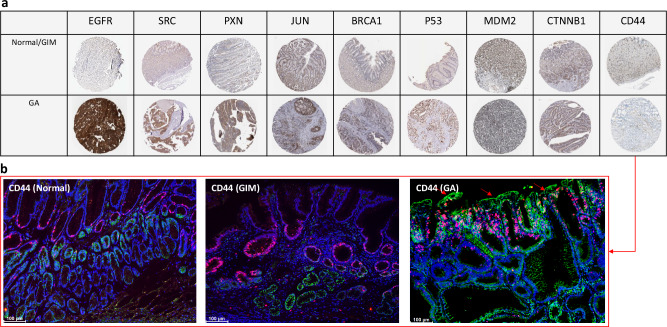
Representative immunohistochemistry of 9 hub proteins. Note: tissue microarray at Human Protein Atlas (**a**) and immunofluorescent staining (**b**) in which CD44 is indicated in green and Ki67 in red, and CD44 on goblet cells in (arrows). Bar = 100 μm.

Utilization of the Human Protein Atlas (v23.proteinatlas.org/)^39^ confirmed that the expression of the 8 out 9 proteins (except CD44) in both normal/GIM and GA tissue samples (Fig. 7a). Apparently, quantity of immunostaining was higher in GA ( < 75%) than GIM ( < 25% or 25–75%). Of note, tissue microarray data at the Human Protein Atlas didn’t show CD44 (Fig. 7a, Table S6). Thus, our immunohistochemistry showed that the expression of CD44 was expanded from basal part of mucosa of normal and GIM stomachs to upper part of mucosa of GA stomach (Fig. 7b).

The gene expression of these 9 proteins showed that PXN and CD44 were up-regulated in both GIM and GA (q < 0.001, *n* = 15), SRC and CTNNB1 were upregulated in GA but not in GIM (q < 0.001 for SRC and q = 0.05 for CTNNB1, *n* = 15), and JUN was upregulated in GIM but down-regulated in GA (q < 0.001, *n* = 15) in comparison with the normal tissues from the same stomachs (Table S6). Furthermore, scRNAseq showed these proteins (except BRCA1) were expressed in goblet cells in addition to other cell types (Table S6).

The molecular docking studies on FDA-approved compounds against EGFR, SRC, PXN, JUN, BRCA1, p53 (monomer and dimer), MDM2, CTNNB1 and CD44 demonstrated high binding affinities of these compounds to the respective proteins. After initial analysis, 15 compounds were selected for each protein based on their binding affinities followed by a structural analysis of the interaction between the protein of interest and selected components, including the biding affinity and interacting residues (length, number and type of bonds). Accordingly, top five compounds per target protein were chosen based on their binding affinity to further analyze their interactions and the nature of the bonds formed. PyMOL provided excellent visualization of these interactions, revealing a detailed and extensive array of bonds between the selected compounds and the amino acids of the target proteins (Table 3). Out of 1453 compounds, the five highest binding affinities for EGFR were obtained for E155, gliquidone, gossypol, troglitazone, ZINC3830383. In regard to SRC, the highest binding affinity was noticed in cases of ZINC3830342, ZINC3830343, ZINC3830371, ZINC3830384, and ZINC3830386. For PAX, accolate, defferin, troglitazone, ZINC1612996 and ZINC3830342 were found. For the case of Jun the highest binding affinities were obtained for differin, E155, ZINC3830369, rolapitant and ZINC1612996. In regard to BRCA1 the highest binding affinities were noticed for ZINC3830342, ZINC3830343, ZINC3830384, E155 and differin. The highest binding affinity in case of p53 dimer were obtained for E155, E155, ZINC607700, ZINC1612996, ZINC3830342 and ZINC3830384. When it comes to MDM2 the highest binding affinities were acquired for rolapitan, risperdal, accolate, ZINC3830371 and ZINC3830372. The highest binding affinity for CTNNB1 was obtained for ZINC3830383, E155, ZINC3830369, ZINC3830385 and ZINC3830386. For CD44 the highest binding affinities were acquired in cases of these molecules E155, ZINC3830342, ZINC3830383, and ZINC3830430 (Table 3). It should be noticed that ZINC3830342 and ZINC3830343 are relatively identical compounds with the same molecular characteristics, but with a slight difference in the structural formula, and therefore identical results are obtained (Table 3). Considering that it was a blind docking analysis, the active/binding sites were not taken into account when creating the gridbox. The listed compounds (E155, rolapitant, ZINC1612996 and ZINC3830369) bound and showed the highest binding affinity for a specific region, although interactions with similar residues were achieved, the number and length of hydrogen bonds were different.

**Table 3:** The hub proteins and ligand (repurposed drugs) interacting residues

Protein	Ligand	Binding affinity kcal/mol	Interacting residues
EGFR	E155 (ZINC3830332)	−10.3	Leu718; Gly719; Asp837 (3.7 Å); Arg841 (2.3 Å); Asn842, Ile878 (2.7 Å); Lys879 (2.3 Å) (four hydrogen bonds)
	Gliquidone (ZINC1482077)	−10.6	Gly762 (3.3 Å); Met766; Ala859 (3.5 Å); Leu861 (2.7 Å) (three hydrogen bonds)
	Gossypol (ZINC3775575)	−10.5	Gly721; Ala722 (3.1 Å); Gly724 (3.0 Å); Lys745 (2.0 Å); Met793 (3.4 Å) (four hydrogen bonds)
	Troglitazone (ZINC968276)	−10.5	Met793 (1.9 Å, 3.3 Å); Asp837 (3.3 Å); Arg841 (2.7 Å, 3.2 Å, 3.6 Å); Asn842 (2.0 Å) (forming a total of seven hydrogen bonds)
	ZINC3830383	−10.6	Lys745 (2.7 Å); Asp837 (3.4 Å, 3.8 Å); Arg841 (three hydrogen bonds)
SRC	ZINC3830342	−12.2	Met343 (3.8 Å); Gly346; Ser347 (3.3 Å); Ala392 (two hydrogen bonds)
	ZINC3830343	−12.2	Met343 (3.8 Å); Gly346, Ser347 (3.3 Å), Ala392 (two hydrogen bonds)
	ZINC3830371	−10.6	Arg162; Pro363 (3.5 Å); Asp367 (3.8 Å); Gly397 (3.4 Å); Asp399 (2.4 Å, 3.3 Å, 3.9 Å); Leu400 (2.0 Å), (seven hydrogen bonds)
	ZINC3830384	−11.6	Gln277 (3.4 Å); Cys279; Phe280 (3.0 Å); Lys297 (2.3 Å, 2.8 Å); Thr340; Ser347 (2.3 Å, 3.5 Å); Ala392 (3.7 Å), Asn393; Asp406 (3.3 Å, 3.9 Å) (nine hydrogen bonds)
	ZINC3830386	−11.5	Gln277 (3.4 Å); Gly278; Cys279 (2.0 Å); Lys297 (2.1 Å, 2.9 Å, 3.0 Å); Asp388 (3.4 Å); Arg390 (2.0 Å); Asn393; Asp406 (3.0, 3.9 Å) (nine hydrogen bonds)
PXN	Accolate (ZINC896717)	−5.9	Met616 (3.5 Å); Asp617 (3.4 Å, 4.0 Å); Asp618 (2.6 Å); Leu622 (3.4 Å); Asp625 (four hydrogen bonds)
	Differin (ZINC3784182)	−5.9	Leu622 (4.0 Å; Asp625 (3.3 Å) (two hydrogen bonds)
	Troglitazone (ZINC968276)	−5.9	Asp617 (3.7 Å); Asp618 (1.8 Å); Asp625 (two hydrogen bonds)
	ZINC1612996	−6.0	Leu623 (without forming hydrogen bonds)
	ZINC3830342	−5.9	Asp620 (without hydrogen bonds)
JUN	Differin (ZINC3784182)	−8.2	Arg272 (3.1 Å); Arg276; Glu281 (3.7 Å) (two hydrogen bonds)
	E155 (ZINC3830332)	−8.1	Ser269; Arg272 (2.1 Å, 3.6 Å); Glu275 (3.4 Å); Arg276 (2.6 Å); (four hydrogen bonds)
	Rolapitant (ZINC3816514)	−8.1	Ser269 (2.5 Å); Arg276 (2.6 Å) (two hydrogen bonds)
	ZINC1612996	−8.1	Arg272 (3.1 Å); Arg276; Glu281 (3.7 Å) (two hydrogen bonds)
	ZINC3830369	−8.1	Arg276 (2.2 Å, 3.3 Å); Glu281 (3.2 Å) (three hydrogen bonds)
BRCA1	ZINC3830342	−9.3	Trp1782; Gln1785 (without hydrogen bonds)
	ZINC3830343	−9.3	Trp1782; Gln1785 (without hydrogen bonds)
	ZINC3830384	−8.7	Sep406 (3.2 Å, 3.3 Å, 3.4 Å); Leu1657 (3.4 Å, 3.8 Å); Pro1659 (3.8 Å); Asn1678; Lys1702 (2.0 Å) (seven hydrogen bonds)
	E155 (ZINC3830332)	−9.1	Sep406 (3.3 Å, 3.5 Å); Leu1657 (3.2 Å, 3.7 Å); Thr1658 (3.5 Å), Arg1670; Asn1678 (2.8 Å) (six hydrogen bonds)
	Differin (ZINC3784182)	−8.6	Gln1811 (2.6 Å); Arg1835 (2.2 Å); Glu1836 (3.7 Å) (three hydrogen bonds)
p53 (dimer)	E155	−7.7	Gln100 (3.1 Å); Ala138 (3.4 Å); Thr140 (3.1 Å); Ser166 (3.1 Å, 3.4 Å); Asp186; Arg196 (1.9 Å); Glu198 (3.8 Å); Asn235 (3.7 Å) (eight hydrogen bonds)
	ZINC607700	−10.6	Lys164 (3.9 Å); Asp186 (3.4 Å); Arg196 (2.7 Å) (three hydrogen bonds)
	ZINC1612996	−10.5	Gln100 (2.1 Å); Lys164 (3.8 Å); Ser166 (2.0, 2.8 Å); Val197 (3.8 Å); Gly199 (2.5 Å); Asn200 (1.9 Å); Asn235 (3.8 Å) (eight hydrogen bonds)
	ZINC3830342	−10.6	Thr150; Asp228 (without hydrogen bonds)
	ZINC3830384	−10.4	ZINC 3830384 -10,4 - Leu137 (3.4 Å), Lys139 (2.2 Å), Ser166 (2.3, 2.6, 2.8 Å), Gln167, Asp186 (3.5 Å), Arg196 (2.1, 3.1 Å), Glu198 (3.4 Å) (nine hydrogen bonds)
MDM2	ZINC3830371	−10.2	Gln72 (3.2 Å, 3.4 Å, 3.6 Å); His96 (3.0 Å); Ile99; Tyr100 (four hydrogen bonds)
	ZINC3830372	−10.4	Gln18 (3.3 Å); Ile19 (2.2 Å, 3.6 Å); Gln24 (2.7 Å); Val93 (3.9 Å); His (2.1 Å) (six hydrogen bonds)
	Accolate ZINC896717	−10.3	Gln24, Leu54 (3.5 Å); His96 (one hydrogen bond)
	Risperidal ZINC538312	−10.6	Ile19 (3.5 Å); Pro20; Gln24 (2.4 Å); Leu54 (3.2 Å); Val93 (three hydrogen bonds)
	Rolapitant ZINC3816514	−10.7	Leu54 (3.5 Å) (one hydrogen bond)
CTNNB1	ZINC3830383	−9.2	His223, His260, Asn261, Lys292, Asp295, Asp299, Tyr333, Thr339, Arg342
	E155	−9.1	Asp249, Ser250, Thr289, Asn290
	ZINC3830369	−8.9	Asn290, Asp299, Thr339, Arg342
	ZINC3830385	−8.6	Gln266, Gly268, Ala269, Lys270, Met271, Tyr306, Gly307, Asn308
	ZINC3830386	−8.6	His260, Ala295, Asp299, Lys335, Thr339, Arg342
CD44	E155 (ZINC3830332)	−9.2	Lys42 (2.2 Å, 2.9 Å); Arg45 (1.8 Å, 3.3 Å); Tyr46 (3.1 Å, 3.7 Å); Ser47; Ser117 (2.1 Å); His118 (2.6 Å, 2.7 Å); Arg167 (2.5 Å); Asp172 (3.7 Å) (11 hydrogen bonds)
	Irinotecan (ZINC1612996)	−9.5	Asn29; Glu41 (3.6 Å); Glu79 (3.4 Å); Arg155 (two hydrogen bonds)
	ZINC3830342	−9.9	Glu41; Cys81 (2.4 Å) (one hydrogen bond)
	ZINC3830383	−9.5	Arg33 (2.9 Å); Phe60 (3.8 Å); Asn125 (1.9 Å, 2.4 Å, 3.4 Å, 3.6 Å); Ser127 (2.4 Å, 2.7 Å, 3.5 Å); Thr138 (nine hydrogen bonds)
	ZINC3830430	−9.3	Asn29 (2.9 Å, 3.6 Å); Val30; Thr31; Tyr34 (2.1 Å); His39 (3.0 Å); Glu79 (3.8 Å); Arg94 (1.9 Å, 2.2 Å, 2.6 Å); Asn (2.1 Å); Arg155 (nine hydrogen bonds)

## Discussion

Biomarkers are needed in risk assessment, screening, differential diagnosis, determination of prognosis, prediction of response to treatment, and monitoring of progression from GIM to GA^40,41^. Particularly, the biomarkers specifically tailored for GA-related GIM could serve as an ideal tool in establishing personalized endoscopic surveillance programs and treatments.

It has been proposed that the incomplete GIM by pathological evaluation might be useful as a biomarker for GA-related GIM, as there is an association between the incomplete GIM and GA in comparison with the complete GIM. Some facilities (particularly in Japan) have recently attempted to perform Endoscopic Grading of Gastric Intestinal Metaplasia such as the Operative Link on Gastric Intestinal Metaplasia Assessment (OLGIM)^42–44^. However, it is practically a challenge due to economic concern, invasiveness to the patients and complex task against clinical daily task flow. The literature also shows high activities in identified potential biomarkers of incomplete GIM^45,46^. In the present study, our aim was not to identify biomarkers for complete *vs* incomplete GIM rather than to identify biomarkers of GA-related GIM. Accordingly, the study subjects were GA patients and the study samples of GIM and GA were collected within the same stomachs of GA patients.

By creating GEM based on pathological diagnosis and single cell atlas, we were able to show ‘the gene expression profile’ for the individual patients or subgroups of patients. However, it was difficult to visualize specific clusters of genes for GIM-related GA within the user’s knowledge domain. It would be possible in the future if powerful visual analytics can be developed, e.g. on linking a reordered GEM heatmap and dual 2D projections of its rows and columns, which can be recomputed conditioned by subsets of genes and/or samples selected by the user during the analysis^47^.

We further utilized our computational platform ScType (developed by AI et al. ^30^) to create UMAP plot and found that the gene expression by “cancer cells” and immune cells (such as dendritic cells, B cells, CD8 + NKT-like cells and mast cells) appeared to be lowest in GA than non-dysplastic lesions including chronic atrophic gastritis, GIM and non-atrophic gastritis. Presumably, the inflammation in the tumor microenvironment suppressed the gene expression by the cancer cells, particularly at the early stage. It was unexpected, as it has been well documented that inflammation predisposes to the development of cancer and promotes all stages of tumorigenesis^48^. It was also unexpected, as it is known that chronic inflammation facilitates tumor progression, whereas acute inflammatory may stimulate the maturation of dendritic cells and antigen presentation, leading to anti-tumor immune responses^49^, and the tumor-promoting inflammation may be regarded as the host’s defense against malignancy^50^.

Furthermore, we found RBP2 expression in GIM but not in GA. Retinoblastoma binding protein 2 (RBP2), also known as cellular RBP2 (CRBP2), belongs to the family of intracellular lipid-binding proteins known as fatty acid-binding proteins (FABPs). Several reports showed that RBP2 stimulated various processes such as HIF-1α–VEGF-induced angiogenesis in non-small cell lung cancer through the Akt pathway^51^, malignant progression of GA through the TGF-β1-(p-Smad3)-RBP2-E-cadherin-Smad3 feedback circuit^52^, induction of stem-like cancer cells by promoting epithelial-mesenchymal transition in renal cell carcinoma^53^, and initiation of ER and IGF1R-ErbB signaling in tamoxifen resistance in breast cancer^54^. However, it should be kept in mind that attributing a single gene as a biomarker would be risky, considering that GA is neither a monogenic nor a polygenic malignancy.

By concentrating on pathways as potential biomarkers for GA-related GIM, we used Ingenuity Pathways Analysis (IPA) to create the bar graphs consisting of 110 canonical signaling pathways. We found that while the activated signaling pathways in GIM might be deemed biomarkers for GA, some of these pathways were inactivated in GA when compared with GIM. Thus, more attention should be given to the dynamic changes in the progression from GIM to GA. Furthermore, we found that it was a moderate negative correlation between GIM and GA, which was unexpected as GIM is believed to be the primary precancerous lesion in GA tumorigenesis. As shown in the literature, only some GIM, but not all, is considered to be the precursor of gastric cancer^55^. In the present study, more samples and patients are needed for establishing the topologic association and more importantly the causality between GIM and GA in the future.

It should be kept in mind that association should not be confused with causality; if GIM causes GA, then the two are associated (dependent). However, associations can arise between variables in the presence (i.e., GIM causes GA) and absence (i.e., they have a common cause) of a causal relationship^56^. By performing the functional enrichment analysis which was based on our data in combination with public available databases, we have created the biomarker network and the network of signaling pathways of GA-related GIM. We found Wnt/β-catenin pathway to be one of the most important biomarkers for GA-related GIM. This was in line with the literature of Wnt signaling in regulating neoplastic transformation and malignant growth, including GA^33,37,38^. By additional functional annotation and validation analysis (e.g. immunohistochemistry), we found gastric stem cell marker CD44 as GA-related GIM. These findings may support the notion that GIM is the primary precancerous lesion in GA tumorigenesis, as both share the “cause” (i.e., hyperactivity of Wnt signaling and the presence of tissue-resident stem cells)^32–34^. Wnt signaling is often implicated in stem cell control as a proliferative and self-renewal signal. Mutations in Wnt genes or Wnt pathway components lead to specific developmental defects, while various diseases, including cancers, are caused by abnormal Wnt signaling^57^. Although molecular targeting of the Wnt signaling system has been proposed and trialed for the treatment of various cancer types without significant success yet^58,59^, Wnt signaling, particular CD44, should be potential target in the future.

In addition to drug targeting on individual hub/key genes and proteins that are highly expressed in GA-related GIM, we should consider the key nodes that keep the stability of network as potential targets. By topological analysis, we found several potential nodes for GA-related GIM, e.g., apoptosis (such as cytochrome c-mediated apoptosis and laminin interactions and apoptosis) and intracellular trafficking (such as COPI-mediated anterograde transport, membrane trafficking, vesicle-mediated transport). Indeed, dysregulation of apoptosis has been well recognized as a hallmark of cancer cells due to mutations in the extrinsic, intrinsic, p53, and the related signaling pathways. Accordingly, current efforts have been made to develop agents that target apoptotic pathways either directly or indirectly^60^. Targeting membrane trafficking particularly in connection with Wnt signaling has also been proposed as a strategy for cancer treatment^61^.

The results of the present study by the topological analysis revealed the nervous system development and axon guidance as potential key node in GA-related GIM, which was in line with our previous studies that demonstrated the role of vagal nerve in gastric tumorigenesis^33,62^. Another key node was RAS/RAF/MAPK pathway. Indeed, RAF inhibitors (RAFi) combined with MEK blockers have been taken as an FDA-approved therapeutic strategy for numerous RAF-mutant cancers, including melanoma, non-small cell lung carcinoma, and thyroid cancer^63^. It would be of interest to include GA-related GIM as a prevention strategy.

We utilized IPA analysis of PPI and identified the hub proteins including EGFR, SRC, PXN, Jun, BRCA1, TP53, MDM2 and CTNNB1. CD44 was included not because of its PPI but the biological importance in GIM-related GA as forementioned. The hub proteins were initially identified by deploying the Causal Network Analysis, enabling us to identify master regulators (hubs) that act directly upon other dataset molecules, or through one or more intermediate upstream regulators. The analysis was performed using data from gene expression profiling and then validated by tissue microarray (i.e., immunohistochemistry) that are publicly available at the Human Protein Atlas and immunofluorescence for CD44. Furthermore, by performing drug-hub protein targeting interactions, we identified the following potential repurposed drugs for treatments of GA-related GIM.

Dasatinib is a tyrosine kinase inhibitor (Sprycel; Bristol-Myers Squibb) and has been used for the treatment of chronic myeloid leukemia and Philadelphia-chromosome-positive acute lymphoblastic leukemia^64^. Recent studies suggest an alternative mechanism of action for dasatinib, which involves augmenting the population of functionally active CAR T cells^65^. SM1-71 is a multitargeted kinase inhibitor that includes MKNK2, MAP2K1/2/3/4/6/7, GAK, AAK1, BMP2K, MAP3K7, MAPKAPK5, GSK3A/B, MAPK1/3, SRC, YES1, FGFR1, ZAK (MLTK), MAP3K1, LIMK1, and RSK2^66^. JNJ-26483327 is a multitarget tyrosine kinase inhibitor^67^. Adapalene, also known as CD271 and differin, belongs to the 3rd generation of retinoids that were approved by the Food and Drug Administration (FDA) in 1996 to treat acne vulgaris. A recent study using in silico, in vitro, and in vivo models suggested that adapalene could be used for treatment of multiple myeloma and Leukemia^68^. Irinotecan liposome has been approved by FDA on Feb. 13, 2024 as first-line treatment of metastatic pancreatic adenocarcinoma (https://www.fda.gov/drugs/resources-information-approved-drugs/fda-approves-irinotecan-liposome-first-line-treatment-metastatic-pancreatic-adenocarcinoma). Gliquidone has been widely used for the treatment of type 2 diabetes. There was no report yet for treatment of cancer but possibly for neuroinflammatory disease^69^, which may share some common features with GIM as shown in this study. Gossypol, also called AT-101, has been tested in clinical trials as a single agent or in combination with standard therapy for various cancer types, showing a trend toward increased overall survival and progression-free survival^70^. Troglitazone as a PPARγ ligand has been reported to exhibit antiproliferative effects and/or enhance cancer immunotherapy^71,72^. Other drugs, such as rolapitant that is used as an antiemetic in oncology^73^, accolate for chronic treatment of asthma, and risperidal for mental/mood disorders, and other ligands shown in Table 3 will be of interest in searching for repurposing to treat GIM.

Computational molecular docking has been used as a tool for the discovery of repurposed drugs^74,75^. By the docking analysis with PyMOL, we identified and visualized five compounds per target protein based on their binding affinity, the nature of the bonds formed and interactions. We presented the repurposed drugs and believed that the information will be useful not only for the identified drugs but also for development of new drugs as potential ‘prototypes’, particularly for CD44 which has the highest binding affinities with the following molecules including E155, ZINC3830342, ZINC3830383, and ZINC3830430.

Taken together, the methods and the results of this study could help in the targeted selection of GIM individuals who would benefit the most from screening and surveillance endoscopy and in the development of treatment for GA-related GIM. The multi-bioinformatics included GEM, ScType on scRNA-seq, IPA, Cytoscape, tissue microarray at HPA, Gene set enrichment analysis (GSEA) (also called functional enrichment analysis or pathway enrichment analysis) and molecular docking analysis, in addition to preliminary analyses with pagerank centrality, GO and MSigDB enrichments (data not shown).

This study has at least the following four limitations. First, after identifying potential biomarkers, the next step is validation. One of possible in vivo models would be the INS-GAS mice in which the majority are with high risk of spontaneous progression from GIM to GA^76^. Unfortunately, we didn’t have capacity at the time of validating the repurposed drugs as reported in the present study. Furthermore, we didn’t find the individual specific biomarkers that were correlated with the spatial progression of GIM to GA in terms of express levels and grade of dysplasia, and we were unable to identify the individual specific biomarkers that were upregulated in patients with nondysplastic GIM that later progress to GA. Therefore, we proposed the GEM, signaling pathways, molecular network and network of signaling pathways. Presumably, this study (together with other studies in the literature) might contribute to the future development of AI/ML-supported in silico models in prediction of the progression of GIM patients to GA. Second, the repurposed drugs proposed in this study should be assessed in detail in terms of risk-benefit analysis. Many of the drugs that were listed in the present study are fairly toxic and would not be recommended (either alone or in combinations) unless the risk of GA progression is extremely high. However, it would be possible to take these repurposed drugs and other molecular ligands as “lead compounds or prototypes” for the drug development for treatment of GA-related GIM. Third, based on the results of this study and the literature, we proposed a hypothesis that GIM and GA share the common tissue-resident stem cells, leading to the progression from GIM to GA. One of possible validation methods could be organoid models of GIM *vs*. GA. Forth, the interpretations of the results reported in the present study (including main and supplementary results) were limited due to consideration of the length of Discussion.

## Methods

### Patients and study design

**Fig. 8:**
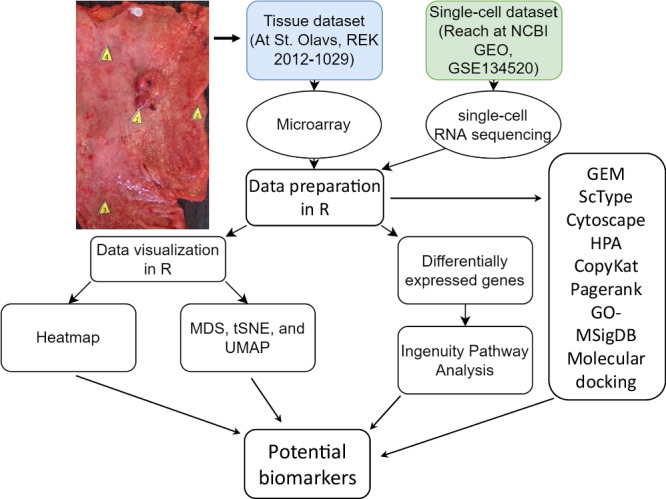
Study design showing multi-bioinformatics used for analysis of surgical biopsies (4 samples per stomach = total 60 samples from 16 patients), single-cell RNA sequencing from 13 patients^91^, immunohistochemistry from 19 patients.

This study has been performed in accordance with the Declaration of Helsinki and approved by the Regional Committees for Medical and Health Research Ethics Central Norway (REK 2012-1029). The surgical samples were taken from 16 GA patients at St. Olav’s Hospital. The stomach specimens were taken from four pre-determined positions in corpus (major and minor curvature), cardia and antrum per stomach (Fig. 8), including tumors, adjacent non-tumor tissues, and distanced non-tumor tissues, immediately after gastrectomy during 2005 to 2010. All patients were diagnosed histologically as primary gastric adenocarcinoma of stage I-IV. Patients have since been followed-up for five years at St. Olavs Hospital, Trondheim, Norway. TNM status was defined, and samples were classified according to Lauren’s classification, (Intestinal, diffuse or mixed/combined type), WHO classification (tubular, papillary, mucinous and poorly cohesive), WHO grading (well, moderately or poorly differentiated). All histological samples without ones from one patient (no. 6) due to a technical error were reviewed according to the Japanese pathological classification. Samples were assigned gastric histopathology scoring including inflammation, epithelial defects, oxyntic atrophy, epithelial hyperplasia and dysplasia and an overall GHAI score. The samples from patient no. 6 did not undergo pathological evaluation and were not analyzed further. Thus, 15 patients were included for gene expression profiling. One or more of the four tissue samples displayed the tumor, and the remaining tissue samples showed a “normal” appearance. The samples were then evaluated histologically and subjected to Illumina microarray gene expression. In addition, the tissue biopsy samples were obtained as routine medical diagnosis by endoscopy from patients with gastritis and/or gastric cancer and were subjected to pathology assessment. Total eight samples from eight patients were used for the immunofluorescent staining of CD44 at Tokyo University Hospital, and the three representative samples were used in this study. Ethics protocol was approved at Tokyo University Hospital and written informed consent was obtained from all patients.

### Multi-bioinformatics

Illumina microarray gene expression data were extracted as an Excel document, while the control data results were extracted. Data wrangling and normalization were performed with R version 4.1.1 and the tidyverse package version 1.3.1. The data frame was filtered using the sample vector list containing the patient samples that were not used for further analysis and was then saved as a text file to be imported into lumi. The control data were similarly imported from a text file to the data frame of control before it was filtered and saved as a text file. Lumi version 2.44.0 was used to filter the unexpressed genes from the data. Genes with detection threshold < 1% in 10 or fewer samples were removed. A limit of 11 was set because the smallest group is GIM, which contains 11 samples. The patient pathology was imported as pdf (pathology dataframe) and filtered to be used as a design matrix. Any flawed samples were removed before the samples were filtered and categorized as GA, GIM or normal. Samples were categorized as cancer if they had a pathological evaluation as GA or a biopsy classification of 2 out of 5, where a biopsy classification of 1 is certain tumor tissue. Samples were categorized as GIM if they were not classified as GA but had a histopathological scoring that indicated intestinal metaplasia. Samples were categorized as normal if they were neither classified as GA nor GIM. After categorization, a new column concerning the sample pathology was made.

A single-cell transcriptomic atlas of premalignant lesions and early GA was modified for data visualization and analysis. All genes that had an adjusted p value above 5% were removed. Genes found in more than one cell type were removed when using the single-cell transcriptome biomarkers as cell type biomarkers for the tissue data. In this way, only the RNAs that were not significant in any other cell type would be used when making a heatmap or when utilizing cell type-specific clustering. Genes without a match in the sample data frame were cross-referenced with the UniProt knowledgebase. The genes from the single-cell transcriptomics were then sorted to coincide/align with the microarray data. The data of the single-cell transcriptomic atlas were also used to confirm the findings by using two different datasets in Seurat version 4.0.4. Cells expressing less than 400 genes, more than 7000 genes or cells containing more than 20% genes correlated to the mitochondria were filtered out.

The housekeeping genes *CTBP1, CUL1, DIMT1L, FBXW2, GPBP1, LUC7L2, OAZ1, PAPOLA, SPG21, TRIM27, UBQLN1, ZNF207, AGPAT1, B2M, CAPN2, CYCC (CCNC), PMM1, SDHA, RPL29, RPL29-B2M*, and *B2M-GAPDH* were used for normalization using geNorm, a computional method, from ctrlGene version 1.0.1. It should be noted that OAZ1 was removed from the normalization due to high intensity output. SPG21 was removed from the normalization due to a very low expression value. The data were also normalized with DESeq2 version 1.32.0. (It should be mentioned that the effects of different normalization methods on the outcomes were not included in the present study).

Seurat version 4.0.4 was used to perform dimensional reduction. Normalized data from the patients were inputted into Seurat and scaled, clustered and plotted through Seurat functions: CreateSeuratObject, ScaleData, FindVariableFeatures, RunPCA, FindNeighbors, FindClusters, RunUMAP and RunTSNE. The clustering methods performed were UMAP and tSNE. Important parameters included 1) selection.method = vst, 2) nfeatures = 5000, 3) dims = 1:10 and 4) perplexity = 10. Heatmaps were created by ComplexHeatmap version 2.8.0. The differential equations were made with both DESeq2 and limma. The single-cell transcriptomic atlas was tested in DESeq2, whereas the microarray data were tested in both DESeq2 and limma.

The tissue and single-cell transcriptome datasets and their differentially expressed genes (DEGs) were imported into IPA (Qiagen). The microarray data mapped 1 9223 of 2 0918 gene IDs, and the single-cell transcriptome mapped 17921 if 22910 gene IDs to equivalent IDs in IPA. A core analysis was then run on each dataset with the criteria of an adjusted *p* value < 0.05 and an absolute log2FoldChange of 1. Each dataset was considered for their signaling pathways. A comparison analysis was performed between the scRNA-seq and the background-corrected lima processed dataset.

A co-analysis of all patient samples was performed with ScType to visualize cell populations and the cell type abundance for each tissue type^30^. To define the subset of cancerous cells, aneuploidy analysis was performed using CopyKat. The underlying idea is that gene expression levels of many adjacent genes can provide depth information to infer genomic copy number in that region, and cells with extensive genome‐wide copy number aberrations (aneuploidy) are considered cancer cells. In the case of the raw fastq files or BAM file, real variant calling was performed to validate the subset of cancer cells in consideration of CopyKat, which is well correlated with SNV calling, especially for solid tumors^30^. Furthermore, to investigate latent disease‐related regulatory changes that are invisible based on clustering or differential expression analysis, regulatory networks from single-cell data and quantify gene centralities were inferred. PageRank centrality was calculated to identify the nodes with high Pagerank centrality, indicating “popular” genes involved in multiple regulatory pathways. GO and MSigDB enrichments were performed to visualize overrepresentation of KEGG_CELL_ADHESION_MOLECULES_CAMS^77^. Network graph was built using igraph v1.4.3 and visualized with ggplot2 v3.3.6.

Functional enrichment analysis was performed using Protein-Protein Interaction Networks (https://string-db.org/), Protein, Genetic and Chemical Interactions (https://thebiogrid.org/) and IntAct Molecular Interaction Database (https://www.ebi.ac.uk/intact/home) and Cytoscape 3.10.

The Human Protein Atlas was used, including pathology data with immunohistochemisty using semi-quantitative tissue microarrays based on The Human Protein Atlas version 23.0 and Ensembl version 109 and RNA single cell read count data based on 31 datasets (https://www.proteinatlas.org/)^39,78–84^.

Blind docking analysis was performed using 100 random compounds from the FDA-approved drug list. The three-dimensional crystal structures of target proteins were retrieved from the RCSB Protein Data Bank (PDB) in PDB format^85^. These proteins include the epidermal growth factor receptor (PDB ID: 5U8L), proto-oncogene tyrosine-protein kinase Src (PDB ID: 8XN8), paxillin (PDB ID: 5W93), transcription factor Jun (PDB ID: 5T01), breast cancer type 1 susceptibility protein (PDB ID: 4Y2G), tumor protein p53 (PDB ID: 8A31), E3 ubiquitin-protein ligase MDM2 (PDB ID: 4MDN), and CD44 antigen (PDB ID: 5SC3). Initially, these structures were in complex formations with other molecules. To facilitate further analysis, protein preparation was performed using AutoDock Tools 1.5.6^86^, which involved removing water molecules, adding polar hydrogen atoms, and assigning Kollman charges. Following these modifications, the structures of the target proteins were converted from PDB to PDBQT format. Additionally, AutoDock Tools 1.5.6 was utilized to define a grid box for each protein, setting the stage for subsequent docking simulations. The grid box dimensions for EGFR were 48 ×49 x 61 Å and it was centered at 5.549, -6.743, and -25.974. For SRC the dimensions were set to 64 ×62 x 66 Å and it was centered at 23.298, 0.183, and 15.596. For PAX the grid box dimensions were 24 ×24 x 24 Å, centered at 3.757, -7.759 and 32.776. The grid box dimensions for Jun were 68 ×70 x 68 Å and it was centered at -16.564, 21.969 and 24.614. For BRCA 1 the dimensions were 68 ×50 x 40 Å, centered at -26.133, 15.257 and -27.353. For p53 the grid box dimensions were 40 ×48 x 50 Å, centered at 98.225, 80.184 and -29.347. The grid box dimensions for MDM2 were 36 ×34 x 44 Å and it was centered at -14.982, 0.643 and 1.031, while for CD44 the dimensions were set to 36 ×48 x 40 and it was centered at 0.933, -0.201 and 5.715. For each analysis the value of spacing (ångstrom) was set to 1.0. Three-dimensional structures of FDA approved drugs were available in the ZINC database^87^. Compounds were initially downloaded in the appropriate SDF format and then prepared and converted to PDBQT format using OpenBabel 3.1.1 software with default settings. During this preparation phase, various adjustments were applied to the ligands, including the addition of charges, hydrogen atoms, assignment of atom types, conversion of bond types, and establishment of the root. It is important to highlight that the default settings of OpenBabel generate the molecule’s 3D structure in a neutral state without considering any ionization states, a feature maintained in the PDBQT format of the ligands employed in this study^88^. Molecular docking simulations were conducted utilizing AutoDock Vina 1.1.2, employing an energy range of four and an exhaustiveness value of 32^89^. The interactions between the receptors and the chosen compounds were subsequently visualized and analyzed using PyMOL 2.5.4^90^.

## Supplementary information

Supplemental Information

## Data Availability

All data used in this study are available within the manuscript and the raw data will be provided per request.
