# Supplementary material for: Multi-bioinformatics revealed potential biomarkers and repurposed drugs for gastric adenocarcinoma-related gastric intestinal metaplasia
Source: NPJ Syst Biol Appl. 2024 Nov 4;10:127. doi: 10.1038/s41540-024-00455-0 (PMC11535201; doi:10.1038/s41540-024-00455-0)
Supplement: Supplementary file 1 — Supplemental Information [file 41540_2024_455_MOESM1_ESM.pdf]

npj Systems Biology and Applications

Multi-bioinformatics revealed potential biomarkers and repurposed drugs for gastric adenocarcinoma-related gastric intestinal metaplasia

Gøran Troseth Andersen[1,2,3], Aleksandr Ianevski[1], Mathilde Resell[1], Naris Pojskic[4], Hanne-Line Rabben[1], Synne Geithus[1], Yosuke Kodama[1], Hiroyuki Tomita[5], Denis Kainov[1], Jon Erik Grønbech[1,2], Yoku Hayakawa[6], Timothy Wang[7], Chun-Mei Zhao[1] and Duan Chen[1§]

[1] Department of Clinical and Molecular Medicine, Norwegian University of Science and Technology (NTNU), Norway
[2] Department of Surgery, St. Olav's Hospital, Norway
[3] Department of Surgery, Namsos Hospital, Norway
[4] Laboratory for Bioinformatics and Biostatistics, University of Sarajevo - Institute for Genetic Engineering and Biotechnology, Bosnia and Herzegovina
[5] Department of Tumor Pathology, Gifu University Graduate School of Medicine, Japan
[6] Department of Gastroenterology, Tokyo University Hospital, Japan
[7] Department of Digestive and Liver Diseases and Herbert Iring Comprehensive Cancer Center, Columbia University Medical Center, USA

§ Corresponding to Duan Chen, Professor, Gastro Center of St. Olavs Hospital, Prinsesse Kristinas gate 1, 7030 Trondheim, Norway

duan.chen@ntnu.no

SUPPLEMENTARY INFORMATION FILE:

Table S1	Signaling pathways of GIM and GA	page 2-4
Table S2	865 GA-related GIM proteins	page 4-23
Table S3	% genes/term of GA-related GIM	page 24-58
Table S4	Topological coefficient	page 59-64
Table S5	Hub proteins with PPI >3 in GA-related GIM	page 65-72
Table s6	Hub protein immunohistochemistry and RNA expression	page 73-75

Table S1: Pathways	GIM	GA
Insulin Receptor Signaling	-0.25	0
Pyridoxal 5`-phosphate Savage Pathway	-0.27	0
UDP-N-acetyl-D-galactosamine Biosynthesis II	0	-0.28
Glucose and Glucose-1-phosphate Degradation	0	-0.28
Geramide Degradation	0	-0.28
Acute Myeloid Leukemia Signaling	0	+0.38
Blader Cancer Signaling	0	+0.15
PTEN Signaling	+0.27	-0.27
Dopamine Receptor Signaling	0	-0.57
Remodeling of Epithelial Adherens Junction	0	-0.52
BMP Signaling Pathway	0	-0.69
UVC-induced MAPK Signaling	+0.31	-0.69
Glioma Signaling	+0.28	-0.41
AMPK Signaling	-0.56	-0.07
Inhibition of Angiogenesis by TSP1	-0.42	-0.15
Estrogen-Dependent Breast Cancer Signaling	-0.59	-0.49
Amyloid Processing	-0.42	-0.42
Regulation of Cellular Mechanics by Calpain Protease	+0.80	+0.28
Ovarian Cancer Signaling	+0.74	+0.28
Pancreatic Cancer Signaling	+0.80	+0.27
Neuregulin Signaling	+0.81	+0.14
PhoA Signaling	+0.42	0
STAT3 Pathway	+0.56	0
IL-22 Signaling	+0.42	+0.15
Leukotriene Biosynthesis	+0.77	0
Rac Signaling	+0.79	+0.56
MIF Regulation of Innate Immunity	+0.83	+0.49
Fcγ Receptor-Mediated Phagocytosis in Macrophages and Monocytes	+0.56	+0.53
Regulation of elF4 and p70s6k Signaling	+0.56	+0.67
Salvage Pathways of Pyrimidine Ribonucleotides	+1.12	+0.65
NF-κB Signaling	-0.97	+0.96
Apoptosis Signaling	-0.96	+0.85
HIPPO Signaling	-1.12	+0.83
Small Cell Lung Cancer Signaling	-0.81	+0.59
B Cell Activating Factors Signaling	-0.98	+0.35
P53 Signaling	-0.96	+0.14
April Mediated Signaling	-0.98	0
IL-6 Signaling	0	0
Oncostatin M Signaling	0	+1.92
Chondroitin Sulfate Degradation (Metazoa)	0	+1.05
ATM Signaling	-0.25	+1.08
Folate Polyglutamylation	0	+0.78
Telomerase Signaling	-0.25	+0.77
IL-17A Signaling in Gastrin Cells	0	+0.42

Glycogen Degradation II	0	+0.56
HGF Signaling	+0.28	+0.56
ERK/MAPK Signaling	+0.35	+1.09
Wnt/β-catenin Signaling	+0.42	+1.12
P38 MAPK Signaling	+0.44	+1.01
Role of BRCA1 in DNA Damage Response	+4.34	+1.16
Sirtuin Signaling Pathway	+0.82	+1.26
Death Receptor Signaling	0	+1.89
Purine Nucleotides De NOVO Biosynthesis II	0	+1.83
Acute Phase Response Signaling	0	+1.82
Interferon Signaling	0	+2.08
Tetrahydrofolate Salvage from 5,10-methenyltetrahydrofolate	0	+1.65
Cell cycle Regulation by BTG Family Proteins	0	+1.54
Mitosis Roles of Polo-like Kinase	0	+1.68
Type 1 Diabetes Mellitus Signaling	+0.28	+1.96
Aging Interaction at Neuromuscular Junction	+0.26	+1.51
Guanosine Nucleotides Degradation	+1.96	0
Mevalonate Pathway	+1.96	0
Acyl-coA Hydrolysis	+1.96	0
Sucrose Degradation V(Mammalian)	+1.83	0
Cholesterol Biosynthesis II (via 24,25-dihydrolanosterol)	+2.24	0
Cholesterol Biosynthesis III (via Desmosterol)	+2.24	0
Cholesteril Biosynthesis I	+2.24	0
Citrulline Biosynthesis	+2.24	0
Superpathway of Citrulline Metabolism	+2.66	0
Stearate Biosynthesis I (Animal)	+2.52	-0.25
NRF2-Mediated Oxidative Stress Response	+1.59	+0.40
γ-glutamyl Cycle	+1.61	+0.40
CDK5 Signaling	+1.54	+0.41
D-myo-inositol (1,4,5)-tris phosphate Degradation	+1.60	+0.28
Ceramide Signaling	+1.57	+0.25
Glycolysis	+2.05	+0.25
D-myo-inositol-5-phosphate Metabolism	+1.76	+0.25
Glycogen Degradation	+1.96	+0.56
γ-linolenate Biosynthesis II (Animal)	+1.33	0
IGF-1 Signaling	+1.26	0
14-3-3-Mediated Signaling	+1.47	0
CD27 Signaling in Lymphocytes	+0.98	-0.37
Superpathway of D-myo-inositol(1,4,5)-tris phosphate Metabolism	+1.40	-0.54
D-myo-inositol(1,4,5,6)-tetrakisphosphate Biosynthesis	+1.75	+1.12
D-myo-inositol(3,4,5,6)-tetrakisphosphate Biosynthesis	+1.12	+1.12
3-phosphoinositide Degradation	+1.67	+0.91
Ephrin Receptor Signaling	+1.33	+1.19
mTOR Signaling	+1.33	+1.26

Induction of Apoptosis by HIV I	+1.24	+1.55
Pyrimidine Ribonucleotides De NOVO Biosynthesis	+2.27	+1.44
Pyrimidine Ribonucleotides Interconversion	+2.26	+1.28
Apelin Liver Signaling Pathway	+1.96	+1.96
IL-8 Signaling	+1.54	+2.38
2-oxobutanoate Degradation	0	-1.61
Methylmalonyl Pathway	0	-1.61
Leucine Degradation	0	-1.54
Oleate Biosynthesis II (Animal)	0	-1.54
Complement System	0	-1.68
Role of CHK Proteins in Cell Cycle Check-point control	+0.31	-1.40
Aldosterone Signaling in Epithelial Cells	+0.71	-1.96
Gluconeogenesis I	+1.38	-0.96
Cell cycle: G2/SM DNA Damage Checkpoint Regulation	+1.41	-0.98
Phenylalanine Degradation IV (Mammalin, via Side Chain)	+1.27	-1.26
Cell Cycle: G1/S Checkpoint Regulation	+0.84	-1.12
D-myo-inositol(1,3,4)-trisphosphate Biosynthesis	+0.81	-0.84
Methylglyoxal Degradation III	+1.96	-1.68
Valine Degradation	+2.02	-1.53
Glutathione-Mediated Detoxification	+1.61	-1.68
Colanic Acid Buolding Block Biosynthesis	+1.96	-1.26
Bile Biosynthesis, Neural Pathway	+2.24	-1.26
Acid Fatty Acid B-Oxidation	+2.63	-1.54
Bupropion Degradation	+2.66	-1.82
Aceton Degradation (to Methylglyoxal)	+2.92	-1.68
Thyroid Hormone Metabolism II (via Conjugation and/or Degradation)	+3.36	-1.26
Glutaryl-coA Degradation	+2.24	-0.84
Superpathway of Cholestrol Biosynthesis	+2.94	-0.56
Granzyme B Signaling	0	+2.66
P13K/AKT Signaling	0	+2.66
Inflammasome Pathway	0	+2.73
GP6 Signaling Pathway	+0.72	+2.66
TREM 1 Signaling	0	+3.36
NER Pathway	-0.68	+1.75
EIF2 Signaling	-1.12	+1.67
Estrogen-Mediated S-Phase Entry	-0.99	+2.45
Cycling and Cell Cycle Regulation	-1.05	+2.52
Dematan Sulfate Degradation (Metaz)	-1.96	+0.70
LPS/IL-1- Mediated Inhibition of RXR Function	-2.38	+0.91
Ary Hydrocarbon Receptor Signaling	-1.54	+0.98
Toll-like Receptor Signaling	-2.1	+1.64
Nicotine Degradation III	+3.71	-2.31
Nicotine Degradation II	+3.85	-2.52
Estrogen Biosynthesis	+3.43	-2.03
Superpathway Of Melatonin Degradation	+4.76	-2.52

Melatonin Degradation	+4.2	-2.52
Serotonin Degradation	+4.2	-3.22
TCA Cycle II (Eukaryotic)	+2.632	-2.66

Table S2: List of IPA genes in GA-related GIM

1.	AES
2.	ANKRD6
3.	ARID1A
4.	ARR3
5.	ARRB1
6.	ARRB2
7.	AXIN1
8.	BCL9
9.	BTRC
10.	CDH1
11.	CDH10
12.	CDH11
13.	CDH12
14.	CDH13
15.	CDH15
16.	CDH16
17.	CDH17
18.	CDH18
19.	CDH19
20.	CDH2
21.	CDH20
22.	CDH22
23.	CDH23
24.	CDH24
25.	CDH3
26.	CDH4
27.	CDH5
28.	CDH6
29.	CDH7
30.	CDH8
31.	CDH9
32.	CDHR1
33.	CDHR2
34.	CELSR1
35.	CELSR2
36.	CELSR3
37.	CER1
38.	CHD1L
39.	CREBBP
40.	CSNK1A1

41.	CSNK1A1L
42.	CSNK1D
43.	CSNK1G2
44.	CSNK1G3
45.	CSNK2A1
46.	CSNK2A2
47.	CSNK2B
48.	CTBP1
49.	CTBP2
50.	CTNNA1
51.	CTNNA2
52.	CTNNA3
53.	CTNNAL1
54.	CTNNB1
55.	DACT1
56.	DCHS1
57.	DVL1
58.	DVL2
59.	DVL3
60.	EP300
61.	EP400
62.	FAT1
63.	FAT2
64.	FAT3
65.	FBXW11
66.	FRZB
67.	FZD1
68.	FZD10
69.	FZD2
70.	FZD3
71.	FZD4
72.	FZD5
73.	FZD6
74.	FZD7
75.	FZD8
76.	FZD9
77.	GNA11
78.	GNA14
79.	GNA15
80.	GNAQ
81.	GNB1
82.	GNB2
83.	GNB3
84.	GNB4
85.	GNG10
86.	GNG12

87.	GNG13
88.	GNG2
89.	GNG3
90.	GNG4
91.	GNG5
92.	GNG7
93.	GNG8
94.	GSK3B
95.	HDAC1
96.	HDAC2
97.	HDAC3
98.	HDAC8
99.	HELLS
100.	HLTF
101.	INO80
102.	ITPR1
103.	ITPR2
104.	ITPR3
105.	KREMEN1
106.	KREMEN2
107.	LEF1
108.	LRP5
109.	LRP6
110.	MAP3K7
111.	NFATC1
112.	NFATC2
113.	NFATC3
114.	NFATC4
115.	NKD1
116.	NKD2
117.	NLK
118.	PCDH1
119.	PCDH10
120.	PCDH11X
121.	PCDH11Y
122.	PCDH12
123.	PCDH15
124.	PCDH18
125.	PCDH19
126.	PCDH20
127.	PCDH7
128.	PCDH8
129.	PCDH9
130.	PCDHA1
131.	PCDHA10
132.	PCDHA11

133.	PCDHA12
134.	PCDHA13
135.	PCDHA2
136.	PCDHA3
137.	PCDHA4
138.	PCDHA5
139.	PCDHA6
140.	PCDHA7
141.	PCDHA8
142.	PCDHA9
143.	PCDHAC1
144.	PCDHAC2
145.	PCDHB1
146.	PCDHB10
147.	PCDHB11
148.	PCDHB12
149.	PCDHB13
150.	PCDHB14
151.	PCDHB15
152.	PCDHB16
153.	PCDHB2
154.	PCDHB3
155.	PCDHB4
156.	PCDHB5
157.	PCDHB6
158.	PCDHB7
159.	PCDHB8
160.	PCDHGA1
161.	PCDHGA10
162.	PCDHGA11
163.	PCDHGA12
164.	PCDHGA2
165.	PCDHGA3
166.	PCDHGA5
167.	PCDHGA6
168.	PCDHGA7
169.	PCDHGA8
170.	PCDHGB1
171.	PCDHGB2
172.	PCDHGB4
173.	PCDHGB6
174.	PCDHGB7
175.	PCDHGC3
176.	PCDHGC4
177.	PCDHGC5
178.	PLCB1

179.	PLCB2
180.	PLCB3
181.	PLCB4
182.	PPP2CA
183.	PPP2CB
184.	PPP2R5A
185.	PPP2R5B
186.	PPP2R5C
187.	PPP2R5D
188.	PPP2R5E
189.	PPP3CA
190.	PPP3CB
191.	PPP3CC
192.	PPP3R1
193.	PPP3R2
194.	PRKCA
195.	PRKCB
196.	PRKCD
197.	PRKCE
198.	PRKCG
199.	PRKCH
200.	PRKCI
201.	PRKCQ
202.	PRKCZ
203.	PYGO1
204.	PYGO2
205.	SAG
206.	SFRP1
207.	SFRP2
208.	SFRP4
209.	SFRP5
210.	SIAH1
211.	SMAD1
212.	SMAD4
213.	SMARCA1
214.	SMARCA2
215.	SMARCA4
216.	SMARCA5
217.	SMARCAL1
218.	SMARCB1
219.	SMARCC1
220.	SMARCC2
221.	SMARCD1
222.	SMARCD2
223.	SMARCD3
224.	SMARCE1

225.	SRCAP
226.	TBL1X
227.	TBL1XR1
228.	TBL1Y
229.	TCF7L1
230.	TCF7L2
231.	TLE1
232.	TLE2
233.	TLE3
234.	TLE4
235.	TLE6
236.	WNT1
237.	WNT10A
238.	WNT10B
239.	WNT11
240.	WNT16
241.	WNT2
242.	WNT2B
243.	WNT3
244.	WNT3A
245.	WNT4
246.	WNT5A
247.	WNT5B
248.	WNT6
249.	WNT7A
250.	WNT7B
251.	WNT8A
252.	WNT8B
253.	WNT9A
254.	WNT9B
255.	ATF2
256.	CDC42
257.	CREB1
258.	DDIT3
259.	ELK1
260.	H3F3A
261.	H3F3B
262.	HMGN1
263.	HRAS
264.	HSPB1
265.	MAP2K4
266.	MAP2K6
267.	MAP3K1
268.	MAP3K5
269.	MAP3K9
270.	MAPK14

271.	MAPKAPK2
272.	MAPKAPK5
273.	MAX
274.	MEF2A
275.	MEF2BNB-MEF2B
276.	MEF2C
277.	MEF2D
278.	MKNK1
279.	MYC
280.	NR2C2
281.	PLA2G1B
282.	RAC1
283.	RPS6KA5
284.	STAT1
285.	ATM
286.	ATR
287.	BRCA1
288.	BRCA2
289.	CHEK1
290.	CHEK2
291.	FANCA
292.	FANCC
293.	FANCD2
294.	FANCE
295.	FANCF
296.	FANCG
297.	HUS1
298.	MRE11A
299.	NBN
300.	RAD1
301.	RAD17
302.	RAD50
303.	RAD51
304.	RAD9A
305.	TP53
306.	TREX1
307.	AKR7A2
308.	CRYZ
309.	FOS
310.	FXYD2
311.	GSTA2
312.	HMOX1
313.	HMOX2
314.	JUN
315.	KEAP1
316.	MAFF

317.	MAFG
318.	MAFK
319.	NFE2L2
320.	POR
321.	UGT1A6
322.	CNDP2
323.	GCLC
324.	GCLM
325.	GGCT
326.	GGT5
327.	GSS
328.	OPLAH
329.	ADCY1
330.	ADRBK1
331.	CAMK2B
332.	CDK5
333.	CDK5R1
334.	DRD1
335.	DRD2
336.	GNAS
337.	GNGT1
338.	GRM1
339.	NCS1
340.	PPP1CA
341.	PPP1R1B
342.	PRKACB
343.	PRKACG
344.	ACTB
345.	ACTG1
346.	ACTR2
347.	ACTR3
348.	ADAM10
349.	ANKS1A
350.	AP2A1
351.	AP2A2
352.	AP2B1
353.	AP2M1
354.	AP2S1
355.	APH1A
356.	APH1B
357.	ARHGEF28
358.	ARHGEF7
359.	ARPC1A
360.	ARPC2
361.	ARPC3
362.	ARPC5

363.	CFL1
364.	CHN1
365.	CRK
366.	DNM1
367.	EFNA1
368.	EFNA2
369.	EFNA3
370.	EFNA4
371.	EFNA5
372.	EFNB1
373.	EFNB2
374.	EFNB3
375.	EPHA1
376.	EPHA10
377.	EPHA2
378.	EPHA3
379.	EPHA4
380.	EPHA5
381.	EPHA6
382.	EPHA7
383.	EPHA8
384.	EPHB1
385.	EPHB2
386.	EPHB3
387.	EPHB4
388.	EPHB6
389.	FYN
390.	GIT1
391.	GRIN1
392.	GRIN2B
393.	ITSN1
394.	KALRN
395.	LIMK1
396.	LYN
397.	MMP2
398.	MMP9
399.	MYH10
400.	MYH11
401.	MYH14
402.	MYH9
403.	MYL12A
404.	MYL12B
405.	MYL6
406.	MYL9
407.	NCK2
408.	NCSTN

409.	NGEF
410.	PAK1
411.	PAK3
412.	PSEN2
413.	PSENEN
414.	PTK2
415.	PTPN11
416.	RASA1
417.	RHOA
418.	ROCK1
419.	ROCK2
420.	SDC2
421.	SDCBP
422.	SIPA1L1
423.	SRC
424.	SS18
425.	TIAM1
426.	VAV2
427.	VAV3
428.	WASL
429.	YES1
430.	AKT1
431.	EIF3A
432.	EIF4A1
433.	EIF4B
434.	EIF4E
435.	EIF4EBP1
436.	EIF4G1
437.	FKBP1A
438.	GH1
439.	GHR
440.	IRS1
441.	MTOR
442.	PDK2
443.	PDPK1
444.	PIK3CA
445.	PIK3R1
446.	PTEN
447.	RHEB
448.	RPS6
449.	RPS6KB1
450.	TSC1
451.	TSC2
452.	ADIPOQ
453.	AGTR1
454.	APLN

455.	APLNR
456.	ATF4
457.	AVP
458.	BMPR2
459.	COL1A1
460.	CRP
461.	GHRL
462.	HIF1A
463.	IGF1
464.	LEP
465.	MIR224
466.	NAMPT
467.	OPRK1
468.	PPARG
469.	RETN
470.	AGR2
471.	AMOT
472.	AMOTL2
473.	AREG
474.	CTGF
475.	DICER1
476.	EGFR
477.	FRMD6
478.	GFER
479.	HMMR
480.	HPCA
481.	ILK
482.	LATS1
483.	LATS2
484.	MARK1
485.	MIR31
486.	MOB1A
487.	NEK2
488.	NEWENTRY
489.	NF2
490.	NPHP4
491.	PHLPP1
492.	PJA2
493.	PKP2
494.	PTPN14
495.	RASSF1
496.	RASSF6
497.	RB1
498.	SAV1
499.	STK3
500.	STK4

501.	TEAD1
502.	TEAD4
503.	TGFB1
504.	VPRBP
505.	WWTR1
506.	YAP1
507.	YY1AP1
508.	ABCF1
509.	ATF5
510.	BZW2
511.	EEF1E1
512.	EIF1
513.	EIF1AX
514.	EIF2A
515.	EIF2AK2
516.	EIF2B1
517.	EIF2S1
518.	EIF2S2
519.	EIF2S3
520.	EIF5
521.	MCL1
522.	VEGFA
523.	CCNH
524.	CDKN1A
525.	CUL4A
526.	CUL4B
527.	DDB1
528.	DDB2
529.	E2F1
530.	ERCC1
531.	ERCC2
532.	ERCC3
533.	ERCC4
534.	ERCC5
535.	GLI1
536.	GTF2H4
537.	H2AFX
538.	HMGB1
539.	MDM2
540.	MIR192
541.	MMS19
542.	PARP1
543.	RNF2
544.	RPA1
545.	SIRT1
546.	TP63

547.	USP7
548.	VCP
549.	XPA
550.	XPC
551.	XRCC1
552.	CDKN1B
553.	CORO1B
554.	CSPG5
555.	ERBB2
556.	ERBB3
557.	ERBB4
558.	L1CAM
559.	MIR145
560.	MUC4
561.	NRG1
562.	NRG3
563.	NRG4
564.	P2RY2
565.	PRKD1
566.	RNF41
567.	SLC1A6
568.	TNF
569.	TRPV1
570.	YY1
571.	ABI1
572.	ABL1
573.	ADGRA2
574.	AIRE
575.	AJUBA
576.	ARF6
577.	ARHGAP24
578.	ARHGAP32
579.	AXL
580.	BAIAP2
581.	BAIAP2L1
582.	BCAR1
583.	BCAR3
584.	BCL2
585.	BCR
586.	CADM1
587.	CASP3
588.	CCL2
589.	CCL5
590.	CCND1
591.	CCR1
592.	CCR5

593.	CEP135
594.	CHN2
595.	CHUK
596.	CTNND1
597.	CTTN
598.	CXCL12
599.	CXCR4
600.	DOCK1
601.	DOCK2
602.	ELMO1
603.	ELMO2
604.	ENOX2
605.	F2
606.	FER
607.	FMNL1
608.	FN1
609.	FSCN1
610.	GRASP
611.	HRH1
612.	IL10
613.	IL1B
614.	ITGA2B
615.	ITGA4
616.	ITGB1
617.	ITGB3
618.	KIT
619.	LCK
620.	LPAR1
621.	MAPK8
622.	MIR149
623.	MIR21
624.	MTSS1
625.	NCF2
626.	NCKIPSD
627.	NFKB1
628.	NISCH
629.	NOX1
630.	NOX5
631.	NOXA1
632.	OCRL
633.	PARVB
634.	PDGFB
635.	PIK3CG
636.	PIP5K1A
637.	PIP5K1C
638.	PLAU

639.	PLAUR
640.	PLCG2
641.	F2RL1
642.	KITLG
643.	MIF
644.	NME1
645.	TXN
646.	ADAM17
647.	ANGPT2
648.	ANKS1B
649.	APC
650.	AR
651.	ARHGEF4
652.	BIRC5
653.	BMP7
654.	BMPR1B
655.	C3
656.	CD40LG
657.	CD44
658.	CDKN2A
659.	CFTR
660.	CLIP1
661.	CNKSR3
662.	COL3A1
663.	CSF1
664.	CXCL8
665.	CYP19A1
666.	DLL4
667.	EGF
668.	EGR1
669.	EREG
670.	ETS1
671.	EZR
672.	FAS
673.	FGF2
674.	FGF7
675.	FLT3LG
676.	FPR2
677.	FURIN
678.	GAB1
679.	GINGF3
680.	GRP
681.	HEY2
682.	HGF
683.	HGFAC
684.	HLX

685.	HPN
686.	HPSE
687.	ID1
688.	IGF1R
689.	IL11
690.	IL1RN
691.	IL32
692.	IL6
693.	IPCEF1
694.	ITGA2
695.	ITGB2
696.	ITGB4
697.	JUNB
698.	KIF5B
699.	KLF4
700.	KLF6
701.	KRAS
702.	LIF
703.	LUM
704.	MACC1
705.	MAPK1
706.	MET
707.	MIR199A1
708.	MIR200C
709.	MIR27B
710.	MIR519C
711.	MMP1
712.	MMP3
713.	MMP7
714.	MMP8
715.	MYD88
716.	NFYC
717.	NOTCH1
718.	NOTCH3
719.	NOX4
720.	PAK2
721.	PAK6
722.	PDGFA
723.	PGF
724.	PLG
725.	PROM1
726.	PTGS2
727.	PXN
728.	RELA
729.	RGMB
730.	S1PR1

731.	S1PR2
732.	S1PR3
733.	SDC1
734.	SERPINE1
735.	SHC1
736.	SKIL
737.	SLC2A4
738.	SMAD2
739.	SMYD3
740.	SNAI1
741.	SP1
742.	SPINT1
743.	SPINT2
744.	SPP1
745.	ST14
746.	STAT3
747.	TGIF1
748.	THBS1
749.	TICAM2
750.	TIMP1
751.	TLR4
752.	WASF2
753.	XIAP
754.	AFP
755.	ALDH1A1
756.	CALB2
757.	CD24
758.	CLDN7
759.	EPCAM
760.	ERAP2
761.	FOLR1
762.	GPA33
763.	GPNMB
764.	IFNAR2
765.	KRT19
766.	MAML1
767.	MLH1
768.	MSH2
769.	PTTG1
770.	TSPAN8
771.	VCAM1
772.	WISP1
773.	ABCA1
774.	SREBF1
775.	ADPGK
776.	AGL

777.	B4GALT1
778.	B4GALT2
779.	G6PC
780.	G6PC2
781.	G6PC3
782.	GAA
783.	GANAB
784.	GANC
785.	GBA
786.	GBA2
787.	GBA3
788.	GCK
789.	GCKR
790.	GLA
791.	GPI
792.	HK1
793.	HK2
794.	HK3
795.	HKDC1
796.	LALBA
797.	LCT
798.	MGAM
799.	MOGS
800.	PGM1
801.	SI
802.	SLC2A1
803.	TREH
804.	HSD17B1
805.	ADH1B
806.	ADH6
807.	ALDH2
808.	ALDH3A2
809.	MAOA
810.	SULT1A1
811.	SULT1A2
812.	UGT2B11
813.	ABCC2
814.	CHST5
815.	CYP3A4
816.	PPP1R14D
817.	SULT1A3/SULT1A4
818.	UGT1A1
819.	UGT2B7
820.	UGT2B17
821.	CLDN3
822.	HLA-DPB1

823.	HLA-DQB1
824.	HLA-DPA1
825.	HLA-DMB
826.	RPS13
827.	RPL22
828.	RPL13A
829.	RPL11
830.	RPL38
831.	RPS7
832.	RPL6
833.	RPL26
834.	RPL5
835.	RPS3
836.	PCBD1
837.	RPL23
838.	RPL10A
839.	KLF5
840.	RPL14
841.	RPL1
842.	RPS20
843.	EEF1A1
844.	RPS23
845.	RPL15
846.	FGFR4
847.	RPL35A
848.	RPL9
849.	RPS15A
850.	RPSA
851.	SOCS3
852.	RPS25
853.	RPL37
854.	RPL30
855.	APP
856.	RPLP1
857.	RPS5
858.	RPS24
859.	RPL13
860.	IGFBP4
861.	CYSTM1
862.	DDC
863.	RPS14
864.	WFDC2
865.	RBM47

Table S3 List of % genes per term in GA-related GIM

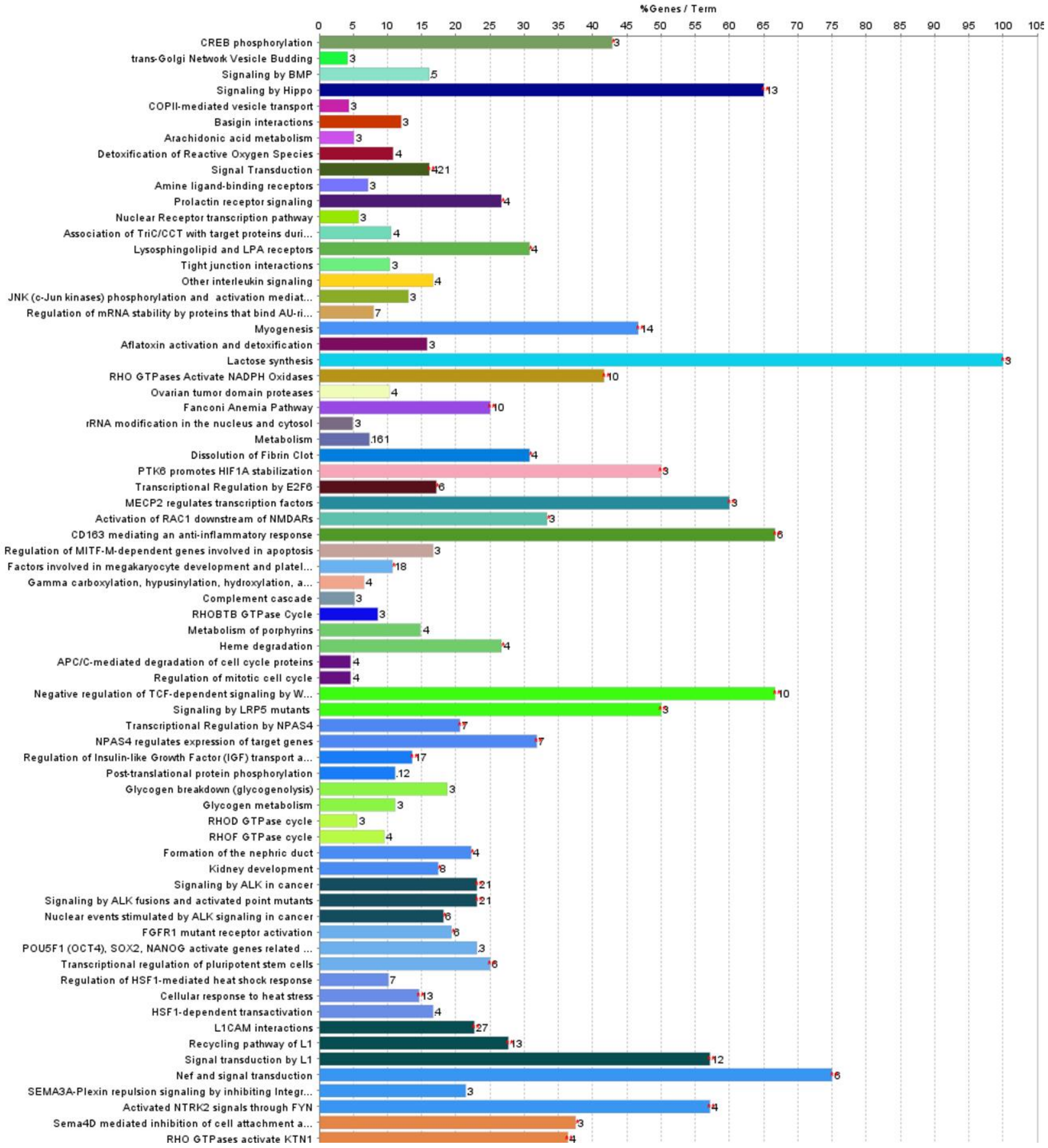

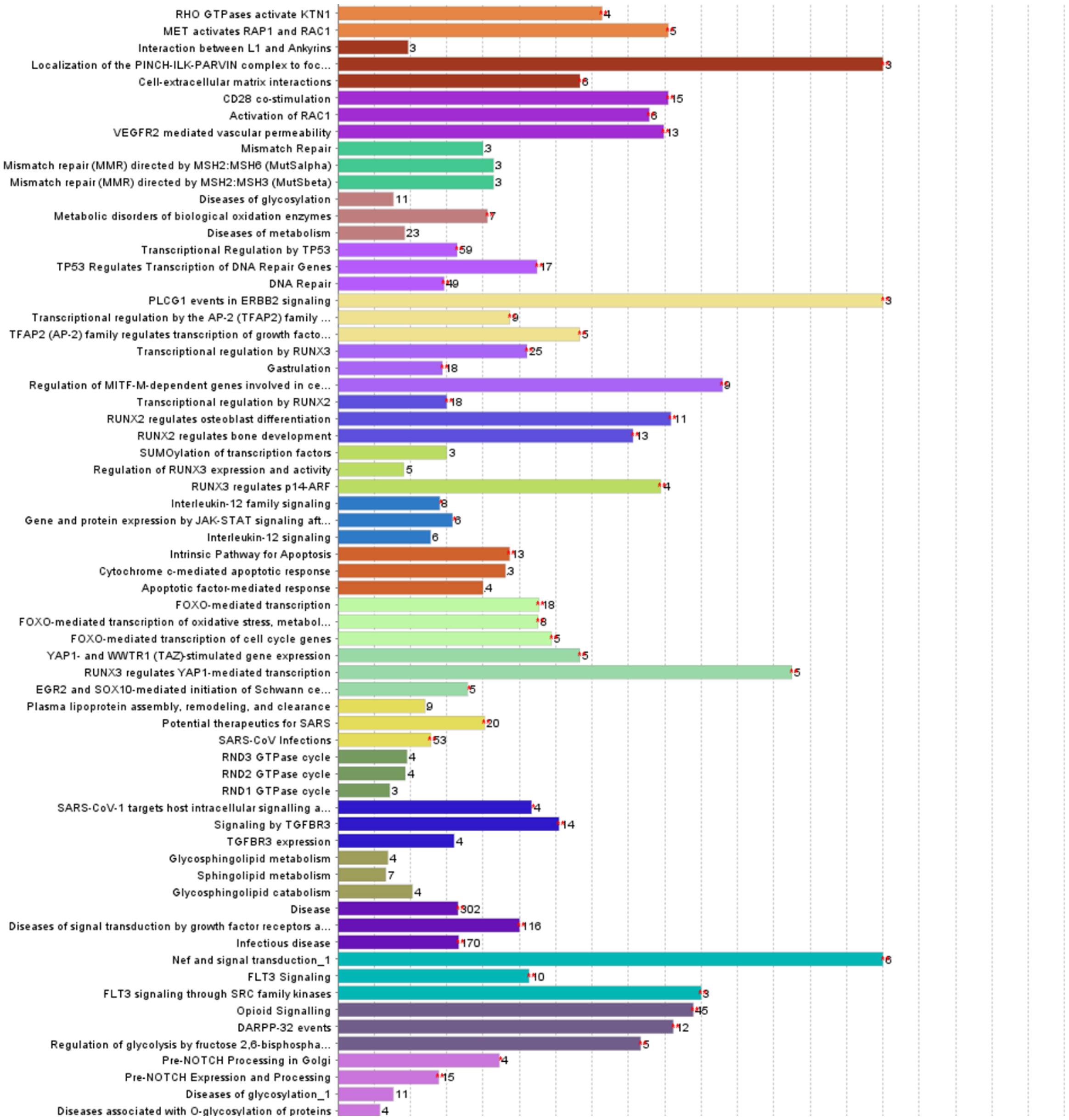
RHO GTPases activate KTN1
MET activates RAP1 and RAC1
Interaction between L1 and Ankyrins
Localization of the PINCH-ILK-PARVIN complex to foc...
Cell-extracellular matrix interactions
CD28 co-stimulation
Activation of RAC1
VEGFR2 mediated vascular permeability
Mismatch Repair
Mismatch repair (MMR) directed by MSH2:MSH6 (MutSalpha)
Mismatch repair (MMR) directed by MSH2:MSH3 (MutSbeta)
Diseases of glycosylation
Metabolic disorders of biological oxidation enzymes
Diseases of metabolism
Transcriptional Regulation by TP53
TP53 Regulates Transcription of DNA Repair Genes
DNA Repair
PLCG1 events in ERBB2 signaling
Transcriptional regulation by the AP-2 (TFAP2) family ...
TFAP2 (AP-2) family regulates transcription of growth facto...
Transcriptional regulation by RUNX3
Gastrulation
Regulation of MITF-M-dependent genes involved in ce...
Transcriptional regulation by RUNX2
RUNX2 regulates osteoblast differentiation
RUNX2 regulates bone development
SUMOylation of transcription factors
Regulation of RUNX3 expression and activity
RUNX3 regulates p14-ARF
Interleukin-12 family signaling
Gene and protein expression by JAK-STAT signaling aft...
Interleukin-12 signaling
Intrinsic Pathway for Apoptosis
Cytochrome c-mediated apoptotic response
Apoptotic factor-mediated response
FOXO-mediated transcription
FOXO-mediated transcription of oxidative stress, metabol...
FOXO-mediated transcription of cell cycle genes
YAP1- and WWTR1 (TAZ)-stimulated gene expression
RUNX3 regulates YAP1-mediated transcription
EGR2 and SOX10-mediated initiation of Schwann ce...
Plasma lipoprotein assembly, remodeling, and clearance
Potential therapeutics for SARS
SARS-CoV Infections
RND3 GTPase cycle
RND2 GTPase cycle
RND1 GTPase cycle
SARS-CoV-1 targets host intracellular signalling a...
Signaling by TGFBR3
TGFBR3 expression
Glycosphingolipid metabolism
Sphingolipid metabolism
Glycosphingolipid catabolism
Disease
Diseases of signal transduction by growth factor receptors a...
Infectious disease
Nef and signal transduction_1
FLT3 Signaling
FLT3 signaling through SRC family kinases
Opioid Signalling
DARPP-32 events
Regulation of glycolysis by fructose 2,6-bisphospha...
Pre-NOTCH Processing in Golgi
Pre-NOTCH Expression and Processing
Diseases of glycosylation_1
Diseases associated with O-glycosylation of proteins
4
5
3
3
6
15
6
13
3
3
3
11
7
23
59
17
49
3
9
5
25
18
9
18
11
13
3
5
4
8
6
6
13
3
4
18
8
5
5
5
5
9
20
53
4
4
3
4
14
4
4
7
4
302
116
170
6
10
3
45
12
5
4
15
11
4

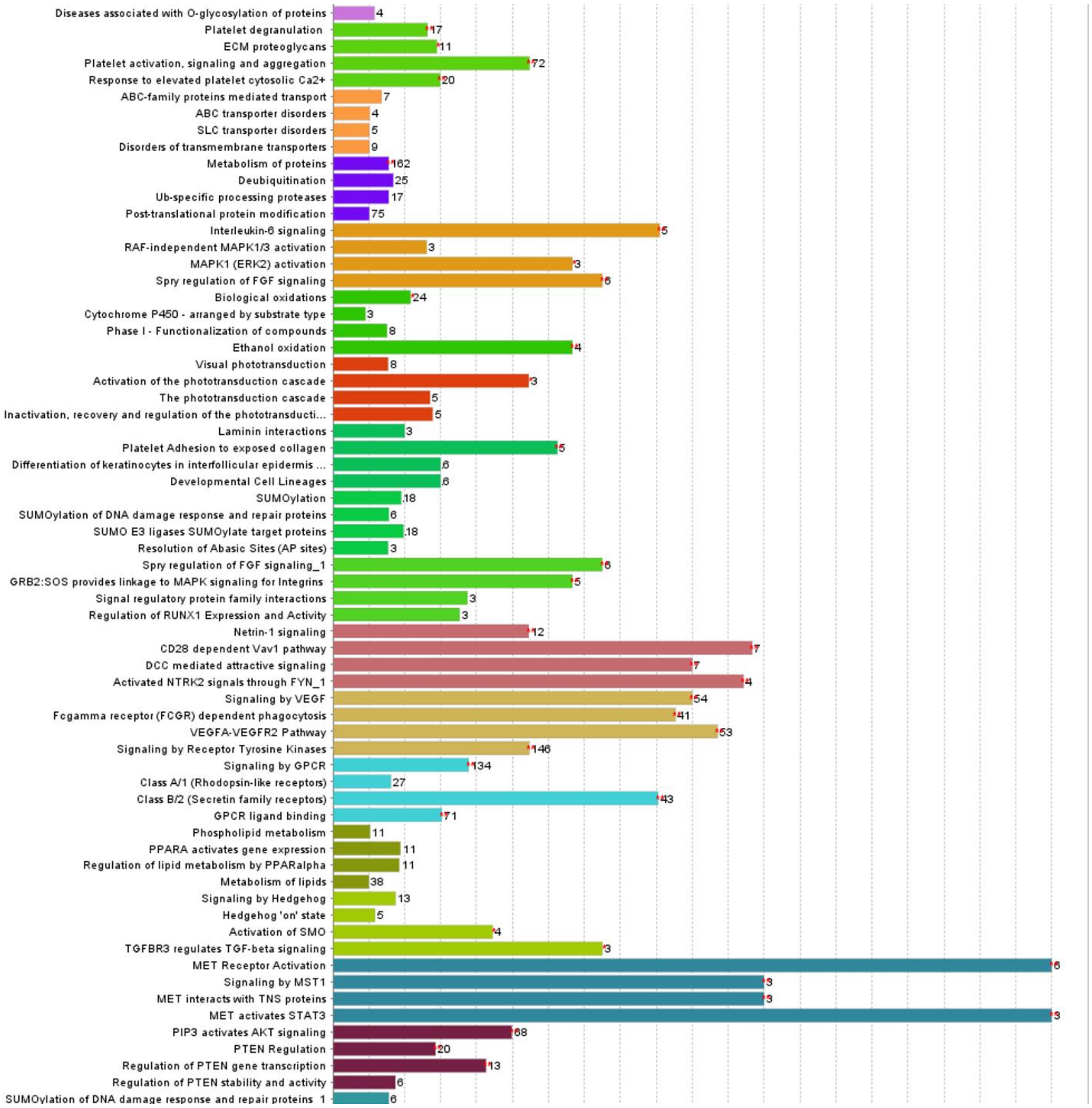
Diseases associated with O-glycosylation of proteins 4
Platelet degranulation 17
ECM proteoglycans 11
Platelet activation, signaling and aggregation 72
Response to elevated platelet cytosolic Ca2+ 20
ABC-family proteins mediated transport 7
ABC transporter disorders 4
SLC transporter disorders 5
Disorders of transmembrane transporters 9
Metabolism of proteins 162
Deubiquitination 25
Ub-specific processing proteases 17
Post-translational protein modification 75
Interleukin-6 signaling 5
RAF-independent MAPK1/3 activation 3
MAPK1 (ERK2) activation 3
Spry regulation of FGF signaling 6
Biological oxidations 24
Cytochrome P450 - arranged by substrate type 3
Phase I - Functionalization of compounds 8
Ethanol oxidation 4
Visual phototransduction 8
Activation of the phototransduction cascade 3
The phototransduction cascade 5
Inactivation, recovery and regulation of the phototransducti... 5
Laminin interactions 3
Platelet Adhesion to exposed collagen 5
Differentiation of keratinocytes in interfollicular epidermis ... 6
Developmental Cell Lineages 6
SUMOylation 18
SUMOylation of DNA damage response and repair proteins 6
SUMO E3 ligases SUMOylate target proteins 18
Resolution of Abasic Sites (AP sites) 3
Spry regulation of FGF signaling_1 6
GRB2:SOS provides linkage to MAPK signaling for Integrins 5
Signal regulatory protein family interactions 3
Regulation of RUNX1 Expression and Activity 3
Netrin-1 signaling 12
CD28 dependent Vav1 pathway 7
DCC mediated attractive signaling 7
Activated NTRK2 signals through FYN_1 4
Signaling by VEGF 54
Fcgamma receptor (FCGR) dependent phagocytosis 41
VEGFA-VEGFR2 Pathway 53
Signaling by Receptor Tyrosine Kinases 146
Signaling by GPCR 134
Class A/1 (Rhodopsin-like receptors) 27
Class B/2 (Secretin family receptors) 43
GPCR ligand binding 71
Phospholipid metabolism 11
PPARA activates gene expression 11
Regulation of lipid metabolism by PPARalpha 11
Metabolism of lipids 38
Signaling by Hedgehog 13
Hedgehog 'on' state 5
Activation of SMO 4
TGFBR3 regulates TGF-beta signaling 3
MET Receptor Activation 6
Signaling by MST1 3
MET interacts with TNS proteins 3
MET activates STAT3 3
PIP3 activates AKT signaling 68
PTEN Regulation 20
Regulation of PTEN gene transcription 13
Regulation of PTEN stability and activity 6
SUMOylation of DNA damage response and repair proteins_1 6

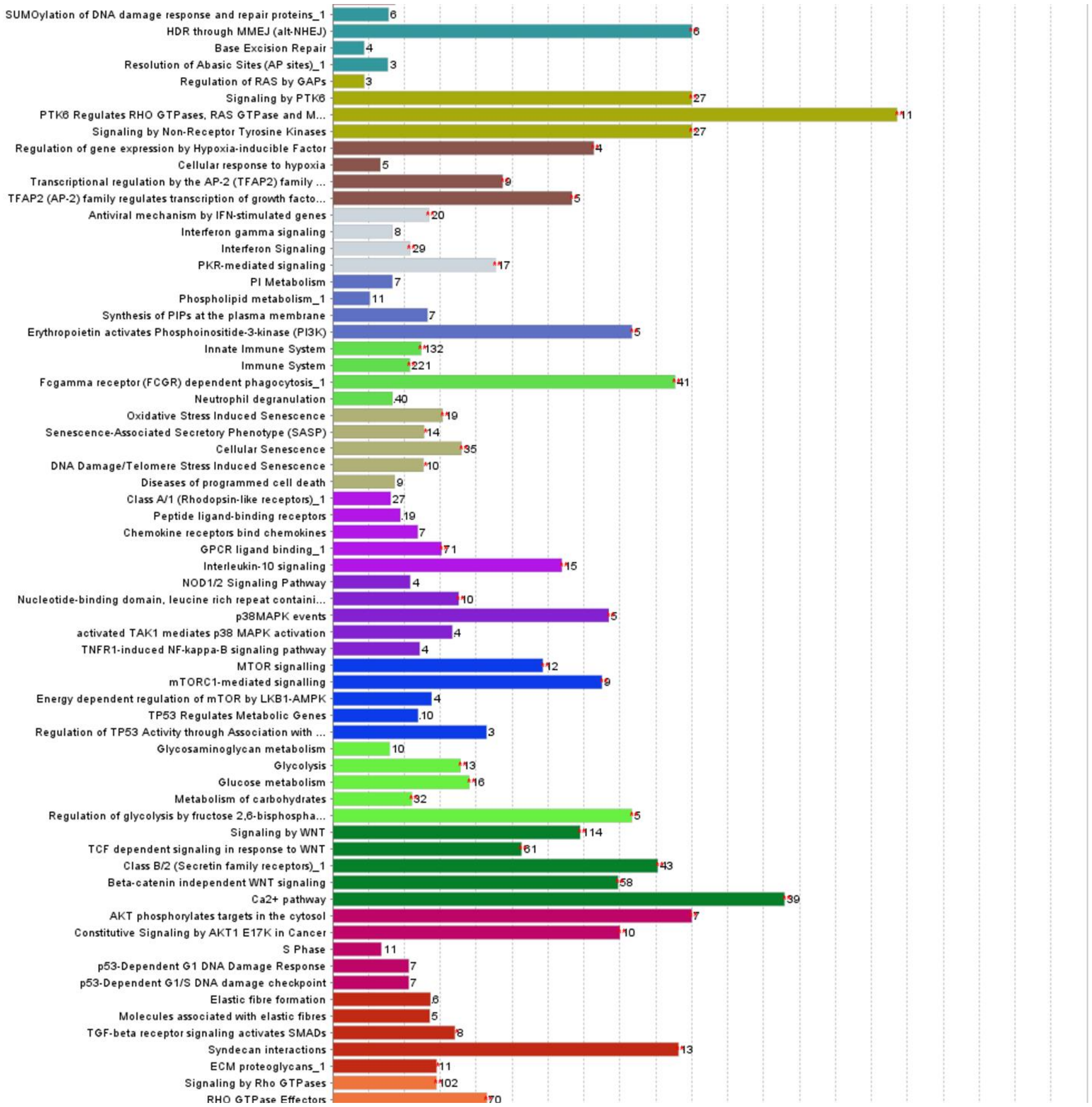
SUMOylation of DNA damage response and repair proteins_1
6
HDR through MMEJ (alt-NHEJ)
6
Base Excision Repair
4
Resolution of Abasic Sites (AP sites)_1
3
Regulation of RAS by GAPs
3
Signaling by PTK6
27
PTK6 Regulates RHO GTPases, RAS GTPase and M...
11
Signaling by Non-Receptor Tyrosine Kinases
27
Regulation of gene expression by Hypoxia-inducible Factor
4
Cellular response to hypoxia
5
Transcriptional regulation by the AP-2 (TFAP2) family ...
9
TFAP2 (AP-2) family regulates transcription of growth facto...
5
Antiviral mechanism by IFN-stimulated genes
20
Interferon gamma signaling
8
Interferon Signaling
29
PKR-mediated signaling
17
PI Metabolism
7
Phospholipid metabolism_1
11
Synthesis of PIPs at the plasma membrane
7
Erythropoietin activates Phosphoinositide-3-kinase (PI3K)
5
Innate Immune System
132
Immune System
221
Fcgamma receptor (FCGR) dependent phagocytosis_1
41
Neutrophil degranulation
40
Oxidative Stress Induced Senescence
19
Senescence-Associated Secretory Phenotype (SASP)
14
Cellular Senescence
35
DNA Damage/Telomere Stress Induced Senescence
10
Diseases of programmed cell death
9
Class A/1 (Rhodopsin-like receptors)_1
27
Peptide ligand-binding receptors
19
Chemokine receptors bind chemokines
7
GPCR ligand binding_1
71
Interleukin-10 signaling
15
NOD1/2 Signaling Pathway
4
Nucleotide-binding domain, leucine rich repeat containi...
10
p38MAPK events
5
activated TAK1 mediates p38 MAPK activation
4
TNFR1-induced NF-kappa-B signaling pathway
4
MTOR signalling
12
mTORC1-mediated signalling
9
Energy dependent regulation of mTOR by LKB1-AMPK
4
TP53 Regulates Metabolic Genes
10
Regulation of TP53 Activity through Association with ...
3
Glycosaminoglycan metabolism
10
Glycolysis
13
Glucose metabolism
16
Metabolism of carbohydrates
32
Regulation of glycolysis by fructose 2,6-bisphospha...
5
Signaling by WNT
114
TCF dependent signaling in response to WNT
61
Class B/2 (Secretin family receptors)_1
43
Beta-catenin independent WNT signaling
58
Ca2+ pathway
39
AKT phosphorylates targets in the cytosol
7
Constitutive Signaling by AKT1 E17K in Cancer
10
S Phase
11
p53-Dependent G1 DNA Damage Response
7
p53-Dependent G1/S DNA damage checkpoint
7
Elastic fibre formation
6
Molecules associated with elastic fibres
5
TGF-beta receptor signaling activates SMADs
8
Syndecan interactions
13
ECM proteoglycans_1
11
Signaling by Rho GTPases
102
RHO GTPase Effectors
70

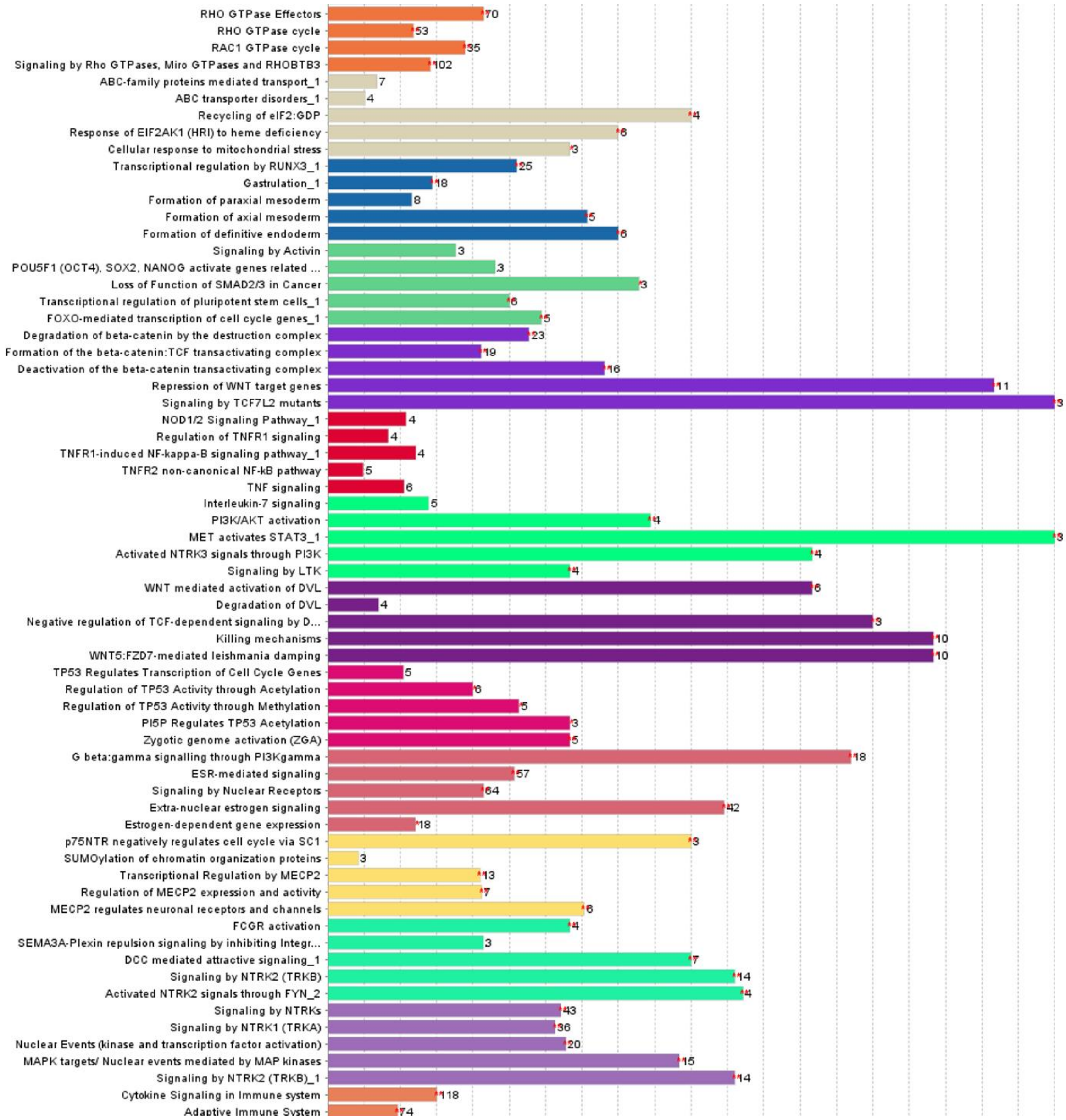
RHO GTPase Effectors
RHO GTPase cycle
RAC1 GTPase cycle
Signaling by Rho GTPases, Miro GTPases and RHOBTB3
ABC-family proteins mediated transport_1
ABC transporter disorders_1
Recycling of eIF2:GDP
Response of EIF2AK1 (HRI) to heme deficiency
Cellular response to mitochondrial stress
Transcriptional regulation by RUNX3_1
Gastrulation_1
Formation of paraxial mesoderm
Formation of axial mesoderm
Formation of definitive endoderm
Signaling by Activin
POU5F1 (OCT4), SOX2, NANOG activate genes related ...
Loss of Function of SMAD2/3 in Cancer
Transcriptional regulation of pluripotent stem cells_1
FOXO-mediated transcription of cell cycle genes_1
Degradation of beta-catenin by the destruction complex
Formation of the beta-catenin:TCF transactivating complex
Deactivation of the beta-catenin transactivating complex
Repression of WNT target genes
Signaling by TCF7L2 mutants
NOD1/2 Signaling Pathway_1
Regulation of TNFR1 signaling
TNFR1-induced NF-kappa-B signaling pathway_1
TNFR2 non-canonical NF-kB pathway
TNF signaling
Interleukin-7 signaling
PI3K/AKT activation
MET activates STAT3_1
Activated NTRK3 signals through PI3K
Signaling by LTK
WNT mediated activation of DVL
Degradation of DVL
Negative regulation of TCF-dependent signaling by D...
Killing mechanisms
WNT5:FZD7-mediated leishmania damping
TP53 Regulates Transcription of Cell Cycle Genes
Regulation of TP53 Activity through Acetylation
Regulation of TP53 Activity through Methylation
PI5P Regulates TP53 Acetylation
Zygotic genome activation (ZGA)
G beta:gamma signalling through PI3Kgamma
ESR-mediated signaling
Signaling by Nuclear Receptors
Extra-nuclear estrogen signaling
Estrogen-dependent gene expression
p75NTR negatively regulates cell cycle via SC1
SUMOylation of chromatin organization proteins
Transcriptional Regulation by MECP2
Regulation of MECP2 expression and activity
MECP2 regulates neuronal receptors and channels
FCGR activation
SEMA3A-Plexin repulsion signaling by inhibiting Integr...
DCC mediated attractive signaling_1
Signaling by NTRK2 (TRKB)
Activated NTRK2 signals through FYN_2
Signaling by NTRKs
Signaling by NTRK1 (TRKA)
Nuclear Events (kinase and transcription factor activation)
MAPK targets/ Nuclear events mediated by MAP kinases
Signaling by NTRK2 (TRKB)_1
Cytokine Signaling in Immune system
Adaptive Immune System
70
53
35
102
7
4
4
6
3
25
18
8
5
6
3
3
3
6
5
23
19
16
11
3
4
4
4
5
6
5
4
3
4
4
6
4
3
10
10
5
6
5
3
5
18
57
64
42
18
3
3
13
7
6
4
3
7
14
4
43
36
20
15
14
118
74

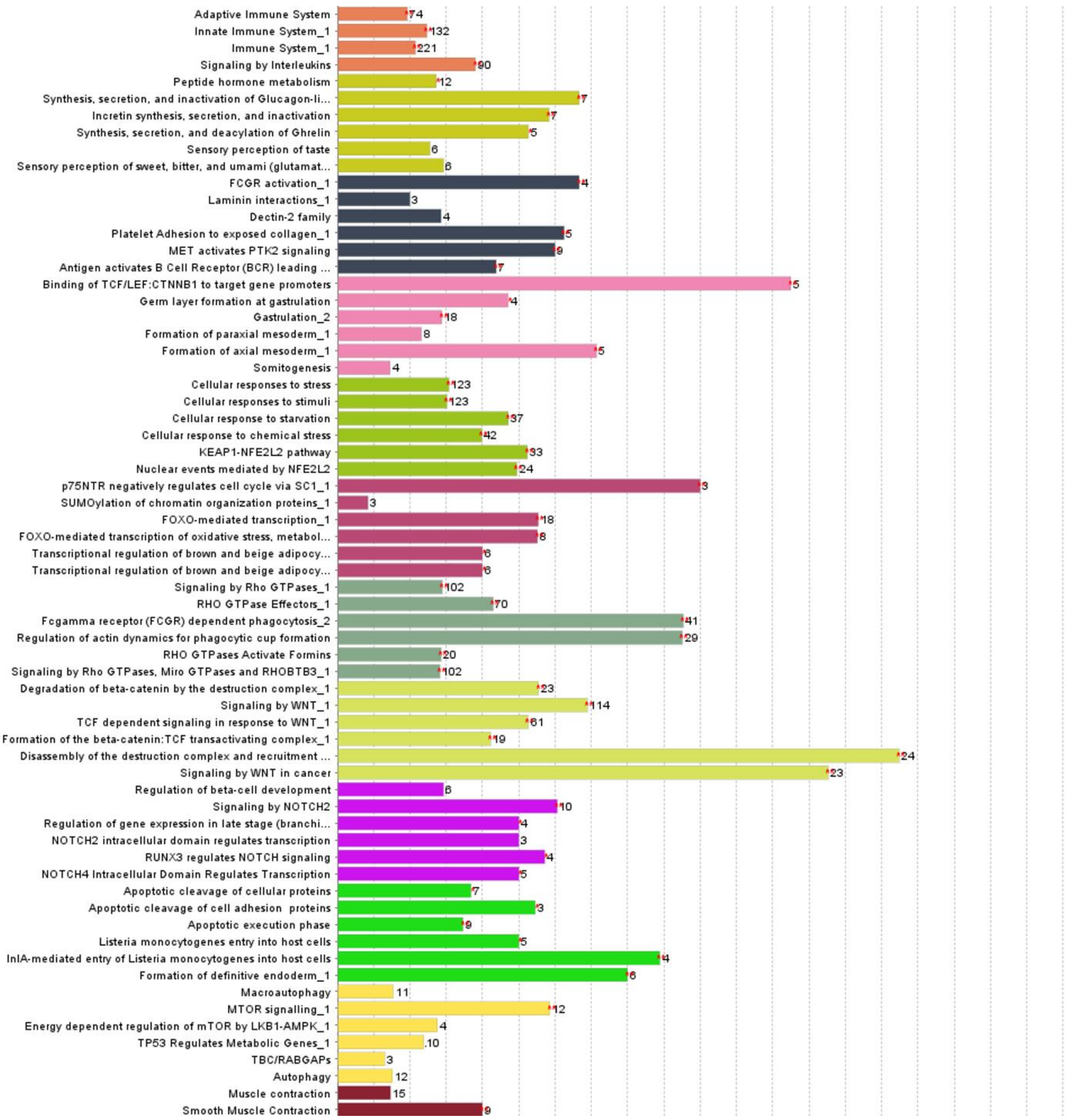
Adaptive Immune System 74
Innate Immune System_1 132
Immune System_1 221
Signaling by Interleukins 90
Peptide hormone metabolism 12
Synthesis, secretion, and inactivation of Glucagon-li... 7
Incretin synthesis, secretion, and inactivation 7
Synthesis, secretion, and deacylation of Ghrelin 5
Sensory perception of taste 6
Sensory perception of sweet, bitter, and umami (glutamat... 6
FCGR activation_1 4
Laminin interactions_1 3
Dectin-2 family 4
Platelet Adhesion to exposed collagen_1 5
MET activates PTK2 signaling 9
Antigen activates B Cell Receptor (BCR) leading ... 7
Binding of TCF/LEF:CTNNB1 to target gene promoters 5
Germ layer formation at gastrulation 4
Gastrulation_2 18
Formation of paraxial mesoderm_1 8
Formation of axial mesoderm_1 5
Somitogenesis 4
Cellular responses to stress 123
Cellular responses to stimuli 123
Cellular response to starvation 37
Cellular response to chemical stress 42
KEAP1-NFE2L2 pathway 33
Nuclear events mediated by NFE2L2 24
p75NTR negatively regulates cell cycle via SC1_1 3
SUMOylation of chromatin organization proteins_1 3
FOXO-mediated transcription_1 18
FOXO-mediated transcription of oxidative stress, metabol... 8
Transcriptional regulation of brown and beige adipocy... 6
Transcriptional regulation of brown and beige adipocy... 6
Signaling by Rho GTPases_1 102
RHO GTPase Effectors_1 70
Fcgamma receptor (FCGR) dependent phagocytosis_2 41
Regulation of actin dynamics for phagocytic cup formation 29
RHO GTPases Activate Formins 20
Signaling by Rho GTPases, Miro GTPases and RHOBTB3_1 102
Degradation of beta-catenin by the destruction complex_1 23
Signaling by WNT_1 114
TCF dependent signaling in response to WNT_1 61
Formation of the beta-catenin:TCF transactivating complex_1 19
Disassembly of the destruction complex and recruitment ... 24
Signaling by WNT in cancer 23
Regulation of beta-cell development 6
Signaling by NOTCH2 10
Regulation of gene expression in late stage (branchi... 4
NOTCH2 intracellular domain regulates transcription 3
RUNX3 regulates NOTCH signaling 4
NOTCH4 Intracellular Domain Regulates Transcription 5
Apoptotic cleavage of cellular proteins 7
Apoptotic cleavage of cell adhesion proteins 3
Apoptotic execution phase 9
Listeria monocytogenes entry into host cells 5
InlA-mediated entry of Listeria monocytogenes into host cells 4
Formation of definitive endoderm_1 6
Macroautophagy 11
MTOR signalling_1 12
Energy dependent regulation of mTOR by LKB1-AMPK_1 4
TP53 Regulates Metabolic Genes_1 10
TBC/RABGAPs 3
Autophagy 12
Muscle contraction 15
Smooth Muscle Contraction 9

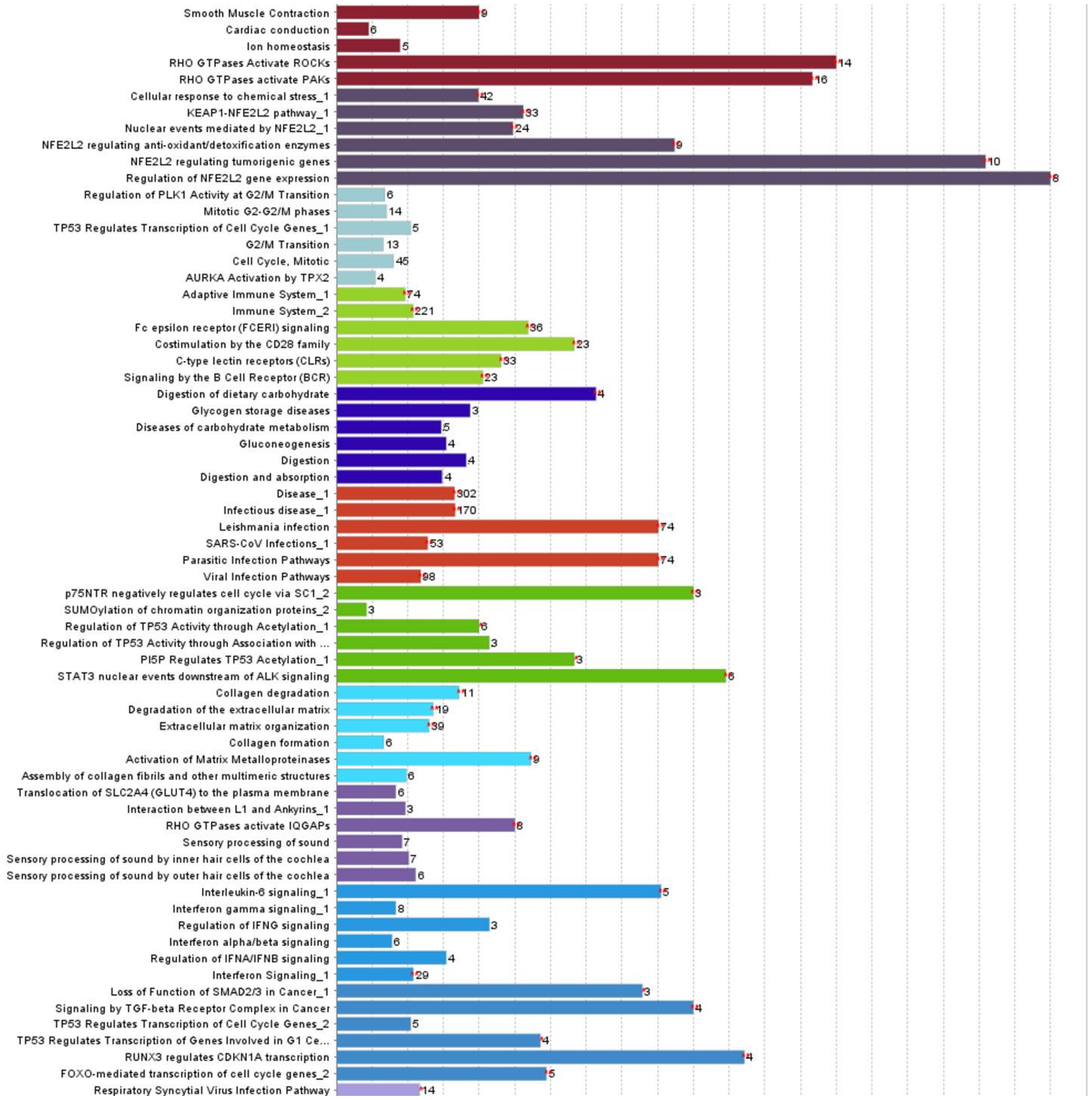
Smooth Muscle Contraction 9
Cardiac conduction 6
Ion homeostasis 5
RHO GTPases Activate ROCKs 14
RHO GTPases activate PAKs 16
Cellular response to chemical stress_1 42
KEAP1-NFE2L2 pathway_1 33
Nuclear events mediated by NFE2L2_1 24
NFE2L2 regulating anti-oxidant/detoxification enzymes 9
NFE2L2 regulating tumorigenic genes 10
Regulation of NFE2L2 gene expression 8
Regulation of PLK1 Activity at G2/M Transition 6
Mitotic G2-G2/M phases 14
TP53 Regulates Transcription of Cell Cycle Genes_1 5
G2/M Transition 13
Cell Cycle, Mitotic 45
AURKA Activation by TPX2 4
Adaptive Immune System_1 74
Immune System_2 221
Fc epsilon receptor (FCERI) signaling 36
Costimulation by the CD28 family 23
C-type lectin receptors (CLRs) 33
Signaling by the B Cell Receptor (BCR) 23
Digestion of dietary carbohydrate 4
Glycogen storage diseases 3
Diseases of carbohydrate metabolism 5
Gluconeogenesis 4
Digestion 4
Digestion and absorption 4
Disease_1 302
Infectious disease_1 170
Leishmania infection 74
SARS-CoV Infections_1 53
Parasitic Infection Pathways 74
Viral Infection Pathways 98
p75NTR negatively regulates cell cycle via SC1_2 3
SUMOylation of chromatin organization proteins_2 3
Regulation of TP53 Activity through Acetylation_1 6
Regulation of TP53 Activity through Association with ... 3
PI5P Regulates TP53 Acetylation_1 3
STAT3 nuclear events downstream of ALK signaling 6
Collagen degradation 11
Degradation of the extracellular matrix 19
Extracellular matrix organization 39
Collagen formation 6
Activation of Matrix Metalloproteinases 9
Assembly of collagen fibrils and other multimeric structures 6
Translocation of SLC2A4 (GLUT4) to the plasma membrane 6
Interaction between L1 and Ankyrins_1 3
RHO GTPases activate IQGAPs 8
Sensory processing of sound 7
Sensory processing of sound by inner hair cells of the cochlea 7
Sensory processing of sound by outer hair cells of the cochlea 6
Interleukin-6 signaling_1 5
Interferon gamma signaling_1 8
Regulation of IFNG signaling 3
Interferon alpha/beta signaling 6
Regulation of IFNA/IFNB signaling 4
Interferon Signaling_1 29
Loss of Function of SMAD2/3 in Cancer_1 3
Signaling by TGF-beta Receptor Complex in Cancer 4
TP53 Regulates Transcription of Cell Cycle Genes_2 5
TP53 Regulates Transcription of Genes Involved in G1 Ce... 4
RUNX3 regulates CDKN1A transcription 4
FOXO-mediated transcription of cell cycle genes_2 5
Respiratory Syncytial Virus Infection Pathway 14

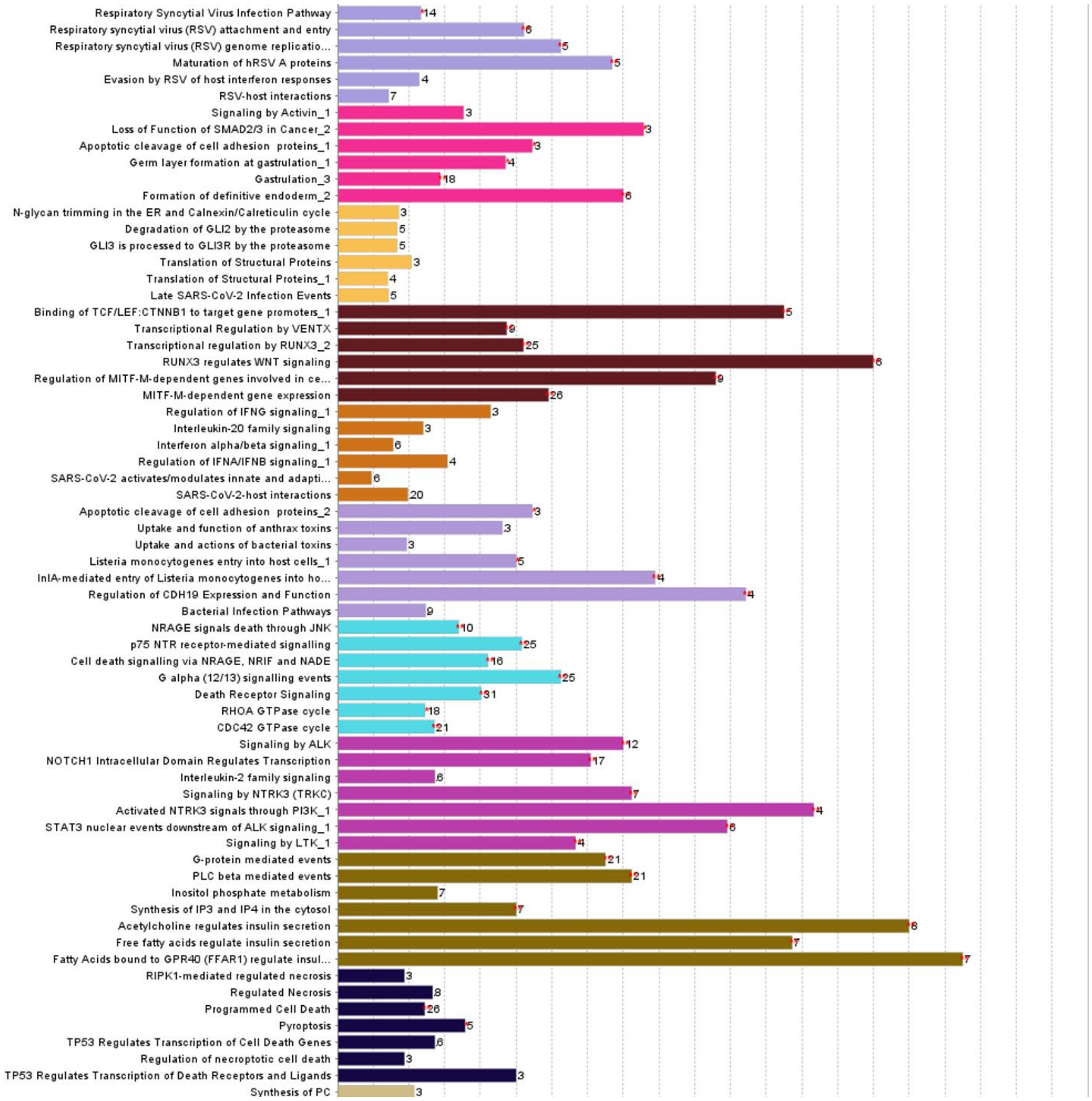
Respiratory Syncytial Virus Infection Pathway 14
Respiratory syncytial virus (RSV) attachment and entry 6
Respiratory syncytial virus (RSV) genome replicatio... 5
Maturation of hRSV A proteins 5
Evasion by RSV of host interferon responses 4
RSV-host interactions 7
Signaling by Activin_1 3
Loss of Function of SMAD2/3 in Cancer_2 3
Apoptotic cleavage of cell adhesion proteins_1 3
Germ layer formation at gastrulation_1 4
Gastrulation_3 18
Formation of definitive endoderm_2 6
N-glycan trimming in the ER and Calnexin/Calreticulin cycle 3
Degradation of GLI2 by the proteasome 5
GLI3 is processed to GLI3R by the proteasome 5
Translation of Structural Proteins 3
Translation of Structural Proteins_1 4
Late SARS-CoV-2 Infection Events 5
Binding of TCF/LEF:CTNNB1 to target gene promoters_1 5
Transcriptional Regulation by VENTX 9
Transcriptional regulation by RUNX3_2 25
RUNX3 regulates WNT signaling 6
Regulation of MITF-M-dependent genes involved in ce... 9
MITF-M-dependent gene expression 26
Regulation of IFNG signaling_1 3
Interleukin-20 family signaling 3
Interferon alpha/beta signaling_1 6
Regulation of IFNA/IFNB signaling_1 4
SARS-CoV-2 activates/modulates innate and adapti... 6
SARS-CoV-2-host interactions 20
Apoptotic cleavage of cell adhesion proteins_2 3
Uptake and function of anthrax toxins 3
Uptake and actions of bacterial toxins 3
Listeria monocytogenes entry into host cells_1 5
InlA-mediated entry of Listeria monocytogenes into ho... 4
Regulation of CDH19 Expression and Function 4
Bacterial Infection Pathways 9
NRAGE signals death through JNK 10
p75 NTR receptor-mediated signalling 25
Cell death signalling via NRAGE, NRIF and NADE 16
G alpha (12/13) signalling events 25
Death Receptor Signaling 31
RHOA GTPase cycle 18
CDC42 GTPase cycle 21
Signaling by ALK 12
NOTCH1 Intracellular Domain Regulates Transcription 17
Interleukin-2 family signaling 6
Signaling by NTRK3 (TRKC) 7
Activated NTRK3 signals through PI3K_1 4
STAT3 nuclear events downstream of ALK signaling_1 6
Signaling by LTK_1 4
G-protein mediated events 21
PLC beta mediated events 21
Inositol phosphate metabolism 7
Synthesis of IP3 and IP4 in the cytosol 7
Acetylcholine regulates insulin secretion 8
Free fatty acids regulate insulin secretion 7
Fatty Acids bound to GPR40 (FFAR1) regulate insul... 7
RIPK1-mediated regulated necrosis 3
Regulated Necrosis 8
Programmed Cell Death 26
Pyroptosis 5
TP53 Regulates Transcription of Cell Death Genes 6
Regulation of necroptotic cell death 3
TP53 Regulates Transcription of Death Receptors and Ligands 3
Synthesis of PC 3

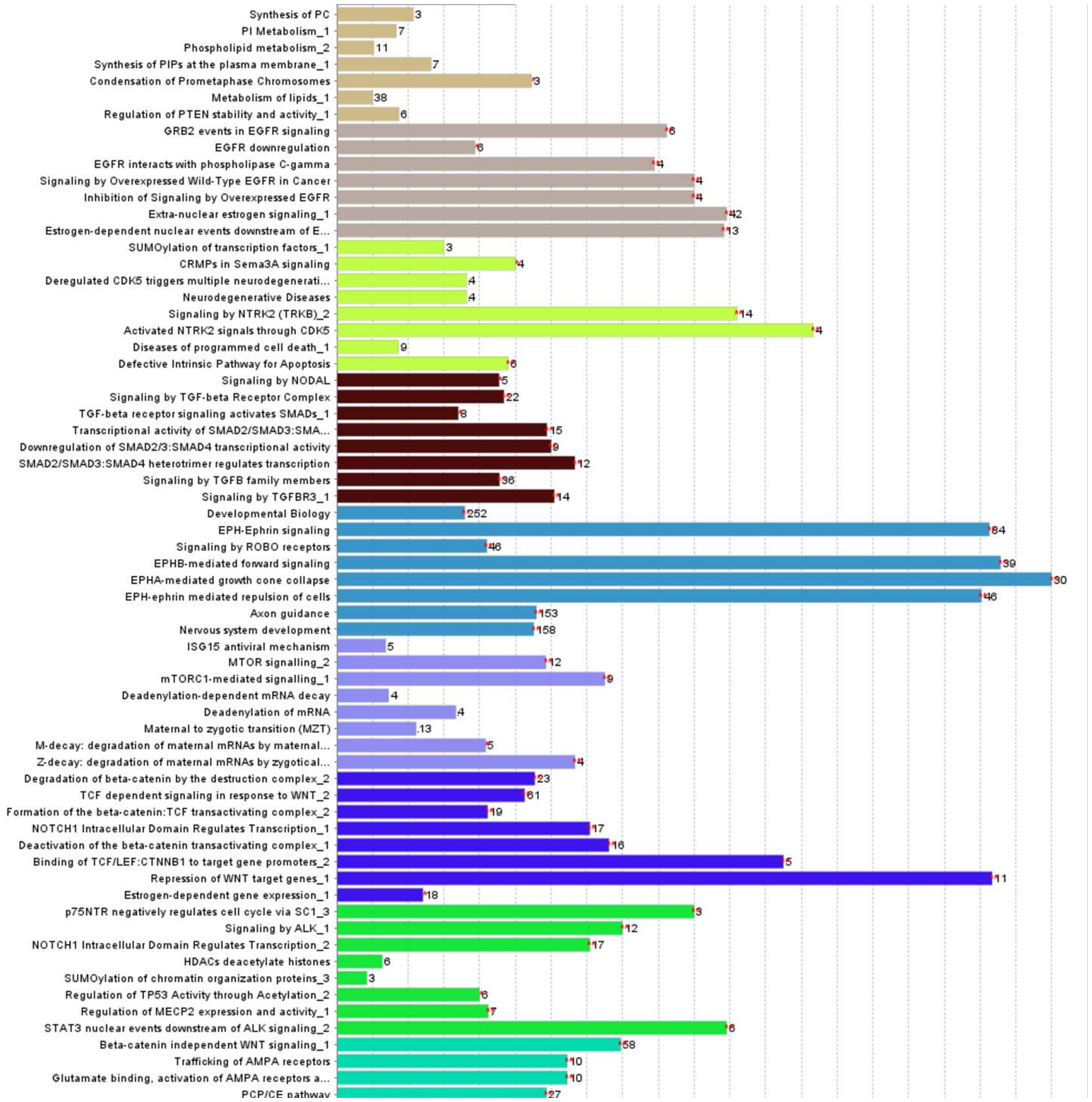
Synthesis of PC 3
PI Metabolism_1 7
Phospholipid metabolism_2 11
Synthesis of PIPs at the plasma membrane_1 7
Condensation of Prometaphase Chromosomes 3
Metabolism of lipids_1 38
Regulation of PTEN stability and activity_1 6
GRB2 events in EGFR signaling 6
EGFR downregulation 6
EGFR interacts with phospholipase C-gamma 4
Signaling by Overexpressed Wild-Type EGFR in Cancer 4
Inhibition of Signaling by Overexpressed EGFR 4
Extra-nuclear estrogen signaling_1 42
Estrogen-dependent nuclear events downstream of E... 13
SUMOylation of transcription factors_1 3
CRMPs in Sema3A signaling 4
Deregulated CDK5 triggers multiple neurodegenerati... 4
Neurodegenerative Diseases 4
Signaling by NTRK2 (TRKB)_2 14
Activated NTRK2 signals through CDK5 4
Diseases of programmed cell death_1 9
Defective Intrinsic Pathway for Apoptosis 6
Signaling by NODAL 5
Signaling by TGF-beta Receptor Complex 22
TGF-beta receptor signaling activates SMADs_1 8
Transcriptional activity of SMAD2/SMAD3:SMA... 15
Downregulation of SMAD2/3:SMAD4 transcriptional activity 9
SMAD2/SMAD3:SMAD4 heterotrimer regulates transcription 12
Signaling by TGFB family members 36
Signaling by TGFBR3_1 14
Developmental Biology 252
EPH-Ephrin signaling 84
Signaling by ROBO receptors 46
EPHB-mediated forward signaling 39
EPHA-mediated growth cone collapse 30
EPH-ephrin mediated repulsion of cells 46
Axon guidance 153
Nervous system development 158
ISG15 antiviral mechanism 5
MTOR signalling_2 12
mTORC1-mediated signalling_1 9
Deadenylation-dependent mRNA decay 4
Deadenylation of mRNA 4
Maternal to zygotic transition (MZT) 13
M-decay: degradation of maternal mRNAs by maternal... 5
Z-decay: degradation of maternal mRNAs by zygotical... 4
Degradation of beta-catenin by the destruction complex_2 23
TCF dependent signaling in response to WNT_2 61
Formation of the beta-catenin:TCF transactivating complex_2 19
NOTCH1 Intracellular Domain Regulates Transcription_1 17
Deactivation of the beta-catenin transactivating complex_1 16
Binding of TCF/LEF:CTNNB1 to target gene promoters_2 5
Repression of WNT target genes_1 11
Estrogen-dependent gene expression_1 18
p75NTR negatively regulates cell cycle via SC1_3 3
Signaling by ALK_1 12
NOTCH1 Intracellular Domain Regulates Transcription_2 17
HDACs deacetylate histones 6
SUMOylation of chromatin organization proteins_3 3
Regulation of TP53 Activity through Acetylation_2 6
Regulation of MECP2 expression and activity_1 7
STAT3 nuclear events downstream of ALK signaling_2 6
Beta-catenin independent WNT signaling_1 58
Trafficking of AMPA receptors 10
Glutamate binding, activation of AMPA receptors a... 10
PCP/CE pathway 27

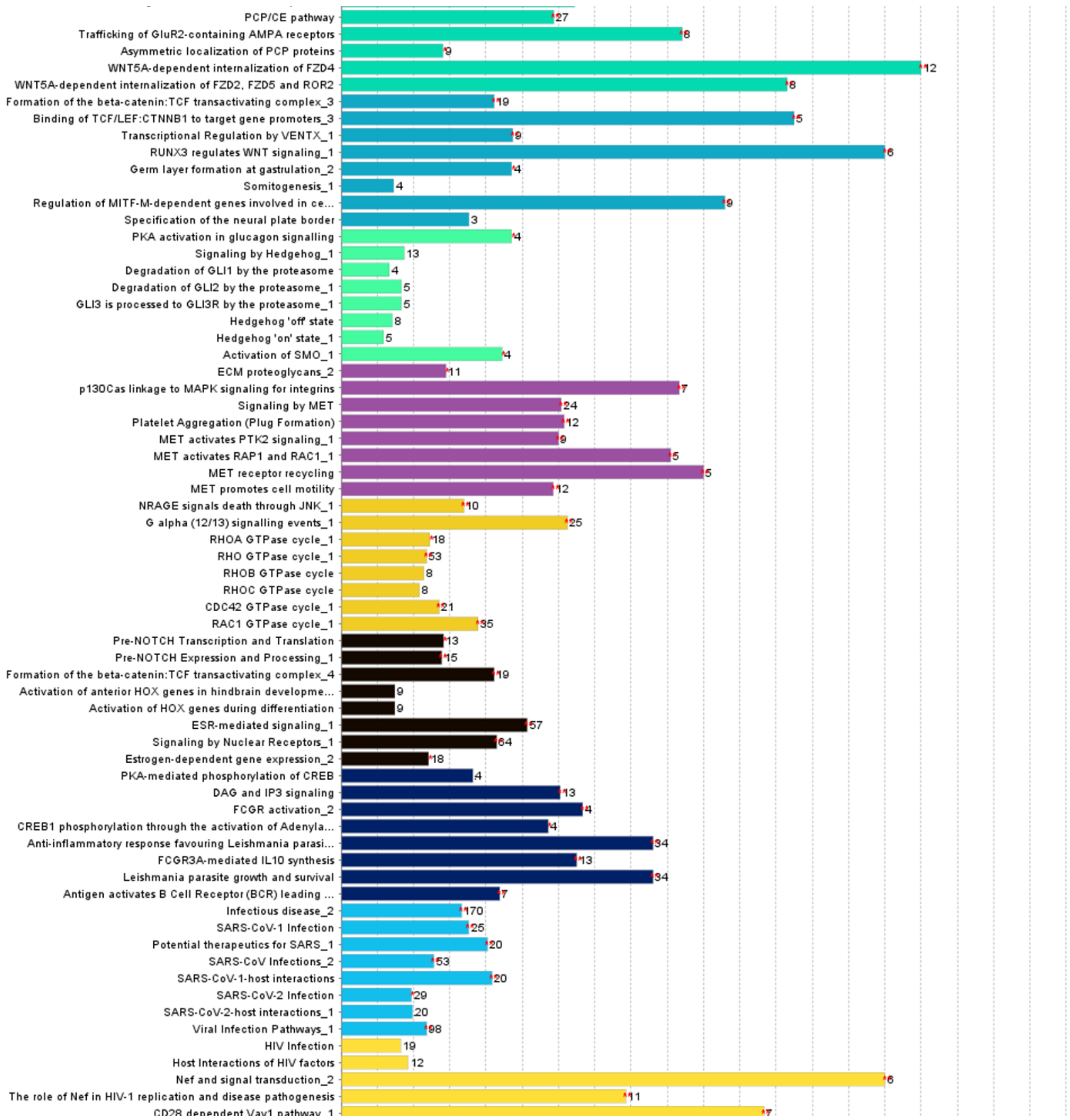
PCP/CE pathway 27
Trafficking of GluR2-containing AMPA receptors 8
Asymmetric localization of PCP proteins 9
WNT5A-dependent internalization of FZD4 12
WNT5A-dependent internalization of FZD2, FZD5 and ROR2 8
Formation of the beta-catenin:TCF transactivating complex_3 19
Binding of TCF/LEF:CTNNB1 to target gene promoters_3 5
Transcriptional Regulation by VENTX_1 9
RUNX3 regulates WNT signaling_1 6
Germ layer formation at gastrulation_2 4
Somitogenesis_1 4
Regulation of MITF-M-dependent genes involved in ce... 9
Specification of the neural plate border 3
PKA activation in glucagon signalling 4
Signaling by Hedgehog_1 13
Degradation of GLI1 by the proteasome 4
Degradation of GLI2 by the proteasome_1 5
GLI3 is processed to GLI3R by the proteasome_1 5
Hedgehog 'off' state 8
Hedgehog 'on' state_1 5
Activation of SMO_1 4
ECM proteoglycans_2 11
p130Cas linkage to MAPK signaling for integrins 7
Signaling by MET 24
Platelet Aggregation (Plug Formation) 12
MET activates PTK2 signaling_1 9
MET activates RAP1 and RAC1_1 5
MET receptor recycling 5
MET promotes cell motility 12
NRAGE signals death through JNK_1 10
G alpha (12/13) signalling events_1 25
RHOA GTPase cycle_1 18
RHO GTPase cycle_1 53
RHOB GTPase cycle 8
RHOC GTPase cycle 8
CDC42 GTPase cycle_1 21
RAC1 GTPase cycle_1 35
Pre-NOTCH Transcription and Translation 13
Pre-NOTCH Expression and Processing_1 15
Formation of the beta-catenin:TCF transactivating complex_4 19
Activation of anterior HOX genes in hindbrain developme... 9
Activation of HOX genes during differentiation 9
ESR-mediated signaling_1 57
Signaling by Nuclear Receptors_1 64
Estrogen-dependent gene expression_2 18
PKA-mediated phosphorylation of CREB 4
DAG and IP3 signaling 13
FCGR activation_2 4
CREB1 phosphorylation through the activation of Adenyla... 4
Anti-inflammatory response favouring Leishmania parasi... 34
FCGR3A-mediated IL10 synthesis 13
Leishmania parasite growth and survival 34
Antigen activates B Cell Receptor (BCR) leading ... 7
Infectious disease_2 170
SARS-CoV-1 Infection 25
Potential therapeutics for SARS_1 20
SARS-CoV Infections_2 53
SARS-CoV-1-host interactions 20
SARS-CoV-2 Infection 29
SARS-CoV-2-host interactions_1 20
Viral Infection Pathways_1 98
HIV Infection 19
Host Interactions of HIV factors 12
Nef and signal transduction_2 6
The role of Nef in HIV-1 replication and disease pathogenesis 11
CD28 dependent Vav1 pathway_1 7

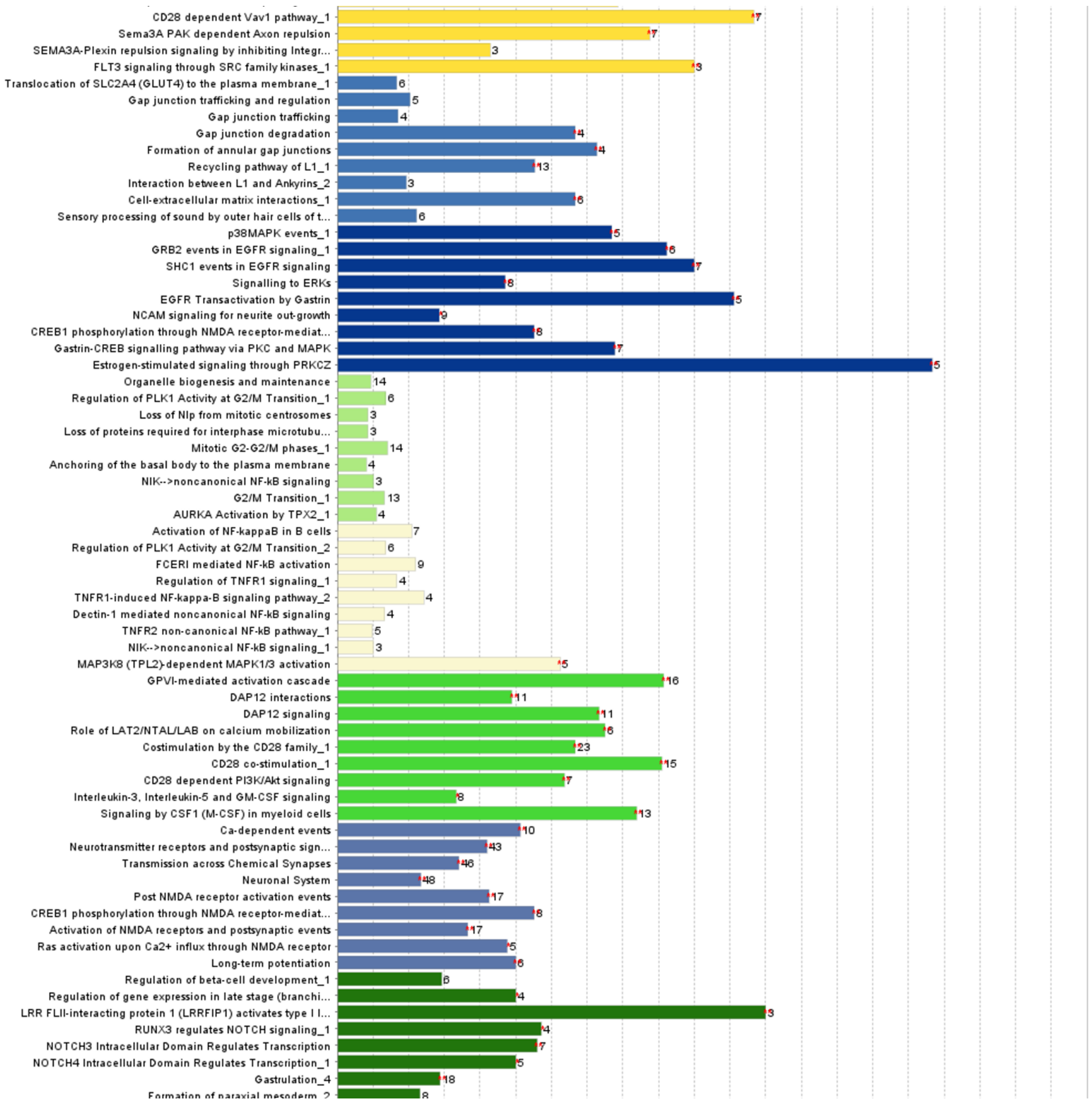
CD28 dependent Vav1 pathway_1
Sema3A PAK dependent Axon repulsion
SEMA3A-Plexin repulsion signaling by inhibiting Integr...
FLT3 signaling through SRC family kinases_1
Translocation of SLC2A4 (GLUT4) to the plasma membrane_1
Gap junction trafficking and regulation
Gap junction trafficking
Gap junction degradation
Formation of annular gap junctions
Recycling pathway of L1_1
Interaction between L1 and Ankyrins_2
Cell-extracellular matrix interactions_1
Sensory processing of sound by outer hair cells of t...
p38MAPK events_1
GRB2 events in EGFR signaling_1
SHC1 events in EGFR signaling
Signalling to ERKs
EGFR Transactivation by Gastrin
NCAM signaling for neurite out-growth
CREB1 phosphorylation through NMDA receptor-mediat...
Gastrin-CREB signalling pathway via PKC and MAPK
Estrogen-stimulated signaling through PRKCZ
Organelle biogenesis and maintenance
Regulation of PLK1 Activity at G2/M Transition_1
Loss of Nlp from mitotic centrosomes
Loss of proteins required for interphase microtubu...
Mitotic G2-G2/M phases_1
Anchoring of the basal body to the plasma membrane
NIK-->noncanonical NF-kB signaling
G2/M Transition_1
AURKA Activation by TPX2_1
Activation of NF-kappaB in B cells
Regulation of PLK1 Activity at G2/M Transition_2
FCERI mediated NF-kB activation
Regulation of TNFR1 signaling_1
TNFR1-induced NF-kappa-B signaling pathway_2
Dectin-1 mediated noncanonical NF-kB signaling
TNFR2 non-canonical NF-kB pathway_1
NIK-->noncanonical NF-kB signaling_1
MAP3K8 (TPL2)-dependent MAPK1/3 activation
GPVI-mediated activation cascade
DAP12 interactions
DAP12 signaling
Role of LAT2/NTAL/LAB on calcium mobilization
Costimulation by the CD28 family_1
CD28 co-stimulation_1
CD28 dependent PI3K/Akt signaling
Interleukin-3, Interleukin-5 and GM-CSF signaling
Signaling by CSF1 (M-CSF) in myeloid cells
Ca-dependent events
Neurotransmitter receptors and postsynaptic sign...
Transmission across Chemical Synapses
Neuronal System
Post NMDA receptor activation events
CREB1 phosphorylation through NMDA receptor-mediat...
Activation of NMDA receptors and postsynaptic events
Ras activation upon Ca2+ influx through NMDA receptor
Long-term potentiation
Regulation of beta-cell development_1
Regulation of gene expression in late stage (branchi...
LRR FLII-interacting protein 1 (LRRFIP1) activates type I I...
RUNX3 regulates NOTCH signaling_1
NOTCH3 Intracellular Domain Regulates Transcription
NOTCH4 Intracellular Domain Regulates Transcription_1
Gastrulation_4
Formation of paraxial mesoderm_2

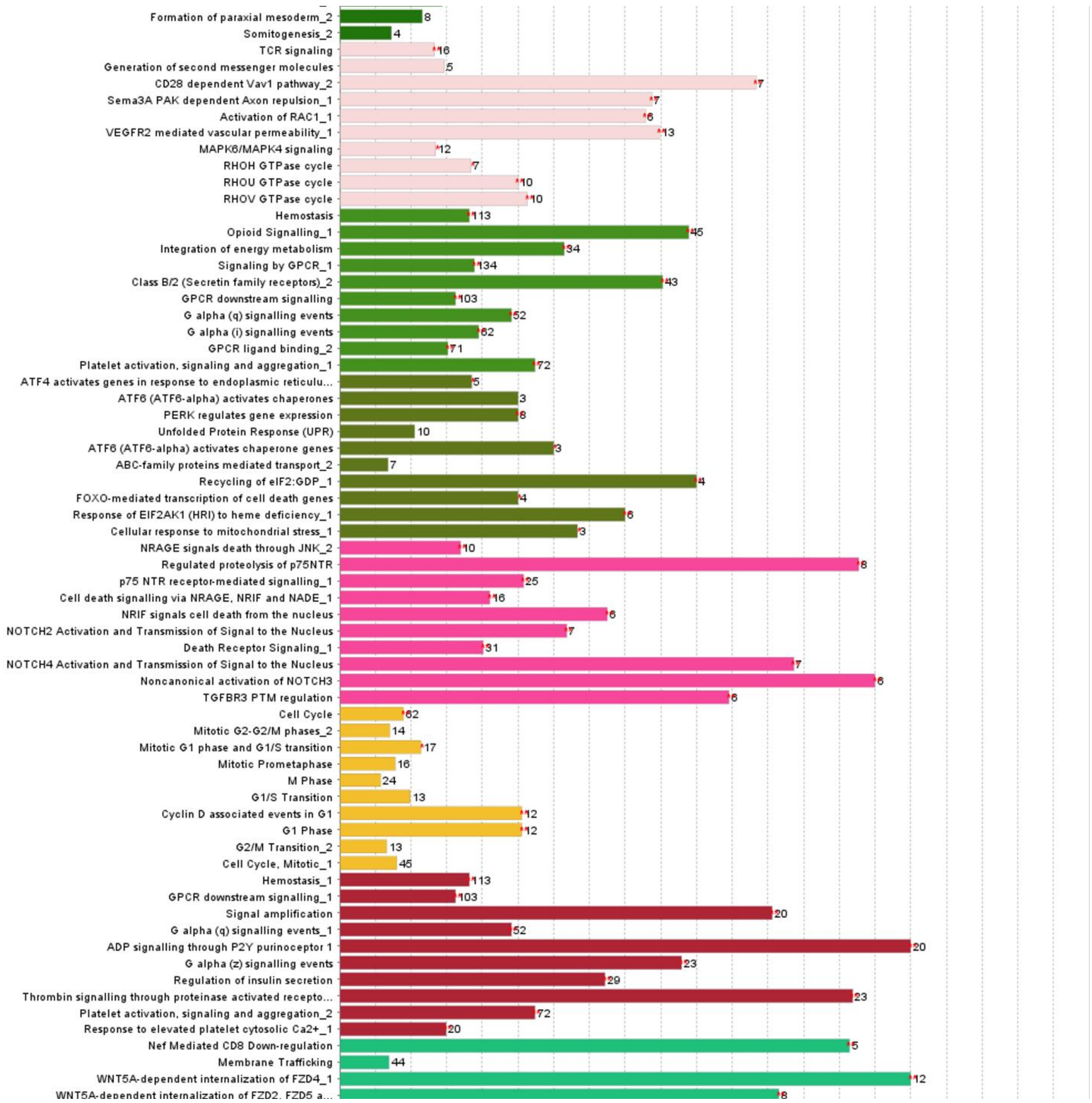
Formation of paraxial mesoderm_2
Somitogenesis_2
TCR signaling
Generation of second messenger molecules
CD28 dependent Vav1 pathway_2
Sema3A PAK dependent Axon repulsion_1
Activation of RAC1_1
VEGFR2 mediated vascular permeability_1
MAPK6/MAPK4 signaling
RHOH GTPase cycle
RHOU GTPase cycle
RHOV GTPase cycle
Hemostasis
Opioid Signalling_1
Integration of energy metabolism
Signaling by GPCR_1
Class B/2 (Secretin family receptors)_2
GPCR downstream signalling
G alpha (q) signalling events
G alpha (i) signalling events
GPCR ligand binding_2
Platelet activation, signaling and aggregation_1
ATF4 activates genes in response to endoplasmic reticulu...
ATF6 (ATF6-alpha) activates chaperones
PERK regulates gene expression
Unfolded Protein Response (UPR)
ATF6 (ATF6-alpha) activates chaperone genes
ABC-family proteins mediated transport_2
Recycling of eIF2:GDP_1
FOXO-mediated transcription of cell death genes
Response of EIF2AK1 (HRI) to heme deficiency_1
Cellular response to mitochondrial stress_1
NRAGE signals death through JNK_2
Regulated proteolysis of p75NTR
p75 NTR receptor-mediated signalling_1
Cell death signalling via NRAGE, NRIF and NADE_1
NRIF signals cell death from the nucleus
NOTCH2 Activation and Transmission of Signal to the Nucleus
Death Receptor Signaling_1
NOTCH4 Activation and Transmission of Signal to the Nucleus
Noncanonical activation of NOTCH3
TGFBR3 PTM regulation
Cell Cycle
Mitotic G2-G2/M phases_2
Mitotic G1 phase and G1/S transition
Mitotic Prometaphase
M Phase
G1/S Transition
Cyclin D associated events in G1
G1 Phase
G2/M Transition_2
Cell Cycle, Mitotic_1
Hemostasis_1
GPCR downstream signalling_1
Signal amplification
G alpha (q) signalling events_1
ADP signalling through P2Y purinoceptor 1
G alpha (z) signalling events
Regulation of insulin secretion
Thrombin signalling through proteinase activated recepto...
Platelet activation, signaling and aggregation_2
Response to elevated platelet cytosolic Ca2+_1
Nef Mediated CD8 Down-regulation
Membrane Trafficking
WNT5A-dependent internalization of FZD4_1
WNT5A-dependent internalization of FZD2, FZD5 a...

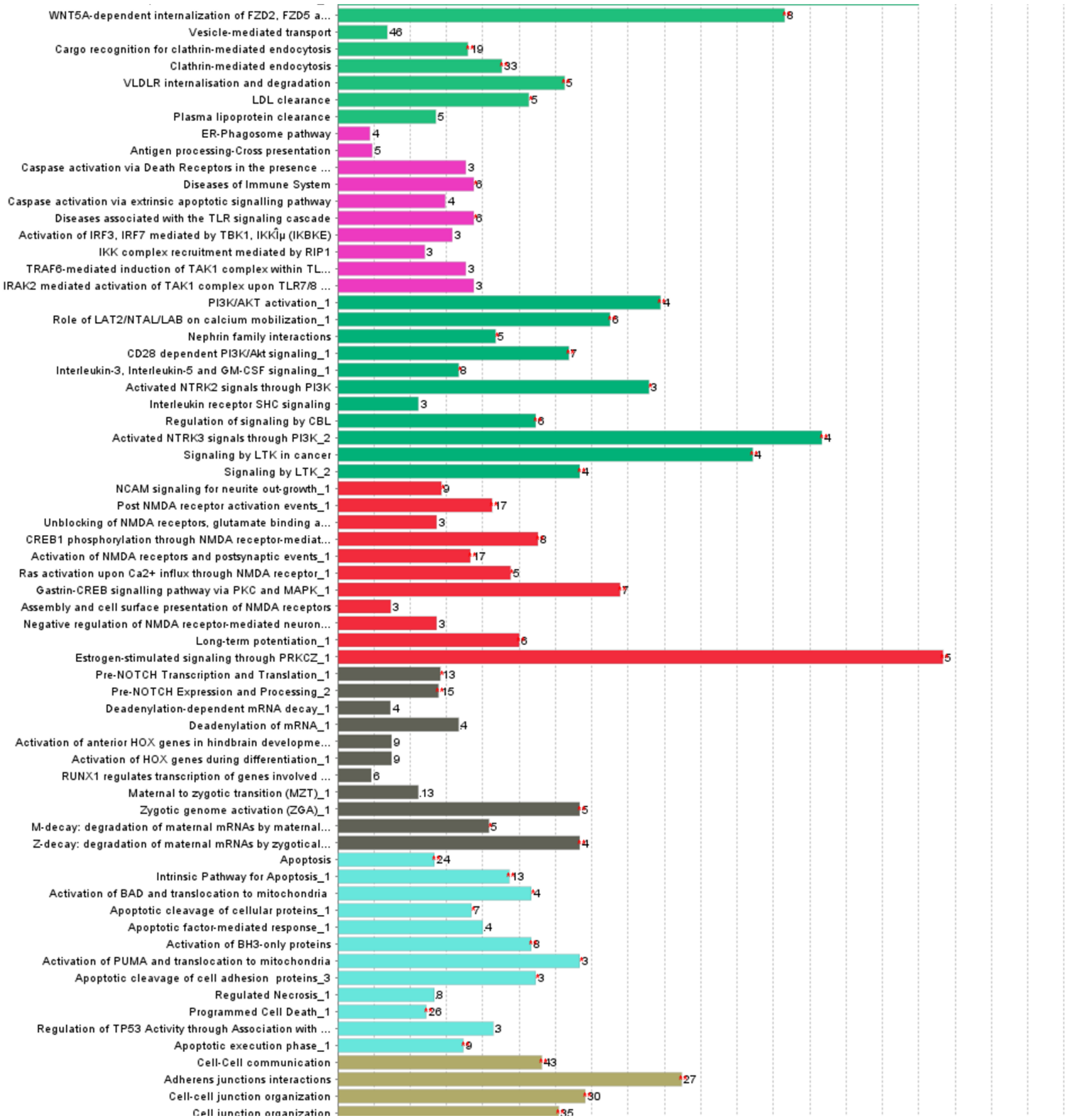
WNT5A-dependent internalization of FZD2, FZD5 a... 8
Vesicle-mediated transport 46
Cargo recognition for clathrin-mediated endocytosis 19
Clathrin-mediated endocytosis 33
VLDLR internalisation and degradation 5
LDL clearance 5
Plasma lipoprotein clearance 5
ER-Phagosome pathway 4
Antigen processing-Cross presentation 5
Caspase activation via Death Receptors in the presence ... 3
Diseases of Immune System 6
Caspase activation via extrinsic apoptotic signalling pathway 4
Diseases associated with the TLR signaling cascade 6
Activation of IRF3, IRF7 mediated by TBK1, IKKÎµ (IKBKE) 3
IKK complex recruitment mediated by RIP1 3
TRAF6-mediated induction of TAK1 complex within TL... 3
IRAK2 mediated activation of TAK1 complex upon TLR7/8 ... 3
PI3K/AKT activation_1 4
Role of LAT2/NTAL/LAB on calcium mobilization_1 6
Nephrin family interactions 5
CD28 dependent PI3K/Akt signaling_1 7
Interleukin-3, Interleukin-5 and GM-CSF signaling_1 8
Activated NTRK2 signals through PI3K 3
Interleukin receptor SHC signaling 3
Regulation of signaling by CBL 6
Activated NTRK3 signals through PI3K_2 4
Signaling by LTK in cancer 4
Signaling by LTK_2 4
NCAM signaling for neurite out-growth_1 9
Post NMDA receptor activation events_1 17
Unblocking of NMDA receptors, glutamate binding a... 3
CREB1 phosphorylation through NMDA receptor-mediat... 8
Activation of NMDA receptors and postsynaptic events_1 17
Ras activation upon Ca2+ influx through NMDA receptor_1 5
Gastrin-CREB signalling pathway via PKC and MAPK_1 7
Assembly and cell surface presentation of NMDA receptors 3
Negative regulation of NMDA receptor-mediated neuron... 3
Long-term potentiation_1 6
Estrogen-stimulated signaling through PRKCZ_1 5
Pre-NOTCH Transcription and Translation_1 13
Pre-NOTCH Expression and Processing_2 15
Deadenylation-dependent mRNA decay_1 4
Deadenylation of mRNA_1 4
Activation of anterior HOX genes in hindbrain developme... 9
Activation of HOX genes during differentiation_1 9
RUNX1 regulates transcription of genes involved ... 6
Maternal to zygotic transition (MZT)_1 13
Zygotic genome activation (ZGA)_1 5
M-decay: degradation of maternal mRNAs by maternal... 5
Z-decay: degradation of maternal mRNAs by zygotical... 4
Apoptosis 24
Intrinsic Pathway for Apoptosis_1 13
Activation of BAD and translocation to mitochondria 4
Apoptotic cleavage of cellular proteins_1 7
Apoptotic factor-mediated response_1 4
Activation of BH3-only proteins 8
Activation of PUMA and translocation to mitochondria 3
Apoptotic cleavage of cell adhesion proteins_3 3
Regulated Necrosis_1 8
Programmed Cell Death_1 26
Regulation of TP53 Activity through Association with ... 3
Apoptotic execution phase_1 9
Cell-Cell communication 43
Adherens junctions interactions 27
Cell-cell junction organization 30
Cell junction organization 35

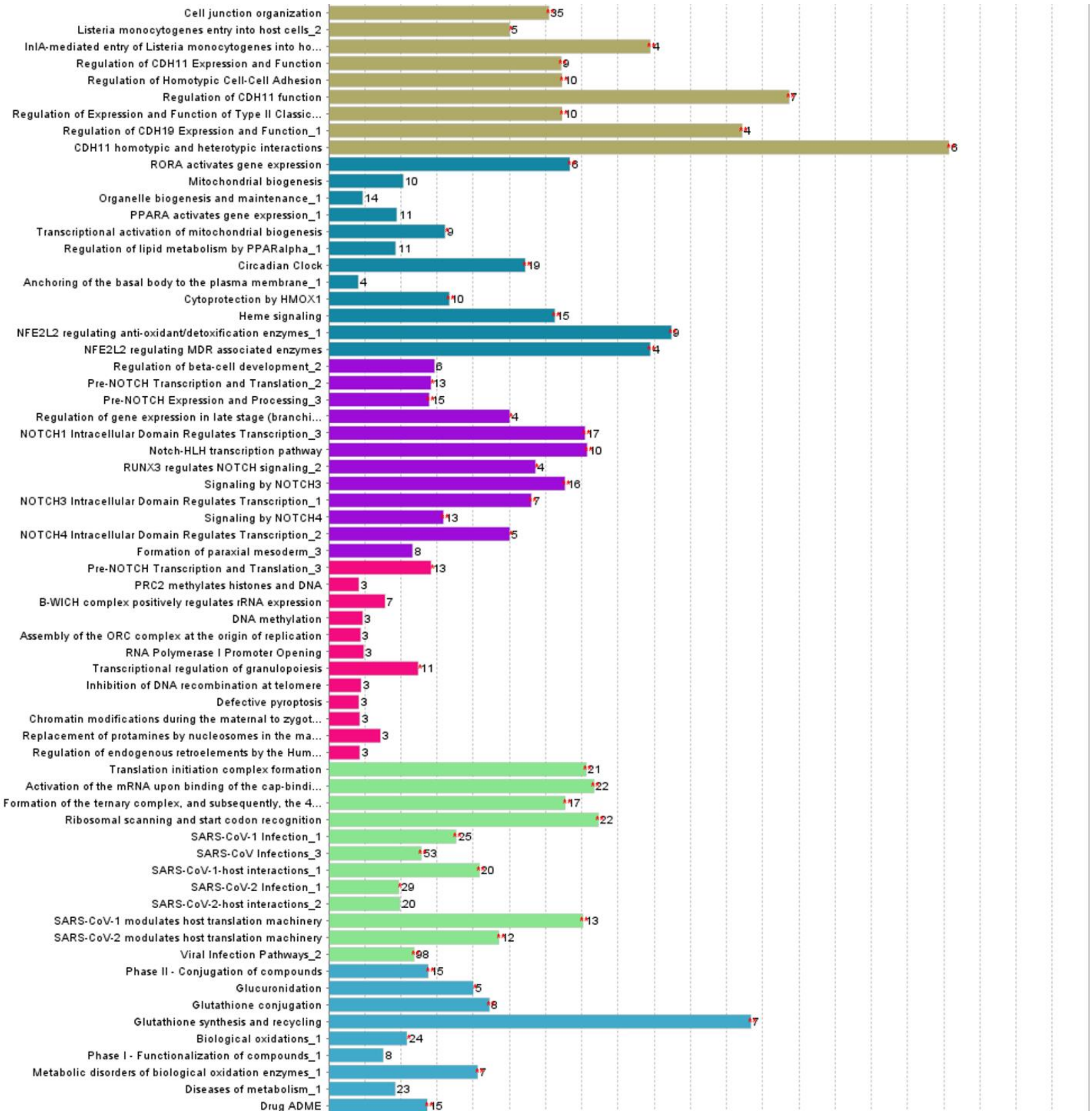

Cell junction organization 35
Listeria monocytogenes entry into host cells_2 5
InlA-mediated entry of Listeria monocytogenes into ho... 4
Regulation of CDH11 Expression and Function 9
Regulation of Homotypic Cell-Cell Adhesion 10
Regulation of CDH11 function 7
Regulation of Expression and Function of Type II Classic... 10
Regulation of CDH19 Expression and Function_1 4
CDH11 homotypic and heterotypic interactions 6
RORA activates gene expression 6
Mitochondrial biogenesis 10
Organelle biogenesis and maintenance_1 14
PPARA activates gene expression_1 11
Transcriptional activation of mitochondrial biogenesis 9
Regulation of lipid metabolism by PPARalpha_1 11
Circadian Clock 19
Anchoring of the basal body to the plasma membrane_1 4
Cytoprotection by HMOX1 10
Heme signaling 15
NFE2L2 regulating anti-oxidant/detoxification enzymes_1 9
NFE2L2 regulating MDR associated enzymes 4
Regulation of beta-cell development_2 6
Pre-NOTCH Transcription and Translation_2 13
Pre-NOTCH Expression and Processing_3 15
Regulation of gene expression in late stage (branchi... 4
NOTCH1 Intracellular Domain Regulates Transcription_3 17
Notch-HLH transcription pathway 10
RUNX3 regulates NOTCH signaling_2 4
Signaling by NOTCH3 16
NOTCH3 Intracellular Domain Regulates Transcription_1 7
Signaling by NOTCH4 13
NOTCH4 Intracellular Domain Regulates Transcription_2 5
Formation of paraxial mesoderm_3 8
Pre-NOTCH Transcription and Translation_3 13
PRC2 methylates histones and DNA 3
B-WICH complex positively regulates rRNA expression 7
DNA methylation 3
Assembly of the ORC complex at the origin of replication 3
RNA Polymerase I Promoter Opening 3
Transcriptional regulation of granulopoiesis 11
Inhibition of DNA recombination at telomere 3
Defective pyroptosis 3
Chromatin modifications during the maternal to zygot... 3
Replacement of protamines by nucleosomes in the ma... 3
Regulation of endogenous retroelements by the Hum... 3
Translation initiation complex formation 21
Activation of the mRNA upon binding of the cap-bindi... 22
Formation of the ternary complex, and subsequently, the 4... 17
Ribosomal scanning and start codon recognition 22
SARS-CoV-1 Infection_1 25
SARS-CoV Infections_3 53
SARS-CoV-1-host interactions_1 20
SARS-CoV-2 Infection_1 29
SARS-CoV-2-host interactions_2 20
SARS-CoV-1 modulates host translation machinery 13
SARS-CoV-2 modulates host translation machinery 12
Viral Infection Pathways_2 98
Phase II - Conjugation of compounds 15
Glucuronidation 5
Glutathione conjugation 8
Glutathione synthesis and recycling 7
Biological oxidations_1 24
Phase I - Functionalization of compounds_1 8
Metabolic disorders of biological oxidation enzymes_1 7
Diseases of metabolism_1 23
Drug ADME 15

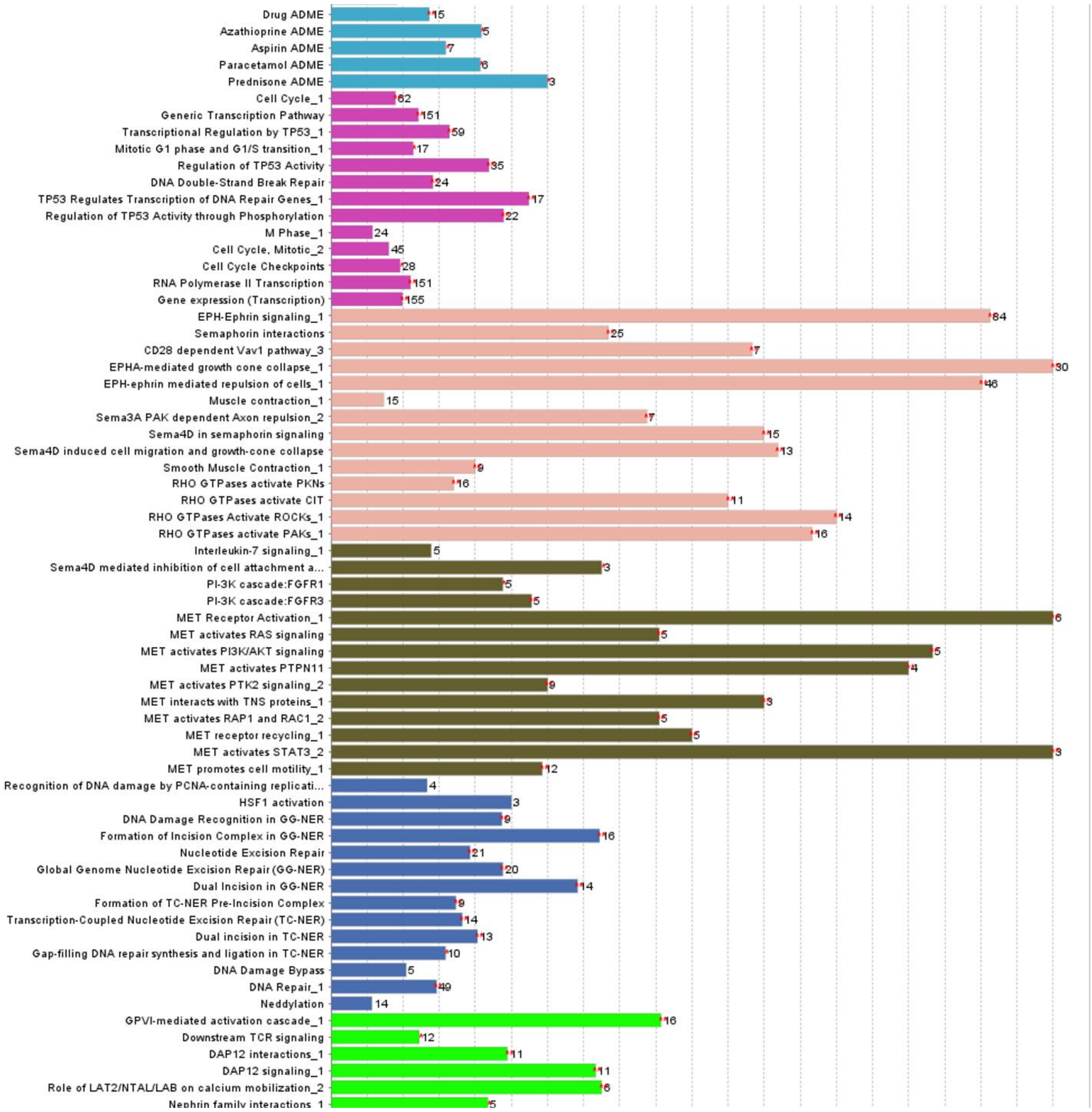
Drug ADME 15
Azathioprine ADME 5
Aspirin ADME 7
Paracetamol ADME 6
Prednisone ADME 3
Cell Cycle_1 62
Generic Transcription Pathway 151
Transcriptional Regulation by TP53_1 59
Mitotic G1 phase and G1/S transition_1 17
Regulation of TP53 Activity 35
DNA Double-Strand Break Repair 24
TP53 Regulates Transcription of DNA Repair Genes_1 17
Regulation of TP53 Activity through Phosphorylation 22
M Phase_1 24
Cell Cycle, Mitotic_2 45
Cell Cycle Checkpoints 28
RNA Polymerase II Transcription 151
Gene expression (Transcription) 155
EPH-Ephrin signaling_1 84
Semaphorin interactions 25
CD28 dependent Vav1 pathway_3 7
EPHA-mediated growth cone collapse_1 30
EPH-ephrin mediated repulsion of cells_1 46
Muscle contraction_1 15
Sema3A PAK dependent Axon repulsion_2 7
Sema4D in semaphorin signaling 15
Sema4D induced cell migration and growth-cone collapse 13
Smooth Muscle Contraction_1 9
RHO GTPases activate PKNs 16
RHO GTPases activate CIT 11
RHO GTPases Activate ROCKs_1 14
RHO GTPases activate PAKs_1 16
Interleukin-7 signaling_1 5
Sema4D mediated inhibition of cell attachment a... 3
PI-3K cascade:FGFR1 5
PI-3K cascade:FGFR3 5
MET Receptor Activation_1 6
MET activates RAS signaling 5
MET activates PI3K/AKT signaling 5
MET activates PTPN11 4
MET activates PTK2 signaling_2 9
MET interacts with TNS proteins_1 3
MET activates RAP1 and RAC1_2 5
MET receptor recycling_1 5
MET activates STAT3_2 3
MET promotes cell motility_1 12
Recognition of DNA damage by PCNA-containing replicati... 4
HSF1 activation 3
DNA Damage Recognition in GG-NER 9
Formation of Incision Complex in GG-NER 16
Nucleotide Excision Repair 21
Global Genome Nucleotide Excision Repair (GG-NER) 20
Dual Incision in GG-NER 14
Formation of TC-NER Pre-Incision Complex 9
Transcription-Coupled Nucleotide Excision Repair (TC-NER) 14
Dual incision in TC-NER 13
Gap-filling DNA repair synthesis and ligation in TC-NER 10
DNA Damage Bypass 5
DNA Repair_1 49
Neddylation 14
GPVI-mediated activation cascade_1 16
Downstream TCR signaling 12
DAP12 interactions_1 11
DAP12 signaling_1 11
Role of LAT2/NTAL/LAB on calcium mobilization_2 6
Nephrin family interactions_1 5

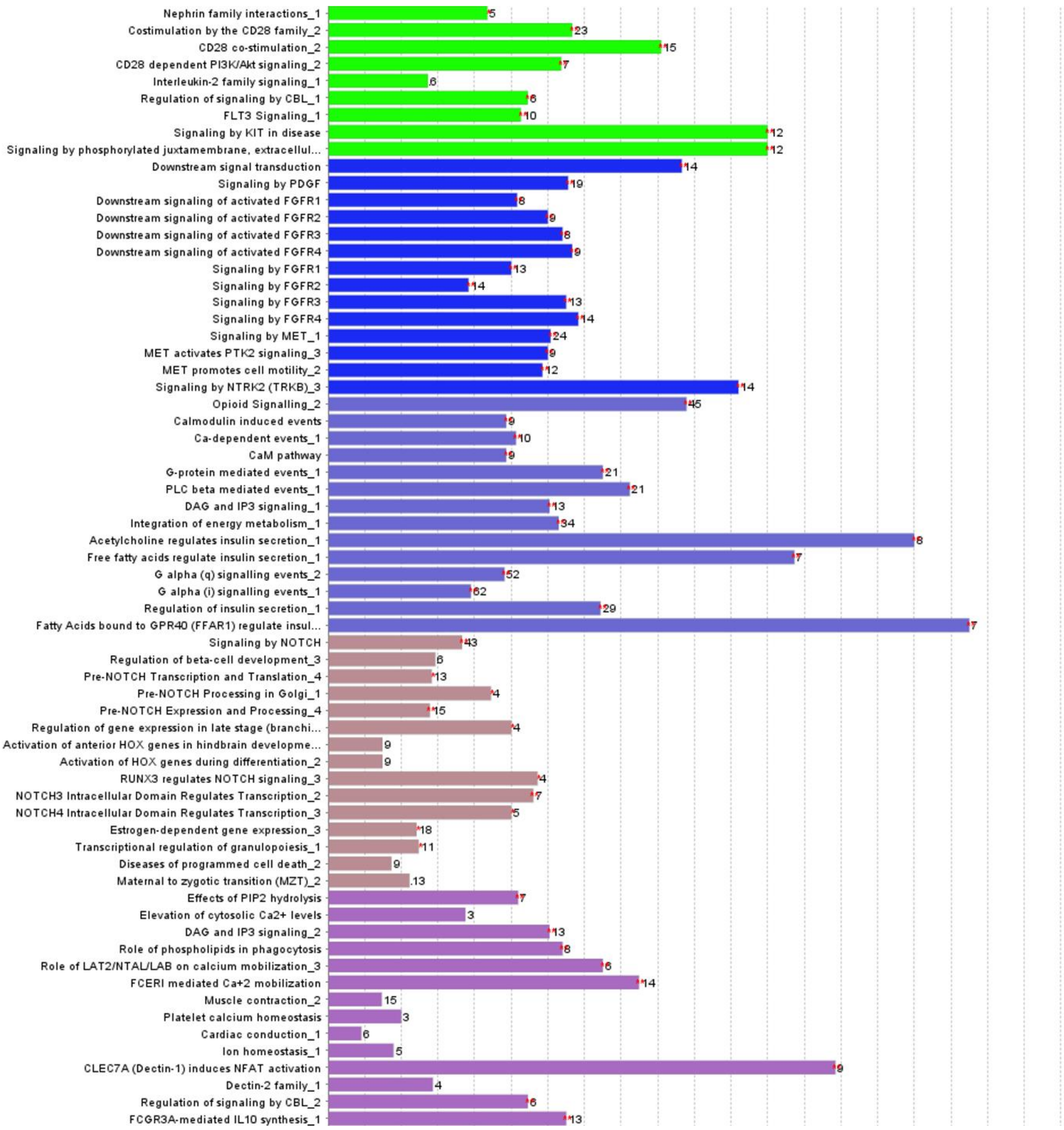

Nephrin family interactions_1
5
Costimulation by the CD28 family_2
23
CD28 co-stimulation_2
15
CD28 dependent PI3K/Akt signaling_2
7
Interleukin-2 family signaling_1
6
Regulation of signaling by CBL_1
6
FLT3 Signaling_1
10
Signaling by KIT in disease
12
Signaling by phosphorylated juxtamembrane, extracellul...
12
Downstream signal transduction
14
Signaling by PDGF
19
Downstream signaling of activated FGFR1
8
Downstream signaling of activated FGFR2
9
Downstream signaling of activated FGFR3
8
Downstream signaling of activated FGFR4
9
Signaling by FGFR1
13
Signaling by FGFR2
14
Signaling by FGFR3
13
Signaling by FGFR4
14
Signaling by MET_1
24
MET activates PTK2 signaling_3
9
MET promotes cell motility_2
12
Signaling by NTRK2 (TRKB)_3
14
Opioid Signalling_2
45
Calmodulin induced events
9
Ca-dependent events_1
10
CaM pathway
9
G-protein mediated events_1
21
PLC beta mediated events_1
21
DAG and IP3 signaling_1
13
Integration of energy metabolism_1
34
Acetylcholine regulates insulin secretion_1
8
Free fatty acids regulate insulin secretion_1
7
G alpha (q) signalling events_2
52
G alpha (i) signalling events_1
62
Regulation of insulin secretion_1
29
Fatty Acids bound to GPR40 (FFAR1) regulate insul...
7
Signaling by NOTCH
43
Regulation of beta-cell development_3
8
Pre-NOTCH Transcription and Translation_4
13
Pre-NOTCH Processing in Golgi_1
4
Pre-NOTCH Expression and Processing_4
15
Regulation of gene expression in late stage (branchi...
4
Activation of anterior HOX genes in hindbrain developme...
9
Activation of HOX genes during differentiation_2
9
RUNX3 regulates NOTCH signaling_3
4
NOTCH3 Intracellular Domain Regulates Transcription_2
7
NOTCH4 Intracellular Domain Regulates Transcription_3
5
Estrogen-dependent gene expression_3
18
Transcriptional regulation of granulopoiesis_1
11
Diseases of programmed cell death_2
9
Maternal to zygotic transition (MZT)_2
13
Effects of PIP2 hydrolysis
7
Elevation of cytosolic Ca2+ levels
3
DAG and IP3 signaling_2
13
Role of phospholipids in phagocytosis
8
Role of LAT2/NTAL/LAB on calcium mobilization_3
6
FCERI mediated Ca+2 mobilization
14
Muscle contraction_2
15
Platelet calcium homeostasis
3
Cardiac conduction_1
6
Ion homeostasis_1
5
CLEC7A (Dectin-1) induces NFAT activation
9
Dectin-2 family_1
4
Regulation of signaling by CBL_2
6
FCGR3A-mediated IL10 synthesis_1
13

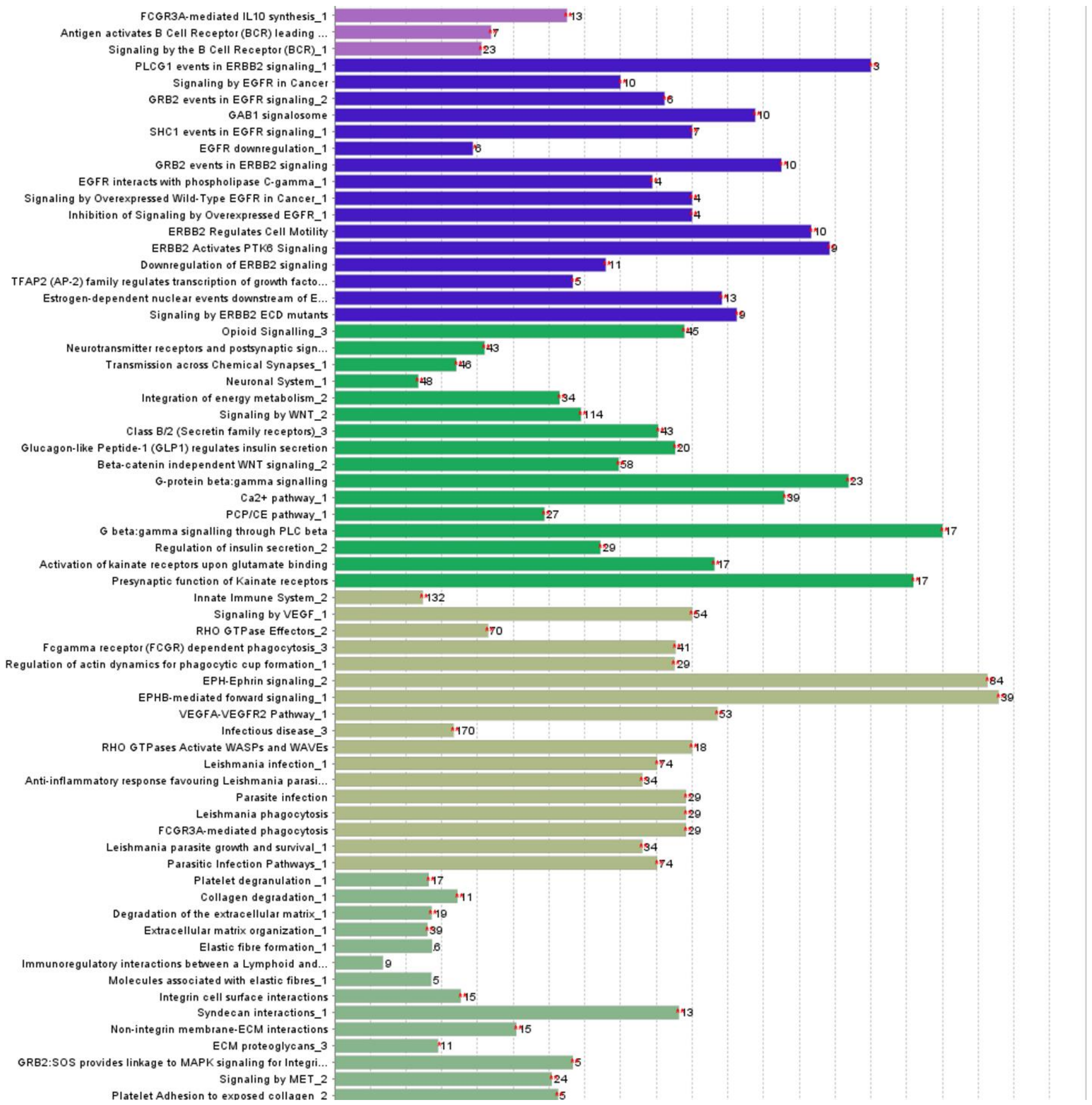
FCGR3A-mediated IL10 synthesis_1
Antigen activates B Cell Receptor (BCR) leading ...
Signaling by the B Cell Receptor (BCR)_1
PLCG1 events in ERBB2 signaling_1
Signaling by EGFR in Cancer
GRB2 events in EGFR signaling_2
GAB1 signalosome
SHC1 events in EGFR signaling_1
EGFR downregulation_1
GRB2 events in ERBB2 signaling
EGFR interacts with phospholipase C-gamma_1
Signaling by Overexpressed Wild-Type EGFR in Cancer_1
Inhibition of Signaling by Overexpressed EGFR_1
ERBB2 Regulates Cell Motility
ERBB2 Activates PTK6 Signaling
Downregulation of ERBB2 signaling
TFAP2 (AP-2) family regulates transcription of growth facto...
Estrogen-dependent nuclear events downstream of E...
Signaling by ERBB2 ECD mutants
Opioid Signalling_3
Neurotransmitter receptors and postsynaptic sign...
Transmission across Chemical Synapses_1
Neuronal System_1
Integration of energy metabolism_2
Signaling by WNT_2
Class B/2 (Secretin family receptors)_3
Glucagon-like Peptide-1 (GLP1) regulates insulin secretion
Beta-catenin independent WNT signaling_2
G-protein beta:gamma signalling
Ca2+ pathway_1
PCP/CE pathway_1
G beta:gamma signalling through PLC beta
Regulation of insulin secretion_2
Activation of kainate receptors upon glutamate binding
Presynaptic function of Kainate receptors
Innate Immune System_2
Signaling by VEGF_1
RHO GTPase Effectors_2
Fcgamma receptor (FCGR) dependent phagocytosis_3
Regulation of actin dynamics for phagocytic cup formation_1
EPH-Ephrin signaling_2
EPHB-mediated forward signaling_1
VEGFA-VEGFR2 Pathway_1
Infectious disease_3
RHO GTPases Activate WASPs and WAVEs
Leishmania infection_1
Anti-inflammatory response favouring Leishmania parasi...
Parasite infection
Leishmania phagocytosis
FCGR3A-mediated phagocytosis
Leishmania parasite growth and survival_1
Parasitic Infection Pathways_1
Platelet degranulation _1
Collagen degradation_1
Degradation of the extracellular matrix_1
Extracellular matrix organization_1
Elastic fibre formation_1
Immunoregulatory interactions between a Lymphoid and...
Molecules associated with elastic fibres_1
Integrin cell surface interactions
Syndecan interactions_1
Non-integrin membrane-ECM interactions
ECM proteoglycans_3
GRB2:SOS provides linkage to MAPK signaling for Integri...
Signaling by MET_2
Platelet Adhesion to exposed collagen_2
13
7
23
3
10
6
10
7
6
10
4
4
4
10
9
11
5
13
9
45
43
46
48
34
114
43
20
58
23
39
27
17
29
17
17
132
54
70
41
29
84
39
53
170
18
74
34
29
29
29
34
74
17
11
19
39
6
9
5
15
13
15
11
5
24
5

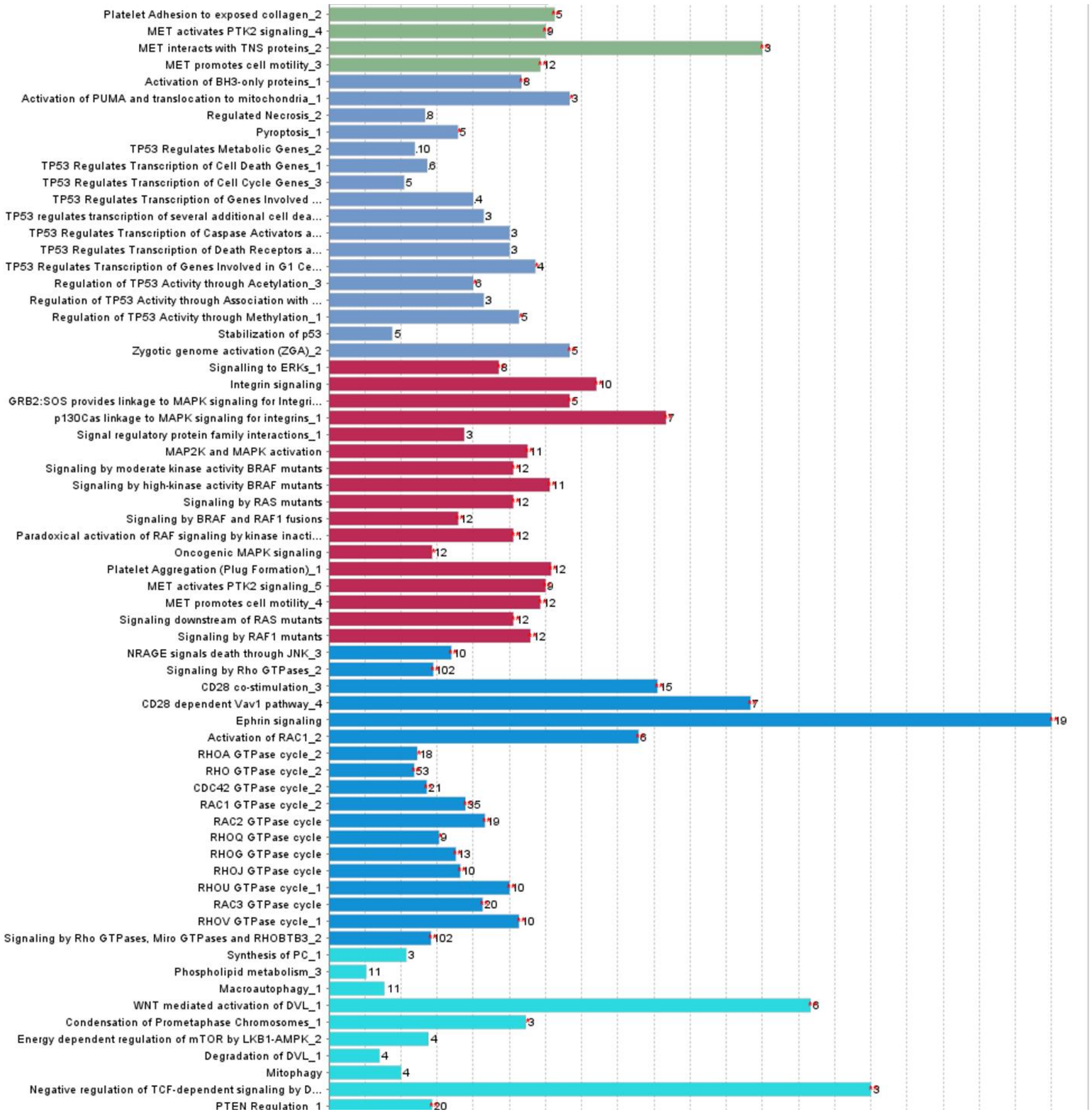
Platelet Adhesion to exposed collagen_2
MET activates PTK2 signaling_4
MET interacts with TNS proteins_2
MET promotes cell motility_3
Activation of BH3-only proteins_1
Activation of PUMA and translocation to mitochondria_1
Regulated Necrosis_2
Pyroptosis_1
TP53 Regulates Metabolic Genes_2
TP53 Regulates Transcription of Cell Death Genes_1
TP53 Regulates Transcription of Cell Cycle Genes_3
TP53 Regulates Transcription of Genes Involved ...
TP53 regulates transcription of several additional cell dea...
TP53 Regulates Transcription of Caspase Activators a...
TP53 Regulates Transcription of Death Receptors a...
TP53 Regulates Transcription of Genes Involved in G1 Ce...
Regulation of TP53 Activity through Acetylation_3
Regulation of TP53 Activity through Association with ...
Regulation of TP53 Activity through Methylation_1
Stabilization of p53
Zygotic genome activation (ZGA)_2
Signalling to ERKs_1
Integrin signaling
GRB2:SOS provides linkage to MAPK signaling for Integri...
p130Cas linkage to MAPK signaling for integrins_1
Signal regulatory protein family interactions_1
MAP2K and MAPK activation
Signaling by moderate kinase activity BRAF mutants
Signaling by high-kinase activity BRAF mutants
Signaling by RAS mutants
Signaling by BRAF and RAF1 fusions
Paradoxical activation of RAF signaling by kinase inacti...
Oncogenic MAPK signaling
Platelet Aggregation (Plug Formation)_1
MET activates PTK2 signaling_5
MET promotes cell motility_4
Signaling downstream of RAS mutants
Signaling by RAF1 mutants
NRAGE signals death through JNK_3
Signaling by Rho GTPases_2
CD28 co-stimulation_3
CD28 dependent Vav1 pathway_4
Ephrin signaling
Activation of RAC1_2
RHOA GTPase cycle_2
RHO GTPase cycle_2
CDC42 GTPase cycle_2
RAC1 GTPase cycle_2
RAC2 GTPase cycle
RHOQ GTPase cycle
RHOG GTPase cycle
RHOJ GTPase cycle
RHOU GTPase cycle_1
RAC3 GTPase cycle
RHOV GTPase cycle_1
Signaling by Rho GTPases, Miro GTPases and RHOBTB3_2
Synthesis of PC_1
Phospholipid metabolism_3
Macroautophagy_1
WNT mediated activation of DVL_1
Condensation of Prometaphase Chromosomes_1
Energy dependent regulation of mTOR by LKB1-AMPK_2
Degradation of DVL_1
Mitophagy
Negative regulation of TCF-dependent signaling by D...
PTEN Regulation_1
5
9
3
12
8
3
8
5
10
6
5
4
3
3
3
4
6
3
5
5
5
8
10
5
7
3
11
12
11
12
12
12
12
12
9
12
12
12
10
102
15
7
19
6
18
53
21
35
19
9
13
10
10
20
10
102
3
11
11
6
3
4
4
4
3
20

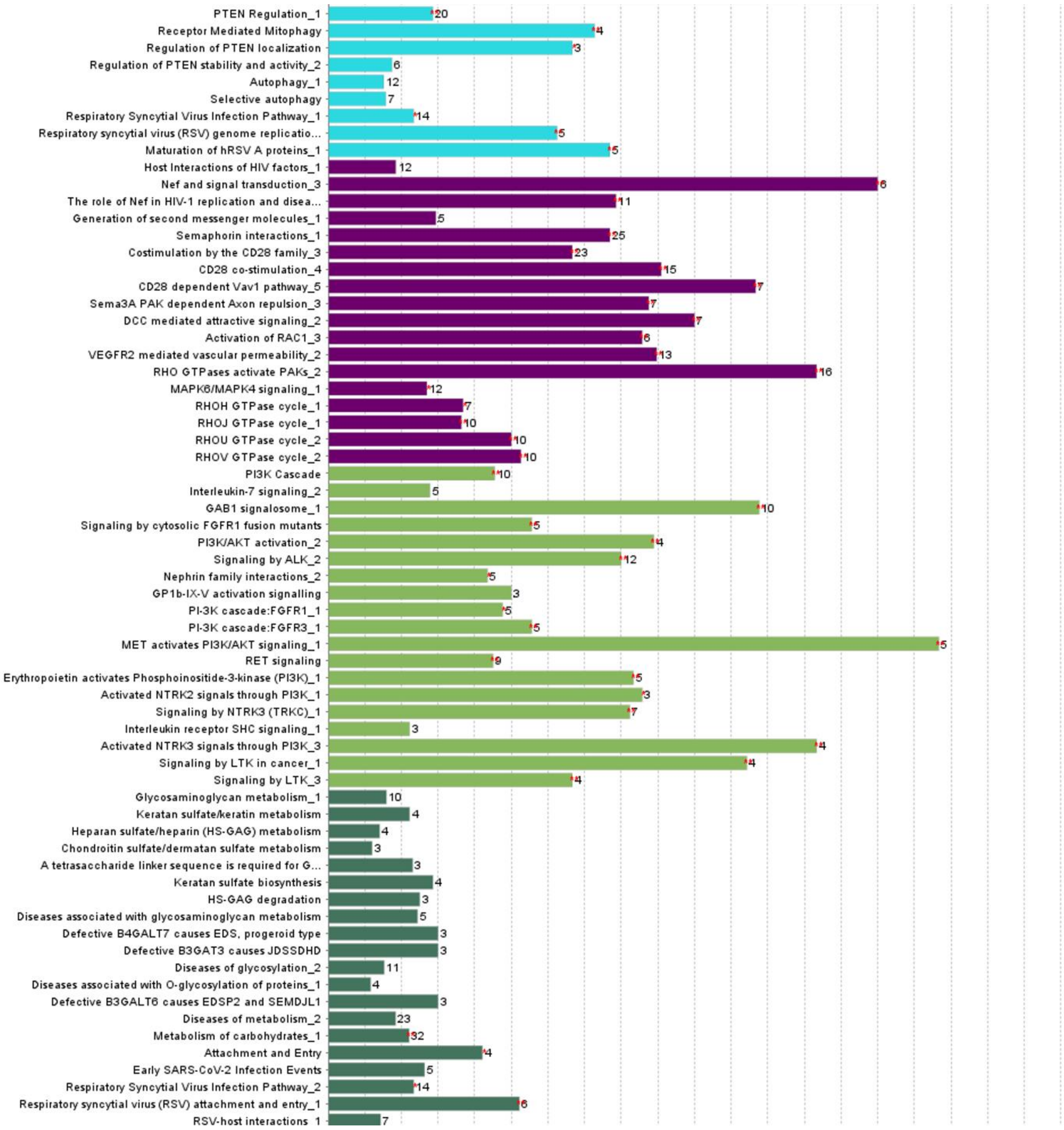
PTEN Regulation_1
Receptor Mediated Mitophagy
Regulation of PTEN localization
Regulation of PTEN stability and activity_2
Autophagy_1
Selective autophagy
Respiratory Syncytial Virus Infection Pathway_1
Respiratory syncytial virus (RSV) genome replicatio...
Maturation of hRSV A proteins_1
Host Interactions of HIV factors_1
Nef and signal transduction_3
The role of Nef in HIV-1 replication and disea...
Generation of second messenger molecules_1
Semaphorin interactions_1
Costimulation by the CD28 family_3
CD28 co-stimulation_4
CD28 dependent Vav1 pathway_5
Sema3A PAK dependent Axon repulsion_3
DCC mediated attractive signaling_2
Activation of RAC1_3
VEGFR2 mediated vascular permeability_2
RHO GTPases activate PAKs_2
MAPK6/MAPK4 signaling_1
RHOH GTPase cycle_1
RHOJ GTPase cycle_1
RHOU GTPase cycle_2
RHOV GTPase cycle_2
PI3K Cascade
Interleukin-7 signaling_2
GAB1 signalosome_1
Signaling by cytosolic FGFR1 fusion mutants
PI3K/AKT activation_2
Signaling by ALK_2
Nephrin family interactions_2
GP1b-IX-V activation signalling
PI-3K cascade:FGFR1_1
PI-3K cascade:FGFR3_1
MET activates PI3K/AKT signaling_1
RET signaling
Erythropoietin activates Phosphoinositide-3-kinase (PI3K)_1
Activated NTRK2 signals through PI3K_1
Signaling by NTRK3 (TRKC)_1
Interleukin receptor SHC signaling_1
Activated NTRK3 signals through PI3K_3
Signaling by LTK in cancer_1
Signaling by LTK_3
Glycosaminoglycan metabolism_1
Keratan sulfate/keratin metabolism
Heparan sulfate/heparin (HS-GAG) metabolism
Chondroitin sulfate/dermatan sulfate metabolism
A tetrasaccharide linker sequence is required for G...
Keratan sulfate biosynthesis
HS-GAG degradation
Diseases associated with glycosaminoglycan metabolism
Defective B4GALT7 causes EDS, progeroid type
Defective B3GAT3 causes JDSSDHD
Diseases of glycosylation_2
Diseases associated with O-glycosylation of proteins_1
Defective B3GALT6 causes EDSP2 and SEMDJL1
Diseases of metabolism_2
Metabolism of carbohydrates_1
Attachment and Entry
Early SARS-CoV-2 Infection Events
Respiratory Syncytial Virus Infection Pathway_2
Respiratory syncytial virus (RSV) attachment and entry_1
RSV-host interactions_1
20
4
3
6
12
7
14
5
5
12
6
11
5
25
23
15
7
7
7
6
13
16
12
7
10
10
10
10
5
10
5
4
12
5
3
5
5
5
9
5
3
7
3
4
4
4
10
4
4
3
3
4
3
5
3
3
11
4
3
23
32
4
5
14
6
7

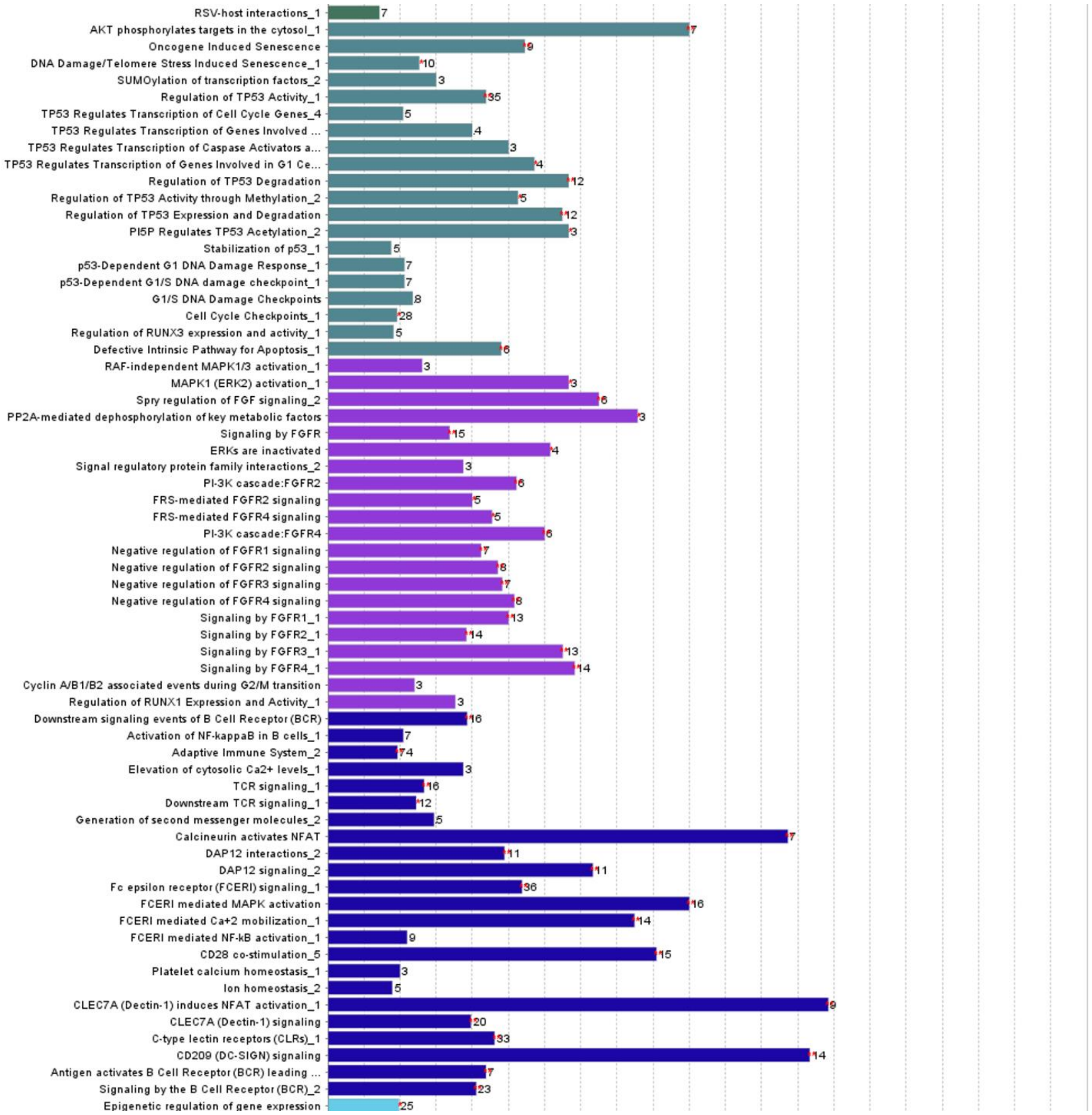
RSV-host interactions_1 7
AKT phosphorylates targets in the cytosol_1 7
Oncogene Induced Senescence 9
DNA Damage/Telomere Stress Induced Senescence_1 10
SUMOylation of transcription factors_2 3
Regulation of TP53 Activity_1 35
TP53 Regulates Transcription of Cell Cycle Genes_4 5
TP53 Regulates Transcription of Genes Involved ... 4
TP53 Regulates Transcription of Caspase Activators a... 3
TP53 Regulates Transcription of Genes Involved in G1 Ce... 4
Regulation of TP53 Degradation 12
Regulation of TP53 Activity through Methylation_2 5
Regulation of TP53 Expression and Degradation 12
PI5P Regulates TP53 Acetylation_2 3
Stabilization of p53_1 5
p53-Dependent G1 DNA Damage Response_1 7
p53-Dependent G1/S DNA damage checkpoint_1 7
G1/S DNA Damage Checkpoints 8
Cell Cycle Checkpoints_1 28
Regulation of RUNX3 expression and activity_1 5
Defective Intrinsic Pathway for Apoptosis_1 6
RAF-independent MAPK1/3 activation_1 3
MAPK1 (ERK2) activation_1 3
Spry regulation of FGF signaling_2 6
PP2A-mediated dephosphorylation of key metabolic factors 3
Signaling by FGFR 15
ERKs are inactivated 4
Signal regulatory protein family interactions_2 3
PI-3K cascade:FGFR2 6
FRS-mediated FGFR2 signaling 5
FRS-mediated FGFR4 signaling 5
PI-3K cascade:FGFR4 6
Negative regulation of FGFR1 signaling 7
Negative regulation of FGFR2 signaling 8
Negative regulation of FGFR3 signaling 7
Negative regulation of FGFR4 signaling 8
Signaling by FGFR1_1 13
Signaling by FGFR2_1 14
Signaling by FGFR3_1 13
Signaling by FGFR4_1 14
Cyclin A/B1/B2 associated events during G2/M transition 3
Regulation of RUNX1 Expression and Activity_1 3
Downstream signaling events of B Cell Receptor (BCR) 16
Activation of NF-kappaB in B cells_1 7
Adaptive Immune System_2 74
Elevation of cytosolic Ca2+ levels_1 3
TCR signaling_1 16
Downstream TCR signaling_1 12
Generation of second messenger molecules_2 5
Calcineurin activates NFAT 7
DAP12 interactions_2 11
DAP12 signaling_2 11
Fc epsilon receptor (FCERI) signaling_1 36
FCERI mediated MAPK activation 16
FCERI mediated Ca+2 mobilization_1 14
FCERI mediated NF-kB activation_1 9
CD28 co-stimulation_5 15
Platelet calcium homeostasis_1 3
Ion homeostasis_2 5
CLEC7A (Dectin-1) induces NFAT activation_1 9
CLEC7A (Dectin-1) signaling 20
C-type lectin receptors (CLRs)_1 33
CD209 (DC-SIGN) signaling 14
Antigen activates B Cell Receptor (BCR) leading ... 7
Signaling by the B Cell Receptor (BCR)_2 23
Epigenetic regulation of gene expression 25

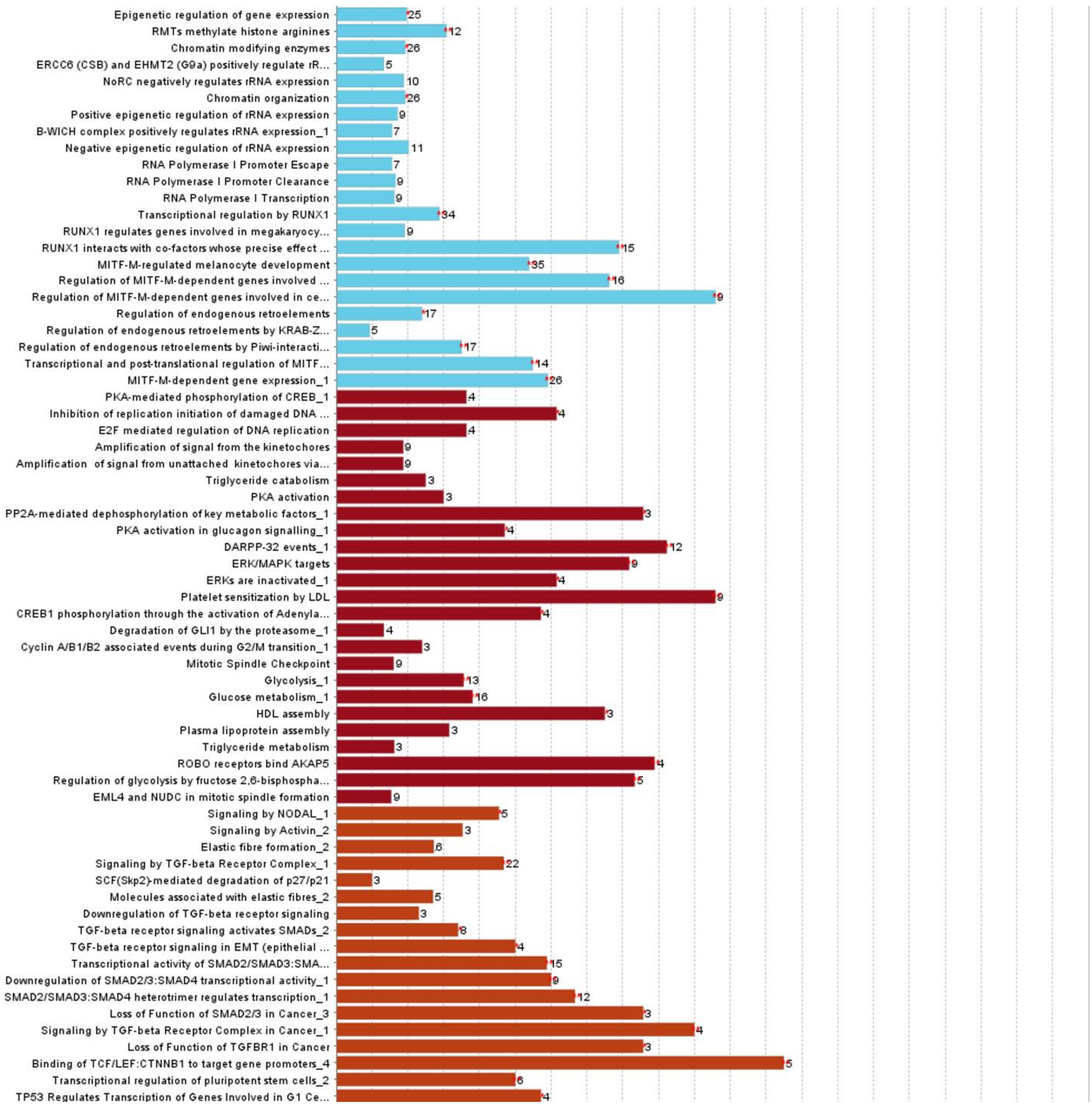

Epigenetic regulation of gene expression
RMTs methylate histone arginines
Chromatin modifying enzymes
ERCC6 (CSB) and EHMT2 (G9a) positively regulate rR...
NoRC negatively regulates rRNA expression
Chromatin organization
Positive epigenetic regulation of rRNA expression
B-WICH complex positively regulates rRNA expression_1
Negative epigenetic regulation of rRNA expression
RNA Polymerase I Promoter Escape
RNA Polymerase I Promoter Clearance
RNA Polymerase I Transcription
Transcriptional regulation by RUNX1
RUNX1 regulates genes involved in megakaryocy...
RUNX1 interacts with co-factors whose precise effect ...
MITF-M-regulated melanocyte development
Regulation of MITF-M-dependent genes involved ...
Regulation of MITF-M-dependent genes involved in ce...
Regulation of endogenous retroelements
Regulation of endogenous retroelements by KRAB-Z...
Regulation of endogenous retroelements by Piwi-interacti...
Transcriptional and post-translational regulation of MITF...
MITF-M-dependent gene expression_1
PKA-mediated phosphorylation of CREB_1
Inhibition of replication initiation of damaged DNA ...
E2F mediated regulation of DNA replication
Amplification of signal from the kinetochores
Amplification of signal from unattached kinetochores via...
Triglyceride catabolism
PKA activation
PP2A-mediated dephosphorylation of key metabolic factors_1
PKA activation in glucagon signalling_1
DARPP-32 events_1
ERK/MAPK targets
ERKs are inactivated_1
Platelet sensitization by LDL
CREB1 phosphorylation through the activation of Adenyla...
Degradation of GLI1 by the proteasome_1
Cyclin A/B1/B2 associated events during G2/M transition_1
Mitotic Spindle Checkpoint
Glycolysis_1
Glucose metabolism_1
HDL assembly
Plasma lipoprotein assembly
Triglyceride metabolism
ROBO receptors bind AKAP5
Regulation of glycolysis by fructose 2,6-bisphospha...
EML4 and NUDC in mitotic spindle formation
Signaling by NODAL_1
Signaling by Activin_2
Elastic fibre formation_2
Signaling by TGF-beta Receptor Complex_1
SCF(Skp2)-mediated degradation of p27/p21
Molecules associated with elastic fibres_2
Downregulation of TGF-beta receptor signaling
TGF-beta receptor signaling activates SMADs_2
TGF-beta receptor signaling in EMT (epithelial ...
Transcriptional activity of SMAD2/SMAD3:SMA...
Downregulation of SMAD2/3:SMAD4 transcriptional activity_1
SMAD2/SMAD3:SMAD4 heterotrimer regulates transcription_1
Loss of Function of SMAD2/3 in Cancer_3
Signaling by TGF-beta Receptor Complex in Cancer_1
Loss of Function of TGFBR1 in Cancer
Binding of TCF/LEF:CTNNB1 to target gene promoters_4
Transcriptional regulation of pluripotent stem cells_2
TP53 Regulates Transcription of Genes Involved in G1 Ce...
25
12
26
5
10
26
9
7
11
7
9
9
34
9
15
35
16
9
17
5
17
14
26
4
4
4
9
9
3
3
3
4
12
9
4
9
4
4
3
9
13
16
3
3
3
4
5
9
5
3
6
22
3
5
3
8
4
15
9
12
3
4
3
5
6
4

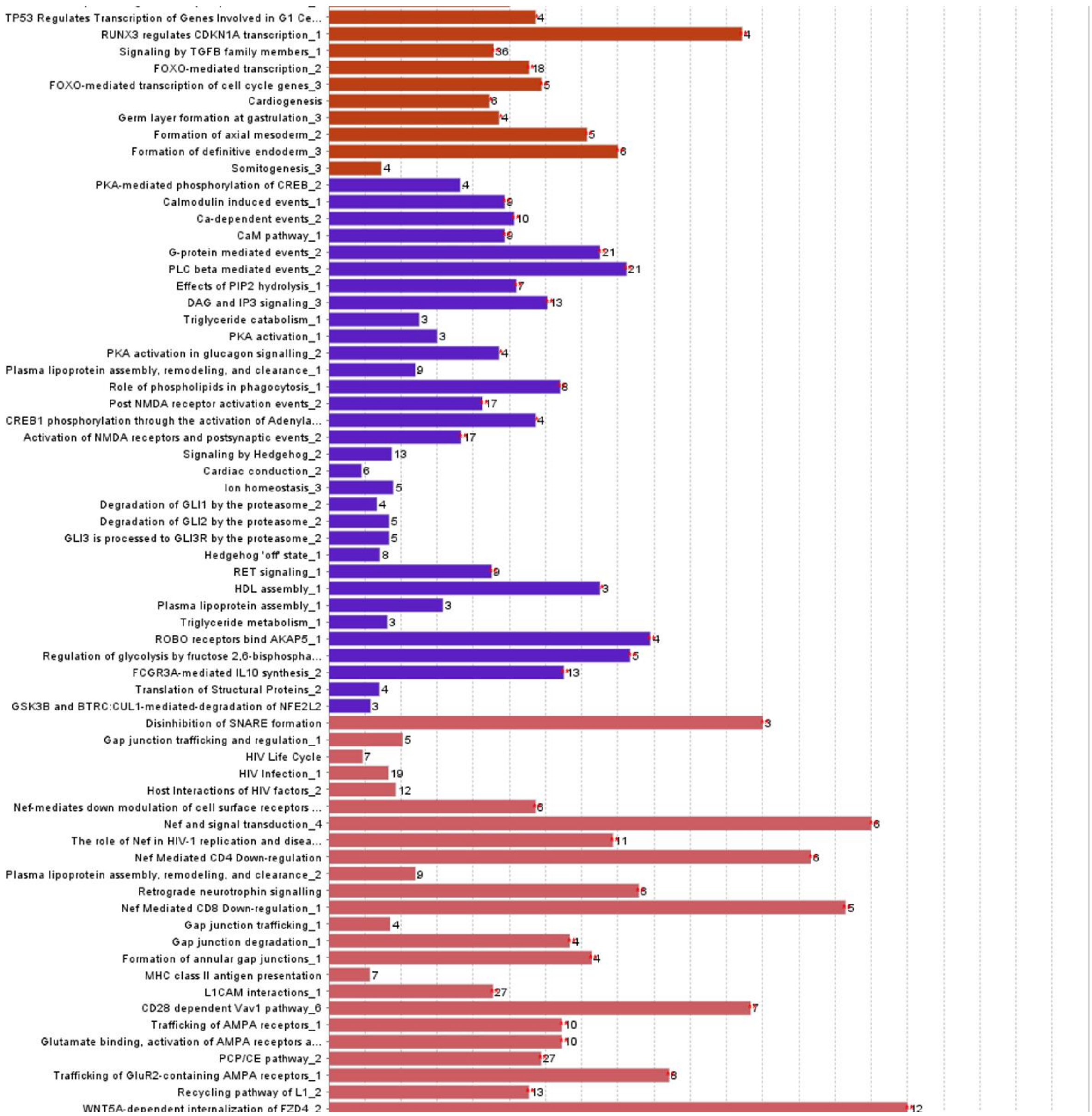
TP53 Regulates Transcription of Genes Involved in G1 Ce...
4
RUNX3 regulates CDKN1A transcription_1
4
Signaling by TGFB family members_1
36
FOXO-mediated transcription_2
18
FOXO-mediated transcription of cell cycle genes_3
5
Cardiogenesis
6
Germ layer formation at gastrulation_3
4
Formation of axial mesoderm_2
5
Formation of definitive endoderm_3
6
Somitogenesis_3
4
PKA-mediated phosphorylation of CREB_2
4
Calmodulin induced events_1
9
Ca-dependent events_2
10
CaM pathway_1
9
G-protein mediated events_2
21
PLC beta mediated events_2
21
Effects of PIP2 hydrolysis_1
7
DAG and IP3 signaling_3
13
Triglyceride catabolism_1
3
PKA activation_1
3
PKA activation in glucagon signalling_2
4
Plasma lipoprotein assembly, remodeling, and clearance_1
9
Role of phospholipids in phagocytosis_1
8
Post NMDA receptor activation events_2
17
CREB1 phosphorylation through the activation of Adenyla...
4
Activation of NMDA receptors and postsynaptic events_2
17
Signaling by Hedgehog_2
13
Cardiac conduction_2
6
Ion homeostasis_3
5
Degradation of GLI1 by the proteasome_2
4
Degradation of GLI2 by the proteasome_2
5
GLI3 is processed to GLI3R by the proteasome_2
5
Hedgehog 'off' state_1
8
RET signaling_1
9
HDL assembly_1
3
Plasma lipoprotein assembly_1
3
Triglyceride metabolism_1
3
ROBO receptors bind AKAP5_1
4
Regulation of glycolysis by fructose 2,6-bisphospha...
5
FCGR3A-mediated IL10 synthesis_2
13
Translation of Structural Proteins_2
4
GSK3B and BTRC:CUL1-mediated-degradation of NFE2L2
3
Disinhibition of SNARE formation
3
Gap junction trafficking and regulation_1
5
HIV Life Cycle
7
HIV Infection_1
19
Host Interactions of HIV factors_2
12
Nef-mediates down modulation of cell surface receptors ...
6
Nef and signal transduction_4
6
The role of Nef in HIV-1 replication and disea...
11
Nef Mediated CD4 Down-regulation
6
Plasma lipoprotein assembly, remodeling, and clearance_2
9
Retrograde neurotrophin signalling
6
Nef Mediated CD8 Down-regulation_1
5
Gap junction trafficking_1
4
Gap junction degradation_1
4
Formation of annular gap junctions_1
4
MHC class II antigen presentation
7
L1CAM interactions_1
27
CD28 dependent Vav1 pathway_6
7
Trafficking of AMPA receptors_1
10
Glutamate binding, activation of AMPA receptors a...
10
PCP/CE pathway_2
27
Trafficking of GluR2-containing AMPA receptors_1
8
Recycling pathway of L1_2
13
WNT5A-dependent internalization of FZD4_2
12

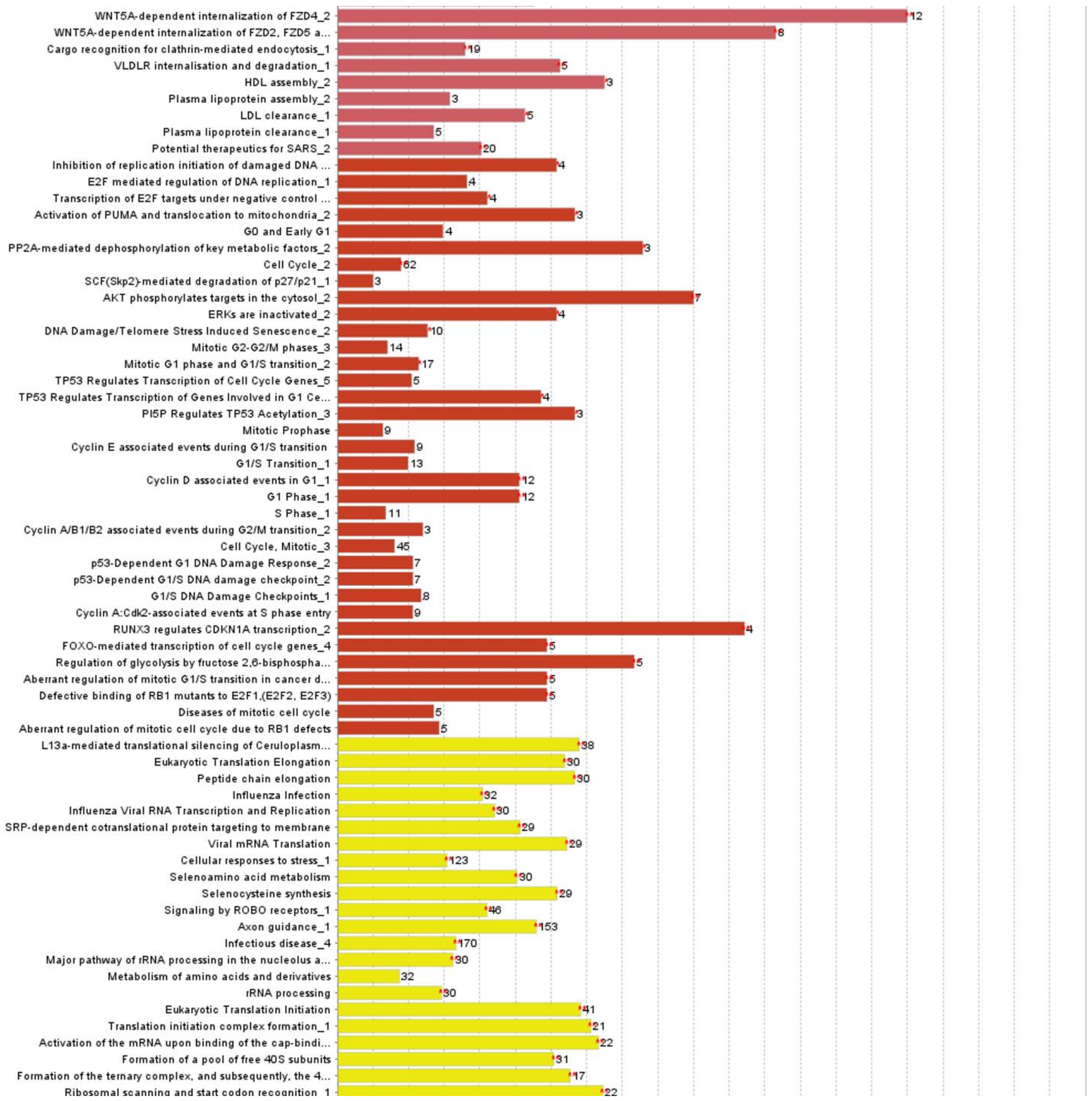
WNT5A-dependent internalization of FZD4_2
12
WNT5A-dependent internalization of FZD2, FZD5 a...
8
Cargo recognition for clathrin-mediated endocytosis_1
19
VLDLR internalisation and degradation_1
5
HDL assembly_2
3
Plasma lipoprotein assembly_2
3
LDL clearance_1
5
Plasma lipoprotein clearance_1
5
Potential therapeutics for SARS_2
20
Inhibition of replication initiation of damaged DNA ...
4
E2F mediated regulation of DNA replication_1
4
Transcription of E2F targets under negative control ...
4
Activation of PUMA and translocation to mitochondria_2
3
G0 and Early G1
4
PP2A-mediated dephosphorylation of key metabolic factors_2
3
Cell Cycle_2
62
SCF(Skp2)-mediated degradation of p27/p21_1
3
AKT phosphorylates targets in the cytosol_2
7
ERKs are inactivated_2
4
DNA Damage/Telomere Stress Induced Senescence_2
10
Mitotic G2-G2/M phases_3
14
Mitotic G1 phase and G1/S transition_2
17
TP53 Regulates Transcription of Cell Cycle Genes_5
5
TP53 Regulates Transcription of Genes Involved in G1 Ce...
4
PI5P Regulates TP53 Acetylation_3
3
Mitotic Prophase
9
Cyclin E associated events during G1/S transition
9
G1/S Transition_1
13
Cyclin D associated events in G1_1
12
G1 Phase_1
12
S Phase_1
11
Cyclin A/B1/B2 associated events during G2/M transition_2
3
Cell Cycle, Mitotic_3
45
p53-Dependent G1 DNA Damage Response_2
7
p53-Dependent G1/S DNA damage checkpoint_2
7
G1/S DNA Damage Checkpoints_1
8
Cyclin A:Cdk2-associated events at S phase entry
9
RUNX3 regulates CDKN1A transcription_2
4
FOXO-mediated transcription of cell cycle genes_4
5
Regulation of glycolysis by fructose 2,6-bisphospha...
5
Aberrant regulation of mitotic G1/S transition in cancer d...
5
Defective binding of RB1 mutants to E2F1,(E2F2, E2F3)
5
Diseases of mitotic cell cycle
5
Aberrant regulation of mitotic cell cycle due to RB1 defects
5
L13a-mediated translational silencing of Ceruloplasm...
38
Eukaryotic Translation Elongation
30
Peptide chain elongation
30
Influenza Infection
32
Influenza Viral RNA Transcription and Replication
30
SRP-dependent cotranslational protein targeting to membrane
29
Viral mRNA Translation
29
Cellular responses to stress_1
123
Selenoamino acid metabolism
30
Selenocysteine synthesis
29
Signaling by ROBO receptors_1
46
Axon guidance_1
153
Infectious disease_4
170
Major pathway of rRNA processing in the nucleolus a...
30
Metabolism of amino acids and derivatives
32
rRNA processing
30
Eukaryotic Translation Initiation
41
Translation initiation complex formation_1
21
Activation of the mRNA upon binding of the cap-bindi...
22
Formation of a pool of free 40S subunits
31
Formation of the ternary complex, and subsequently, the 4...
17
Ribosomal scanning and start codon recognition_1
22

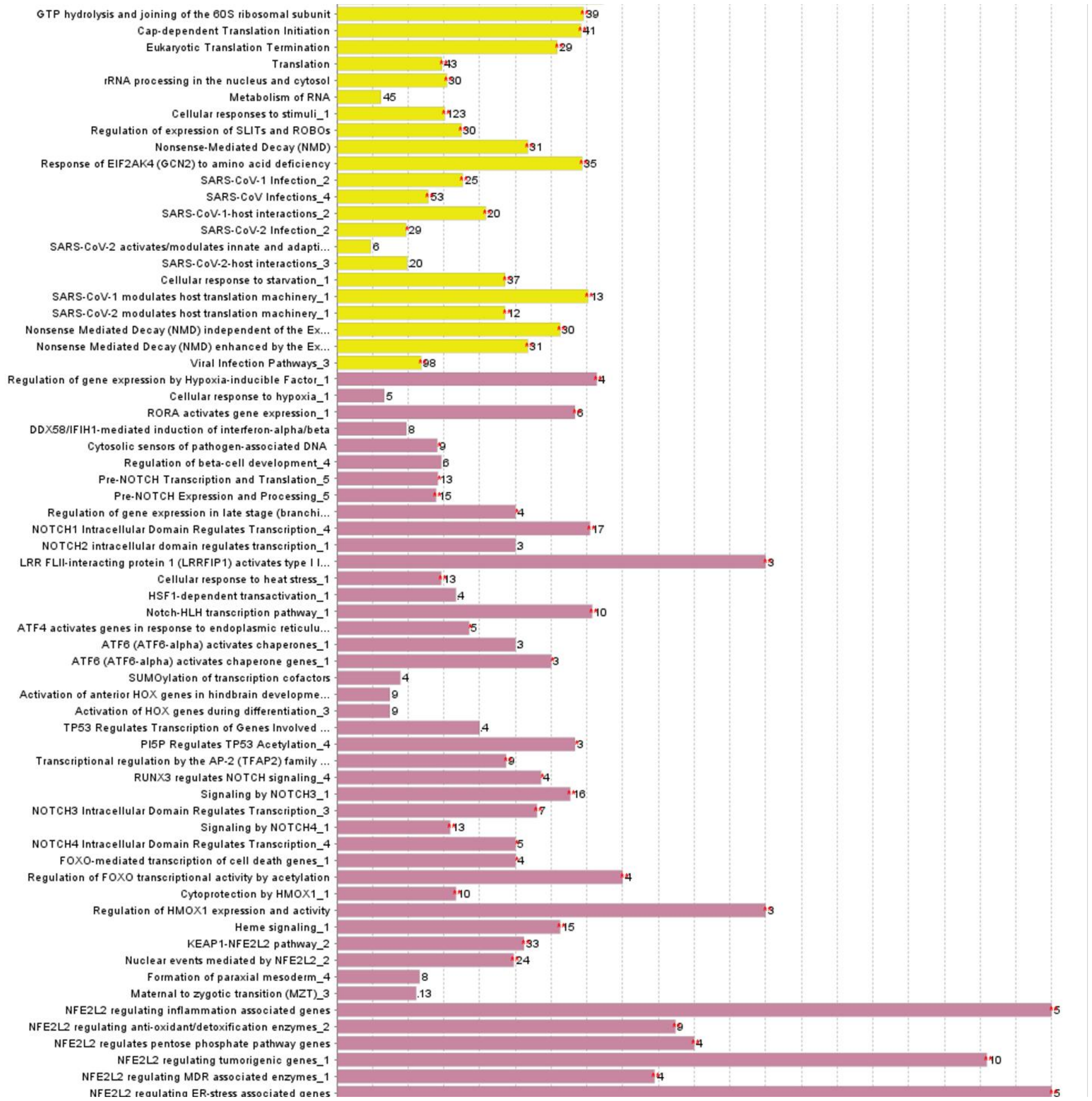
GTP hydrolysis and joining of the 60S ribosomal subunit 39
Cap-dependent Translation Initiation 41
Eukaryotic Translation Termination 29
Translation 43
rRNA processing in the nucleus and cytosol 30
Metabolism of RNA 45
Cellular responses to stimuli_1 123
Regulation of expression of SLITs and ROBOs 30
Nonsense-Mediated Decay (NMD) 31
Response of EIF2AK4 (GCN2) to amino acid deficiency 35
SARS-CoV-1 Infection_2 25
SARS-CoV Infections_4 53
SARS-CoV-1-host interactions_2 20
SARS-CoV-2 Infection_2 29
SARS-CoV-2 activates/modulates innate and adapti... 6
SARS-CoV-2-host interactions_3 20
Cellular response to starvation_1 37
SARS-CoV-1 modulates host translation machinery_1 13
SARS-CoV-2 modulates host translation machinery_1 12
Nonsense Mediated Decay (NMD) independent of the Ex... 30
Nonsense Mediated Decay (NMD) enhanced by the Ex... 31
Viral Infection Pathways_3 98
Regulation of gene expression by Hypoxia-inducible Factor_1 4
Cellular response to hypoxia_1 5
RORA activates gene expression_1 6
DDX58/IFIH1-mediated induction of interferon-alpha/beta 8
Cytosolic sensors of pathogen-associated DNA 9
Regulation of beta-cell development_4 6
Pre-NOTCH Transcription and Translation_5 13
Pre-NOTCH Expression and Processing_5 15
Regulation of gene expression in late stage (branchi... 4
NOTCH1 Intracellular Domain Regulates Transcription_4 17
NOTCH2 intracellular domain regulates transcription_1 3
LRR FLII-interacting protein 1 (LRRFIP1) activates type I I... 3
Cellular response to heat stress_1 13
HSF1-dependent transactivation_1 4
Notch-HLH transcription pathway_1 10
ATF4 activates genes in response to endoplasmic reticulu... 5
ATF6 (ATF6-alpha) activates chaperones_1 3
ATF6 (ATF6-alpha) activates chaperone genes_1 3
SUMOylation of transcription cofactors 4
Activation of anterior HOX genes in hindbrain developme... 9
Activation of HOX genes during differentiation_3 9
TP53 Regulates Transcription of Genes Involved ... 4
PI5P Regulates TP53 Acetylation_4 3
Transcriptional regulation by the AP-2 (TFAP2) family ... 9
RUNX3 regulates NOTCH signaling_4 4
Signaling by NOTCH3_1 16
NOTCH3 Intracellular Domain Regulates Transcription_3 7
Signaling by NOTCH4_1 13
NOTCH4 Intracellular Domain Regulates Transcription_4 5
FOXO-mediated transcription of cell death genes_1 4
Regulation of FOXO transcriptional activity by acetylation 4
Cytoprotection by HMOX1_1 10
Regulation of HMOX1 expression and activity 3
Heme signaling_1 15
KEAP1-NFE2L2 pathway_2 33
Nuclear events mediated by NFE2L2_2 24
Formation of paraxial mesoderm_4 8
Maternal to zygotic transition (MZT)_3 13
NFE2L2 regulating inflammation associated genes 5
NFE2L2 regulating anti-oxidant/detoxification enzymes_2 9
NFE2L2 regulates pentose phosphate pathway genes 4
NFE2L2 regulating tumorigenic genes_1 10
NFE2L2 regulating MDR associated enzymes_1 4
NFE2L2 regulating ER-stress associated genes 5

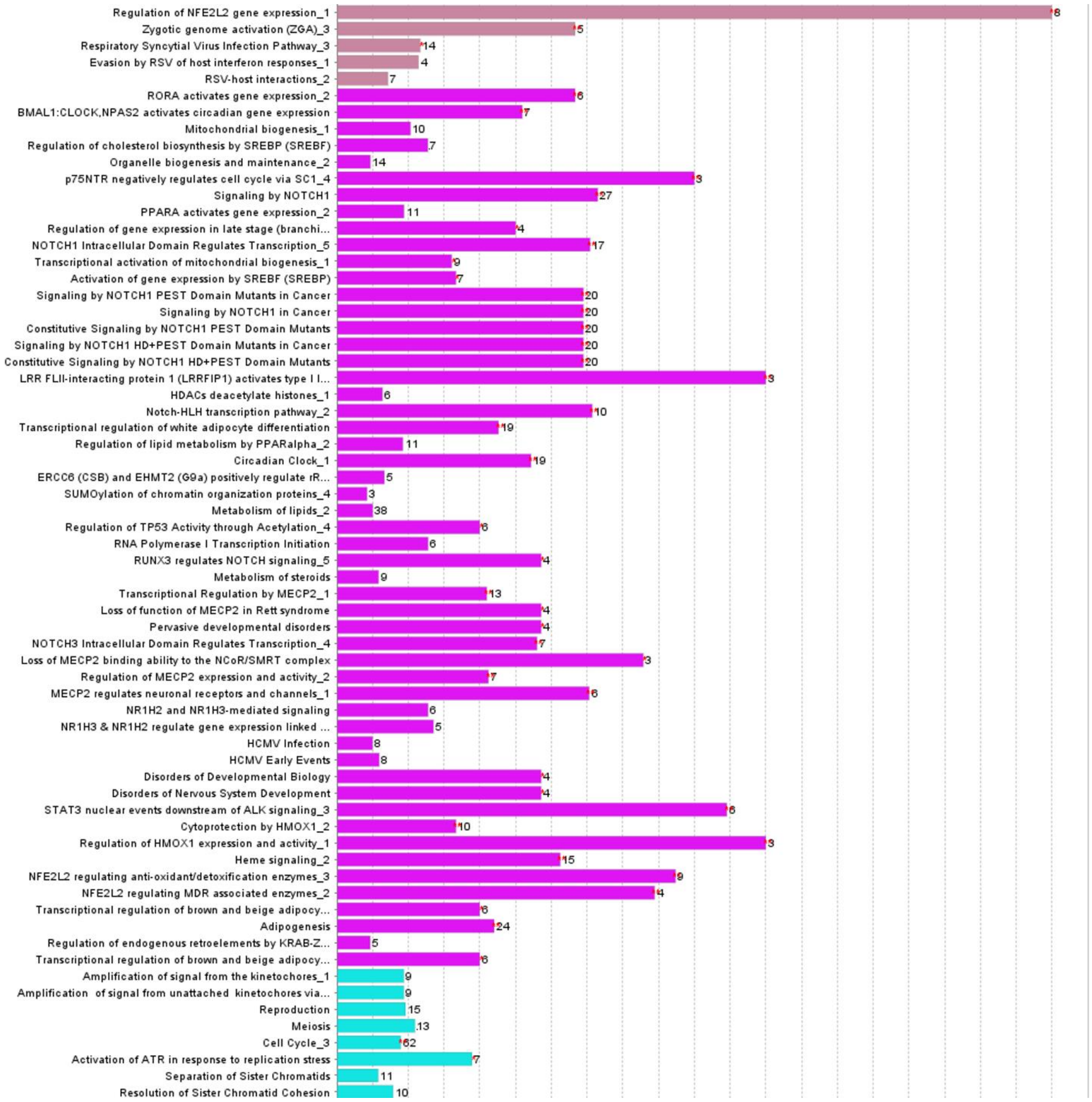
Regulation of NFE2L2 gene expression_1 8
Zygotic genome activation (ZGA)_3 5
Respiratory Syncytial Virus Infection Pathway_3 14
Evasion by RSV of host interferon responses_1 4
RSV-host interactions_2 7
RORA activates gene expression_2 6
BMAL1:CLOCK,NPAS2 activates circadian gene expression 7
Mitochondrial biogenesis_1 10
Regulation of cholesterol biosynthesis by SREBP (SREBF) 7
Organelle biogenesis and maintenance_2 14
p75NTR negatively regulates cell cycle via SC1_4 3
Signaling by NOTCH1 27
PPARA activates gene expression_2 11
Regulation of gene expression in late stage (branchi... 4
NOTCH1 Intracellular Domain Regulates Transcription_5 17
Transcriptional activation of mitochondrial biogenesis_1 9
Activation of gene expression by SREBF (SREBP) 7
Signaling by NOTCH1 PEST Domain Mutants in Cancer 20
Signaling by NOTCH1 in Cancer 20
Constitutive Signaling by NOTCH1 PEST Domain Mutants 20
Signaling by NOTCH1 HD+PEST Domain Mutants in Cancer 20
Constitutive Signaling by NOTCH1 HD+PEST Domain Mutants 20
LRR FLII-interacting protein 1 (LRRFIP1) activates type I I... 3
HDACs deacetylate histones_1 6
Notch-HLH transcription pathway_2 10
Transcriptional regulation of white adipocyte differentiation 19
Regulation of lipid metabolism by PPARalpha_2 11
Circadian Clock_1 19
ERCC6 (CSB) and EHMT2 (G9a) positively regulate rR... 5
SUMOylation of chromatin organization proteins_4 3
Metabolism of lipids_2 38
Regulation of TP53 Activity through Acetylation_4 6
RNA Polymerase I Transcription Initiation 6
RUNX3 regulates NOTCH signaling_5 4
Metabolism of steroids 9
Transcriptional Regulation by MECP2_1 13
Loss of function of MECP2 in Rett syndrome 4
Pervasive developmental disorders 4
NOTCH3 Intracellular Domain Regulates Transcription_4 7
Loss of MECP2 binding ability to the NCoR/SMRT complex 3
Regulation of MECP2 expression and activity_2 7
MECP2 regulates neuronal receptors and channels_1 6
NR1H2 and NR1H3-mediated signaling 6
NR1H3 & NR1H2 regulate gene expression linked ... 5
HCMV Infection 8
HCMV Early Events 8
Disorders of Developmental Biology 4
Disorders of Nervous System Development 4
STAT3 nuclear events downstream of ALK signaling_3 6
Cytoprotection by HMOX1_2 10
Regulation of HMOX1 expression and activity_1 3
Heme signaling_2 15
NFE2L2 regulating anti-oxidant/detoxification enzymes_3 9
NFE2L2 regulating MDR associated enzymes_2 4
Transcriptional regulation of brown and beige adipocy... 6
Adipogenesis 24
Regulation of endogenous retroelements by KRAB-Z... 5
Transcriptional regulation of brown and beige adipocy... 6
Amplification of signal from the kinetochores_1 9
Amplification of signal from unattached kinetochores via... 9
Reproduction 15
Meiosis 13
Cell Cycle_3 62
Activation of ATR in response to replication stress 7
Separation of Sister Chromatids 11
Resolution of Sister Chromatid Cohesion 10

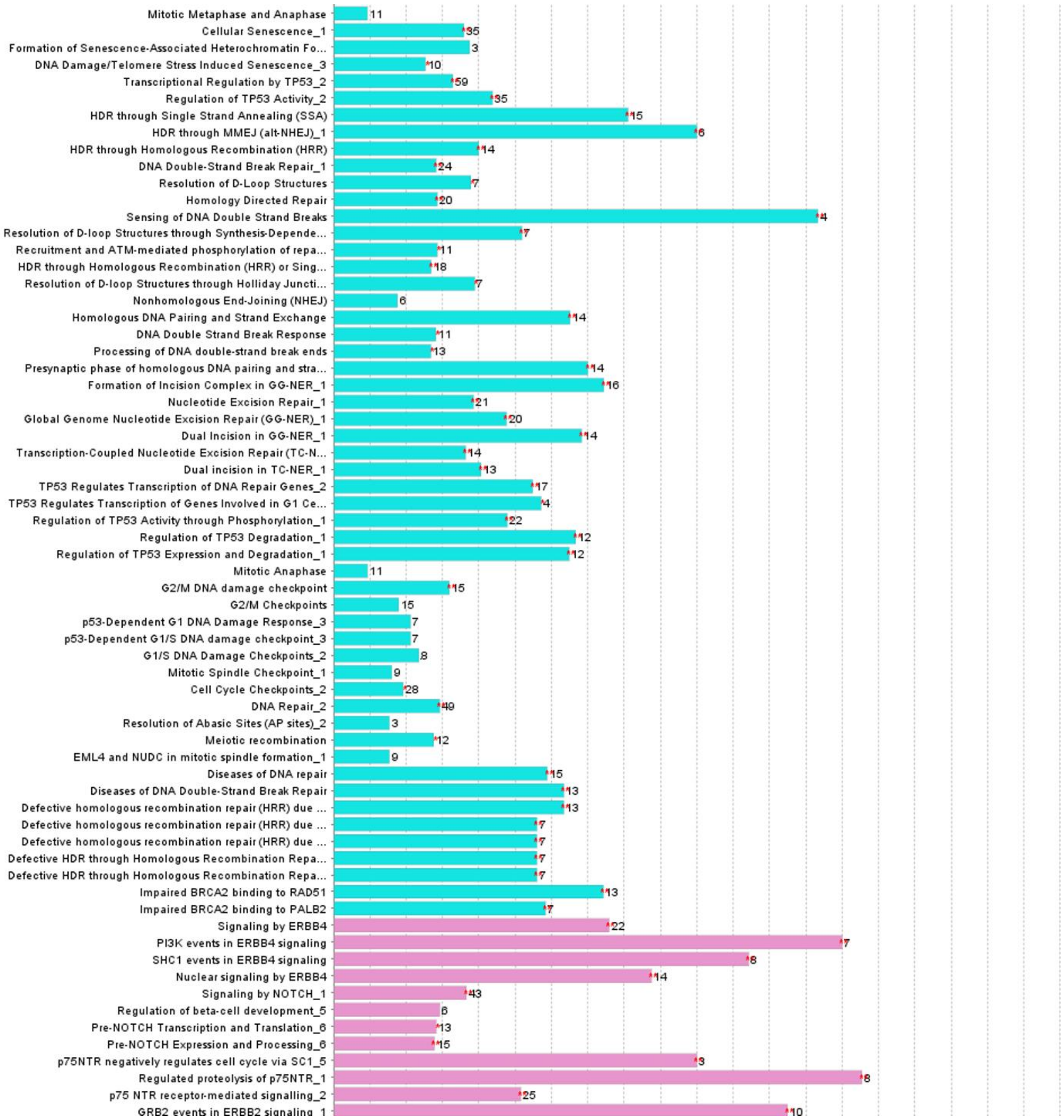
Mitotic Metaphase and Anaphase
Cellular Senescence_1
Formation of Senescence-Associated Heterochromatin Fo...
DNA Damage/Telomere Stress Induced Senescence_3
Transcriptional Regulation by TP53_2
Regulation of TP53 Activity_2
HDR through Single Strand Annealing (SSA)
HDR through MMEJ (alt-NHEJ)_1
HDR through Homologous Recombination (HRR)
DNA Double-Strand Break Repair_1
Resolution of D-Loop Structures
Homology Directed Repair
Sensing of DNA Double Strand Breaks
Resolution of D-loop Structures through Synthesis-Depende...
Recruitment and ATM-mediated phosphorylation of repa...
HDR through Homologous Recombination (HRR) or Sing...
Resolution of D-loop Structures through Holliday Juncti...
Nonhomologous End-Joining (NHEJ)
Homologous DNA Pairing and Strand Exchange
DNA Double Strand Break Response
Processing of DNA double-strand break ends
Presynaptic phase of homologous DNA pairing and stra...
Formation of Incision Complex in GG-NER_1
Nucleotide Excision Repair_1
Global Genome Nucleotide Excision Repair (GG-NER)_1
Dual Incision in GG-NER_1
Transcription-Coupled Nucleotide Excision Repair (TC-N...
Dual incision in TC-NER_1
TP53 Regulates Transcription of DNA Repair Genes_2
TP53 Regulates Transcription of Genes Involved in G1 Ce...
Regulation of TP53 Activity through Phosphorylation_1
Regulation of TP53 Degradation_1
Regulation of TP53 Expression and Degradation_1
Mitotic Anaphase
G2/M DNA damage checkpoint
G2/M Checkpoints
p53-Dependent G1 DNA Damage Response_3
p53-Dependent G1/S DNA damage checkpoint_3
G1/S DNA Damage Checkpoints_2
Mitotic Spindle Checkpoint_1
Cell Cycle Checkpoints_2
DNA Repair_2
Resolution of Abasic Sites (AP sites)_2
Meiotic recombination
EML4 and NUDC in mitotic spindle formation_1
Diseases of DNA repair
Diseases of DNA Double-Strand Break Repair
Defective homologous recombination repair (HRR) due ...
Defective homologous recombination repair (HRR) due ...
Defective homologous recombination repair (HRR) due ...
Defective HDR through Homologous Recombination Repa...
Defective HDR through Homologous Recombination Repa...
Impaired BRCA2 binding to RAD51
Impaired BRCA2 binding to PALB2
Signaling by ERBB4
PI3K events in ERBB4 signaling
SHC1 events in ERBB4 signaling
Nuclear signaling by ERBB4
Signaling by NOTCH_1
Regulation of beta-cell development_5
Pre-NOTCH Transcription and Translation_6
Pre-NOTCH Expression and Processing_6
p75NTR negatively regulates cell cycle via SC1_5
Regulated proteolysis of p75NTR_1
p75 NTR receptor-mediated signalling_2
GRB2 events in ERBB2 signaling_1
11
35
3
10
59
35
15
6
14
24
7
20
4
7
11
18
7
6
14
11
13
14
16
21
20
14
14
13
17
4
22
12
12
11
15
15
7
7
8
9
28
49
3
12
9
15
13
13
7
7
7
7
7
13
7
22
7
8
14
43
6
13
15
3
8
25
10

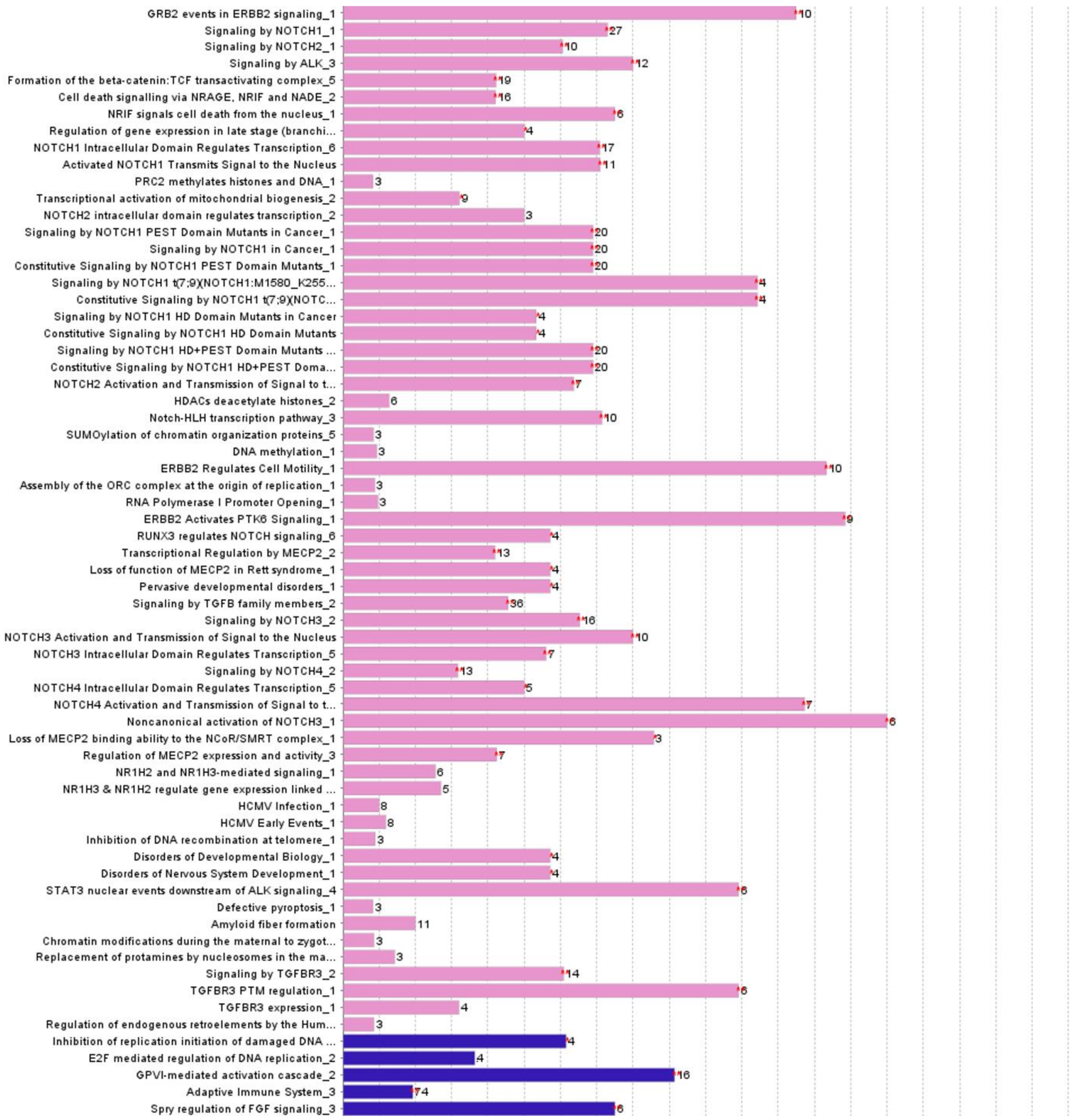
GRB2 events in ERBB2 signaling_1 10
Signaling by NOTCH1_1 27
Signaling by NOTCH2_1 10
Signaling by ALK_3 12
Formation of the beta-catenin:TCF transactivating complex_5 19
Cell death signalling via NRAGE, NRIF and NADE_2 16
NRIF signals cell death from the nucleus_1 6
Regulation of gene expression in late stage (branchi... 4
NOTCH1 Intracellular Domain Regulates Transcription_6 17
Activated NOTCH1 Transmits Signal to the Nucleus 11
PRC2 methylates histones and DNA_1 3
Transcriptional activation of mitochondrial biogenesis_2 9
NOTCH2 intracellular domain regulates transcription_2 3
Signaling by NOTCH1 PEST Domain Mutants in Cancer_1 20
Signaling by NOTCH1 in Cancer_1 20
Constitutive Signaling by NOTCH1 PEST Domain Mutants_1 20
Signaling by NOTCH1 t(7;9)(NOTCH1:M1580_K255... 4
Constitutive Signaling by NOTCH1 t(7;9)(NOTC... 4
Signaling by NOTCH1 HD Domain Mutants in Cancer 4
Constitutive Signaling by NOTCH1 HD Domain Mutants 4
Signaling by NOTCH1 HD+PEST Domain Mutants ... 20
Constitutive Signaling by NOTCH1 HD+PEST Doma... 20
NOTCH2 Activation and Transmission of Signal to t... 7
HDACs deacetylate histones_2 6
Notch-HLH transcription pathway_3 10
SUMOylation of chromatin organization proteins_5 3
DNA methylation_1 3
ERBB2 Regulates Cell Motility_1 10
Assembly of the ORC complex at the origin of replication_1 3
RNA Polymerase I Promoter Opening_1 3
ERBB2 Activates PTK6 Signaling_1 9
RUNX3 regulates NOTCH signaling_6 4
Transcriptional Regulation by MECP2_2 13
Loss of function of MECP2 in Rett syndrome_1 4
Pervasive developmental disorders_1 4
Signaling by TGFB family members_2 36
Signaling by NOTCH3_2 16
NOTCH3 Activation and Transmission of Signal to the Nucleus 10
NOTCH3 Intracellular Domain Regulates Transcription_5 7
Signaling by NOTCH4_2 13
NOTCH4 Intracellular Domain Regulates Transcription_5 5
NOTCH4 Activation and Transmission of Signal to t... 7
Noncanonical activation of NOTCH3_1 6
Loss of MECP2 binding ability to the NCoR/SMRT complex_1 3
Regulation of MECP2 expression and activity_3 7
NR1H2 and NR1H3-mediated signaling_1 6
NR1H3 & NR1H2 regulate gene expression linked ... 5
HCMV Infection_1 8
HCMV Early Events_1 8
Inhibition of DNA recombination at telomere_1 3
Disorders of Developmental Biology_1 4
Disorders of Nervous System Development_1 4
STAT3 nuclear events downstream of ALK signaling_4 6
Defective pyroptosis_1 3
Amyloid fiber formation 11
Chromatin modifications during the maternal to zygot... 3
Replacement of protamines by nucleosomes in the ma... 3
Signaling by TGFBR3_2 14
TGFBR3 PTM regulation_1 6
TGFBR3 expression_1 4
Regulation of endogenous retroelements by the Hum... 3
Inhibition of replication initiation of damaged DNA ... 4
E2F mediated regulation of DNA replication_2 4
GPVI-mediated activation cascade_2 16
Adaptive Immune System_3 74
Spry regulation of FGF signaling_3 6

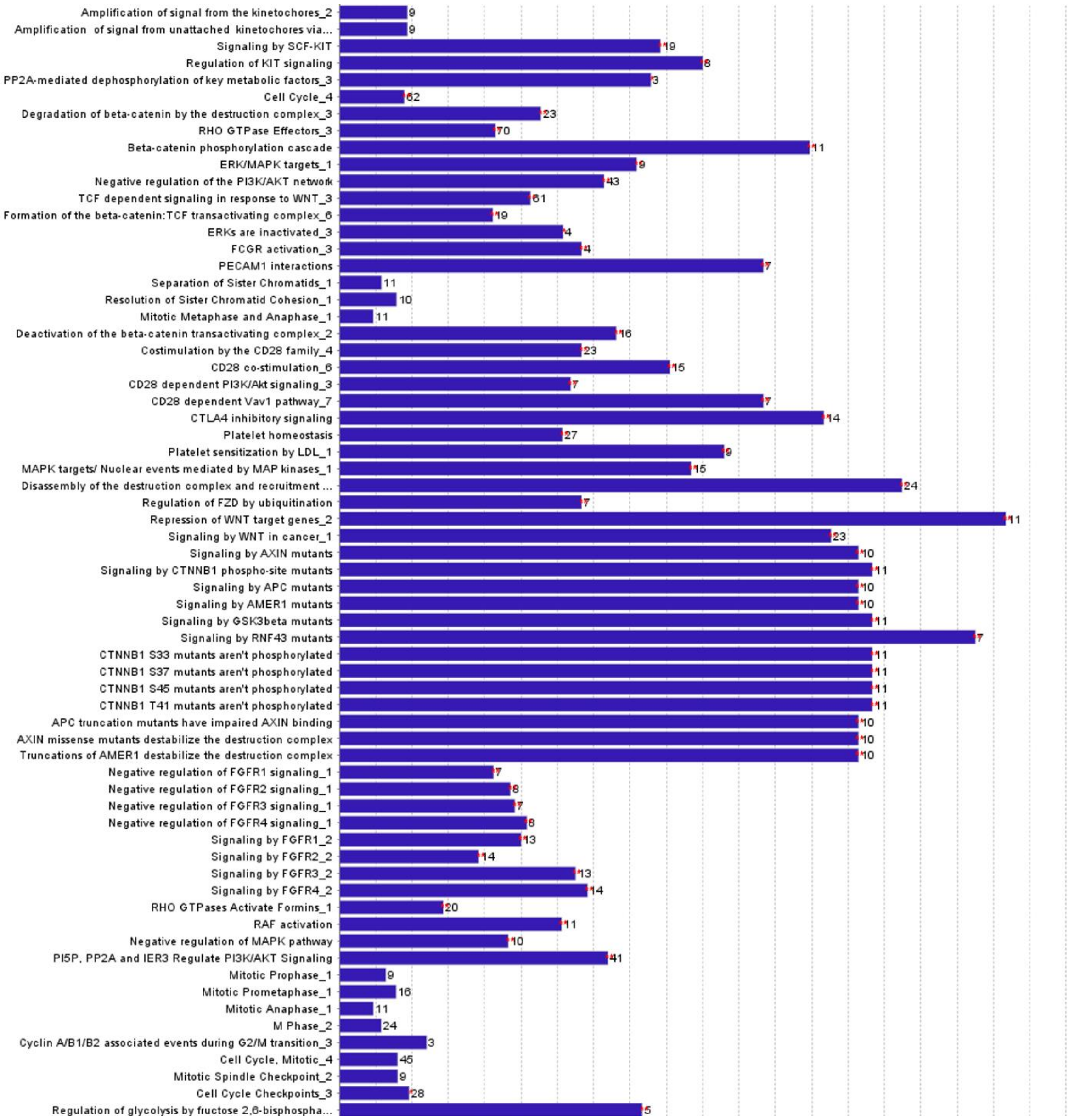
Amplification of signal from the kinetochores_2
9
Amplification of signal from unattached kinetochores via...
9
Signaling by SCF-KIT
19
Regulation of KIT signaling
8
PP2A-mediated dephosphorylation of key metabolic factors_3
3
Cell Cycle_4
62
Degradation of beta-catenin by the destruction complex_3
23
RHO GTPase Effectors_3
70
Beta-catenin phosphorylation cascade
11
ERK/MAPK targets_1
9
Negative regulation of the PI3K/AKT network
43
TCF dependent signaling in response to WNT_3
61
Formation of the beta-catenin:TCF transactivating complex_6
19
ERKs are inactivated_3
4
FCGR activation_3
4
PECAM1 interactions
7
Separation of Sister Chromatids_1
11
Resolution of Sister Chromatid Cohesion_1
10
Mitotic Metaphase and Anaphase_1
11
Deactivation of the beta-catenin transactivating complex_2
16
Costimulation by the CD28 family_4
23
CD28 co-stimulation_6
15
CD28 dependent PI3K/Akt signaling_3
7
CD28 dependent Vav1 pathway_7
7
CTLA4 inhibitory signaling
14
Platelet homeostasis
27
Platelet sensitization by LDL_1
9
MAPK targets/ Nuclear events mediated by MAP kinases_1
15
Disassembly of the destruction complex and recruitment ...
24
Regulation of FZD by ubiquitination
7
Repression of WNT target genes_2
11
Signaling by WNT in cancer_1
23
Signaling by AXIN mutants
10
Signaling by CTNNB1 phospho-site mutants
11
Signaling by APC mutants
10
Signaling by AMER1 mutants
10
Signaling by GSK3beta mutants
11
Signaling by RNF43 mutants
7
CTNNB1 S33 mutants aren't phosphorylated
11
CTNNB1 S37 mutants aren't phosphorylated
11
CTNNB1 S45 mutants aren't phosphorylated
11
CTNNB1 T41 mutants aren't phosphorylated
11
APC truncation mutants have impaired AXIN binding
10
AXIN missense mutants destabilize the destruction complex
10
Truncations of AMER1 destabilize the destruction complex
10
Negative regulation of FGFR1 signaling_1
7
Negative regulation of FGFR2 signaling_1
8
Negative regulation of FGFR3 signaling_1
7
Negative regulation of FGFR4 signaling_1
8
Signaling by FGFR1_2
13
Signaling by FGFR2_2
14
Signaling by FGFR3_2
13
Signaling by FGFR4_2
14
RHO GTPases Activate Formins_1
20
RAF activation
11
Negative regulation of MAPK pathway
10
PI5P, PP2A and IER3 Regulate PI3K/AKT Signaling
41
Mitotic Prophase_1
9
Mitotic Prometaphase_1
16
Mitotic Anaphase_1
11
M Phase_2
24
Cyclin A/B1/B2 associated events during G2/M transition_3
3
Cell Cycle, Mitotic_4
45
Mitotic Spindle Checkpoint_2
9
Cell Cycle Checkpoints_3
28
Regulation of glycolysis by fructose 2,6-bisphospha...
5

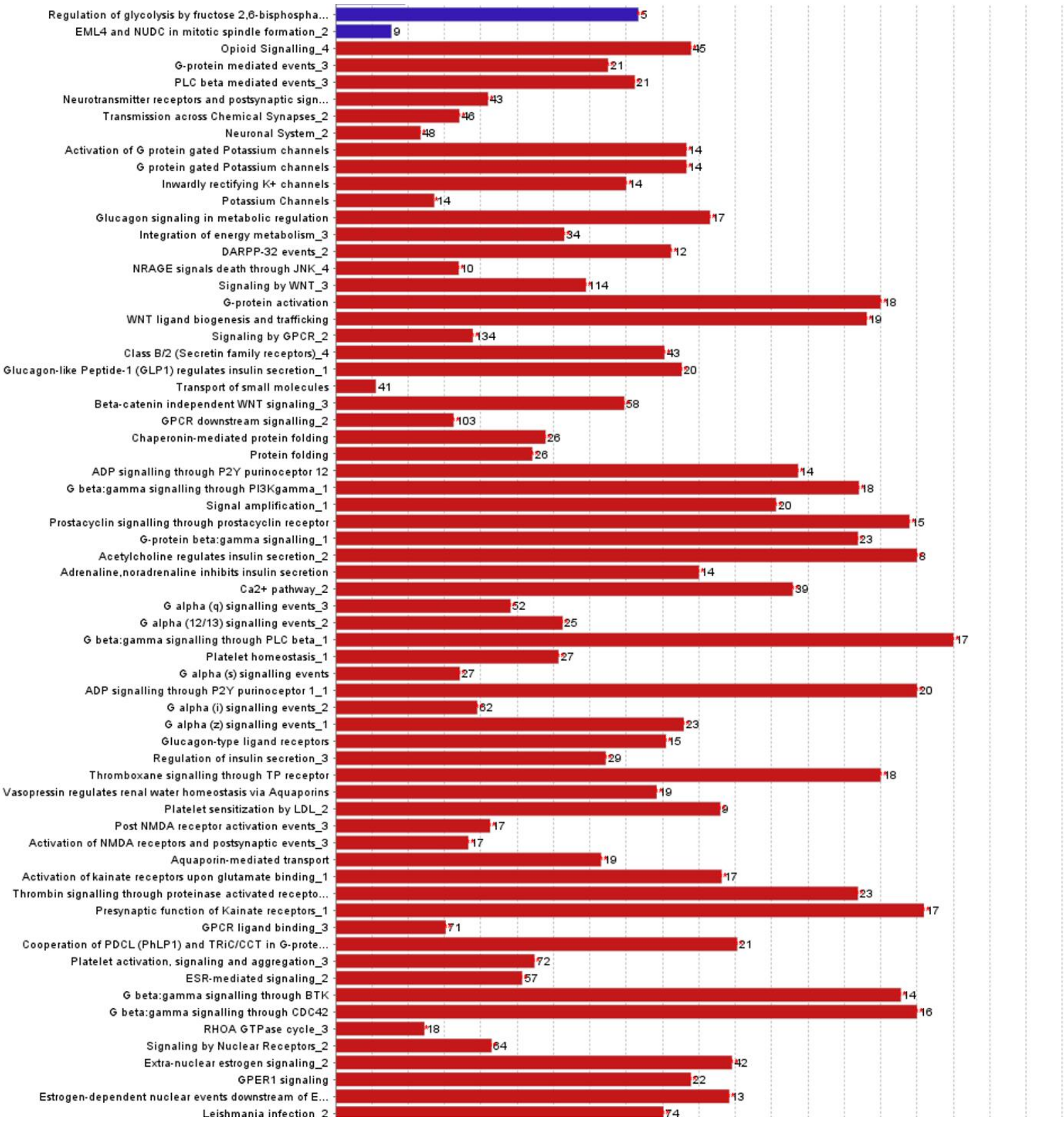
Regulation of glycolysis by fructose 2,6-bisphospha... 5
EML4 and NUDC in mitotic spindle formation_2 9
Opioid Signalling_4 45
G-protein mediated events_3 21
PLC beta mediated events_3 21
Neurotransmitter receptors and postsynaptic sign... 43
Transmission across Chemical Synapses_2 46
Neuronal System_2 48
Activation of G protein gated Potassium channels 14
G protein gated Potassium channels 14
Inwardly rectifying K+ channels 14
Potassium Channels 14
Glucagon signaling in metabolic regulation 17
Integration of energy metabolism_3 34
DARPP-32 events_2 12
NRAGE signals death through JNK_4 10
Signaling by WNT_3 114
G-protein activation 18
WNT ligand biogenesis and trafficking 19
Signaling by GPCR_2 134
Class B/2 (Secretin family receptors)_4 43
Glucagon-like Peptide-1 (GLP1) regulates insulin secretion_1 20
Transport of small molecules 41
Beta-catenin independent WNT signaling_3 58
GPCR downstream signalling_2 103
Chaperonin-mediated protein folding 26
Protein folding 26
ADP signalling through P2Y purinoceptor 12 14
G beta:gamma signalling through PI3Kgamma_1 18
Signal amplification_1 20
Prostacyclin signalling through prostacyclin receptor 15
G-protein beta:gamma signalling_1 23
Acetylcholine regulates insulin secretion_2 8
Adrenaline,noradrenaline inhibits insulin secretion 14
Ca2+ pathway_2 39
G alpha (q) signalling events_3 52
G alpha (12/13) signalling events_2 25
G beta:gamma signalling through PLC beta_1 17
Platelet homeostasis_1 27
G alpha (s) signalling events 27
ADP signalling through P2Y purinoceptor 1_1 20
G alpha (i) signalling events_2 62
G alpha (z) signalling events_1 23
Glucagon-type ligand receptors 15
Regulation of insulin secretion_3 29
Thromboxane signalling through TP receptor 18
Vasopressin regulates renal water homeostasis via Aquaporins 19
Platelet sensitization by LDL_2 9
Post NMDA receptor activation events_3 17
Activation of NMDA receptors and postsynaptic events_3 17
Aquaporin-mediated transport 19
Activation of kainate receptors upon glutamate binding_1 17
Thrombin signalling through proteinase activated recepto... 23
Presynaptic function of Kainate receptors_1 17
GPCR ligand binding_3 71
Cooperation of PDCL (PhLP1) and TRiC/CCT in G-prote... 21
Platelet activation, signaling and aggregation_3 72
ESR-mediated signaling_2 57
G beta:gamma signalling through BTK 14
G beta:gamma signalling through CDC42 16
RHOA GTPase cycle_3 18
Signaling by Nuclear Receptors_2 64
Extra-nuclear estrogen signaling_2 42
GPER1 signaling 22
Estrogen-dependent nuclear events downstream of E... 13
Leishmania infection_2 74

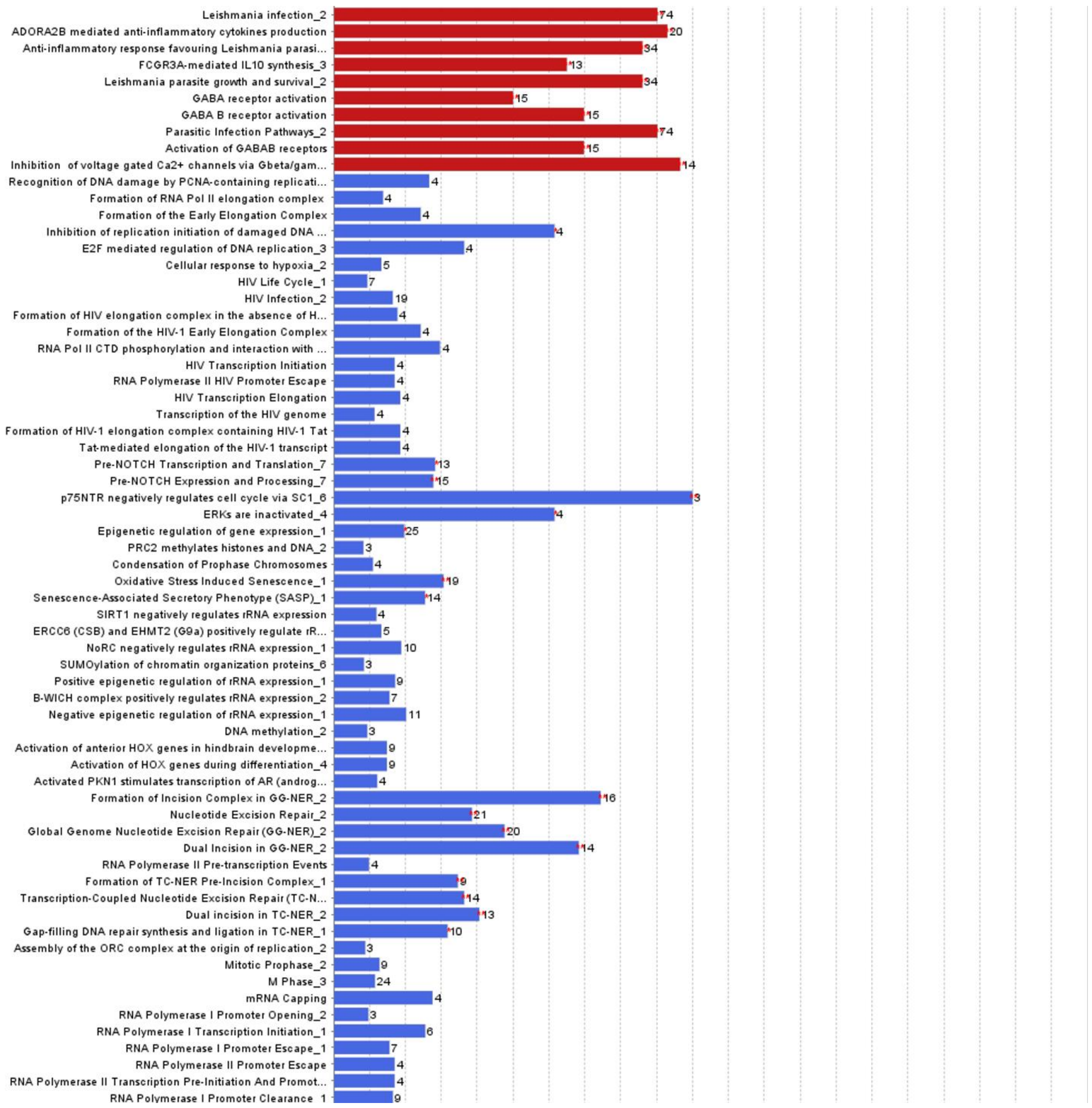

Leishmania infection_2 74
ADORA2B mediated anti-inflammatory cytokines production 20
Anti-inflammatory response favouring Leishmania parasi... 34
FCGR3A-mediated IL10 synthesis_3 13
Leishmania parasite growth and survival_2 34
GABA receptor activation 15
GABA B receptor activation 15
Parasitic Infection Pathways_2 74
Activation of GABAB receptors 15
Inhibition of voltage gated Ca2+ channels via Gbeta/gam... 14
Recognition of DNA damage by PCNA-containing replicati... 4
Formation of RNA Pol II elongation complex 4
Formation of the Early Elongation Complex 4
Inhibition of replication initiation of damaged DNA ... 4
E2F mediated regulation of DNA replication_3 4
Cellular response to hypoxia_2 5
HIV Life Cycle_1 7
HIV Infection_2 19
Formation of HIV elongation complex in the absence of H... 4
Formation of the HIV-1 Early Elongation Complex 4
RNA Pol II CTD phosphorylation and interaction with ... 4
HIV Transcription Initiation 4
RNA Polymerase II HIV Promoter Escape 4
HIV Transcription Elongation 4
Transcription of the HIV genome 4
Formation of HIV-1 elongation complex containing HIV-1 Tat 4
Tat-mediated elongation of the HIV-1 transcript 4
Pre-NOTCH Transcription and Translation_7 13
Pre-NOTCH Expression and Processing_7 15
p75NTR negatively regulates cell cycle via SC1_6 3
ERKs are inactivated_4 4
Epigenetic regulation of gene expression_1 25
PRC2 methylates histones and DNA_2 3
Condensation of Prophase Chromosomes 4
Oxidative Stress Induced Senescence_1 19
Senescence-Associated Secretory Phenotype (SASP)_1 14
SIRT1 negatively regulates rRNA expression 4
ERCC6 (CSB) and EHMT2 (G9a) positively regulate rR... 5
NoRC negatively regulates rRNA expression_1 10
SUMOylation of chromatin organization proteins_6 3
Positive epigenetic regulation of rRNA expression_1 9
B-WICH complex positively regulates rRNA expression_2 7
Negative epigenetic regulation of rRNA expression_1 11
DNA methylation_2 3
Activation of anterior HOX genes in hindbrain developme... 9
Activation of HOX genes during differentiation_4 9
Activated PKN1 stimulates transcription of AR (androg... 4
Formation of Incision Complex in GG-NER_2 16
Nucleotide Excision Repair_2 21
Global Genome Nucleotide Excision Repair (GG-NER)_2 20
Dual Incision in GG-NER_2 14
RNA Polymerase II Pre-transcription Events 4
Formation of TC-NER Pre-Incision Complex_1 9
Transcription-Coupled Nucleotide Excision Repair (TC-N... 14
Dual incision in TC-NER_2 13
Gap-filling DNA repair synthesis and ligation in TC-NER_1 10
Assembly of the ORC complex at the origin of replication_2 3
Mitotic Prophase_2 9
M Phase_3 24
mRNA Capping 4
RNA Polymerase I Promoter Opening_2 3
RNA Polymerase I Transcription Initiation_1 6
RNA Polymerase I Promoter Escape_1 7
RNA Polymerase II Promoter Escape 4
RNA Polymerase II Transcription Pre-Initiation And Promot... 4
RNA Polymerase I Promoter Clearance_1 9

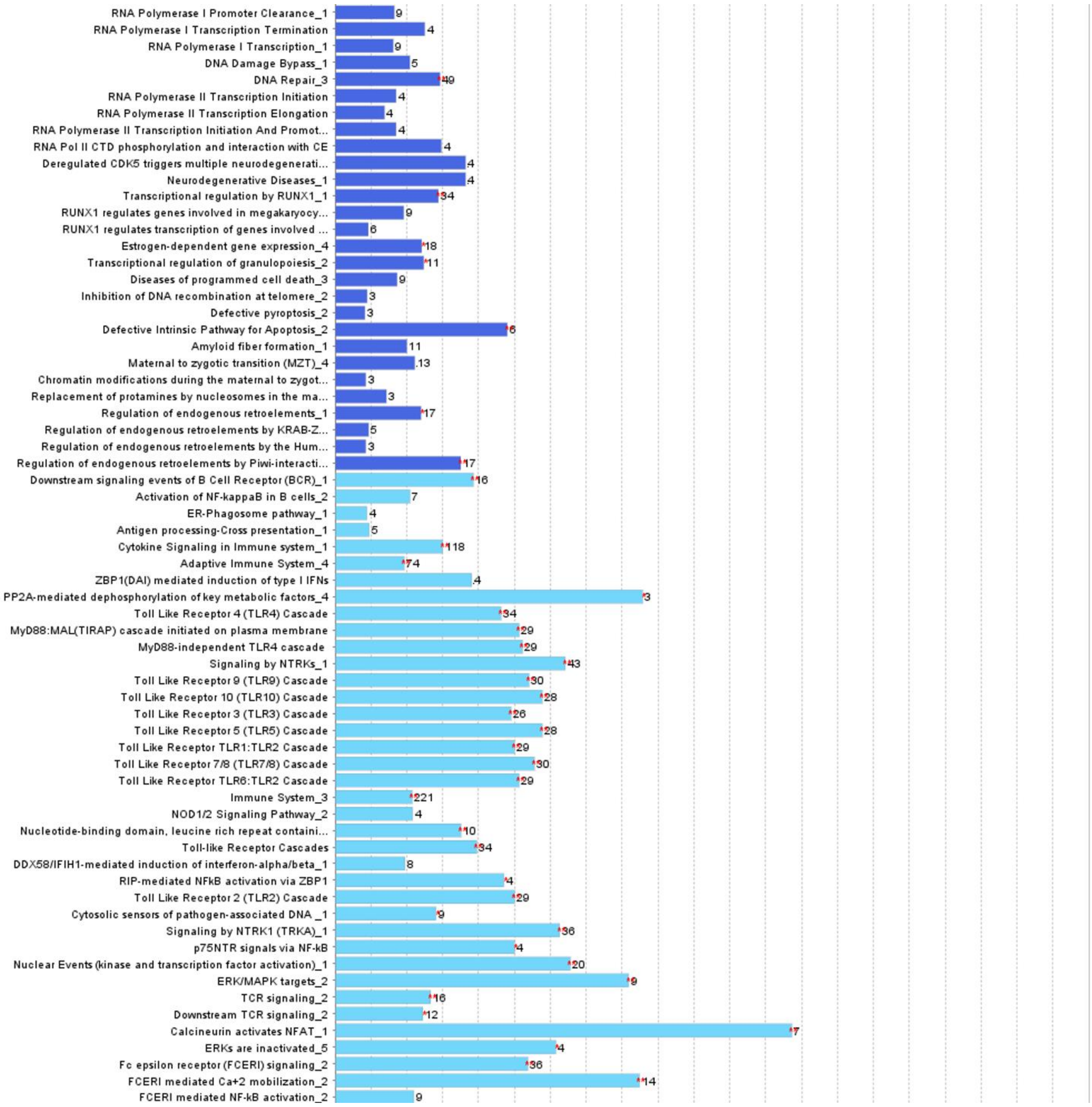
RNA Polymerase I Promoter Clearance_1 9
RNA Polymerase I Transcription Termination 4
RNA Polymerase I Transcription_1 9
DNA Damage Bypass_1 5
DNA Repair_3 49
RNA Polymerase II Transcription Initiation 4
RNA Polymerase II Transcription Elongation 4
RNA Polymerase II Transcription Initiation And Promot... 4
RNA Pol II CTD phosphorylation and interaction with CE 4
Deregulated CDK5 triggers multiple neurodegenerati... 4
Neurodegenerative Diseases_1 4
Transcriptional regulation by RUNX1_1 34
RUNX1 regulates genes involved in megakaryocy... 9
RUNX1 regulates transcription of genes involved ... 6
Estrogen-dependent gene expression_4 18
Transcriptional regulation of granulopoiesis_2 11
Diseases of programmed cell death_3 9
Inhibition of DNA recombination at telomere_2 3
Defective pyroptosis_2 3
Defective Intrinsic Pathway for Apoptosis_2 6
Amyloid fiber formation_1 11
Maternal to zygotic transition (MZT)_4 13
Chromatin modifications during the maternal to zygot... 3
Replacement of protamines by nucleosomes in the ma... 3
Regulation of endogenous retroelements_1 17
Regulation of endogenous retroelements by KRAB-Z... 5
Regulation of endogenous retroelements by the Hum... 3
Regulation of endogenous retroelements by Piwi-interacti... 17
Downstream signaling events of B Cell Receptor (BCR)_1 16
Activation of NF-kappaB in B cells_2 7
ER-Phagosome pathway_1 4
Antigen processing-Cross presentation_1 5
Cytokine Signaling in Immune system_1 118
Adaptive Immune System_4 74
ZBP1(DAI) mediated induction of type I IFNs 4
PP2A-mediated dephosphorylation of key metabolic factors_4 3
Toll Like Receptor 4 (TLR4) Cascade 34
MyD88:MAL(TIRAP) cascade initiated on plasma membrane 29
MyD88-independent TLR4 cascade 29
Signaling by NTRKs_1 43
Toll Like Receptor 9 (TLR9) Cascade 30
Toll Like Receptor 10 (TLR10) Cascade 28
Toll Like Receptor 3 (TLR3) Cascade 26
Toll Like Receptor 5 (TLR5) Cascade 28
Toll Like Receptor TLR1:TLR2 Cascade 29
Toll Like Receptor 7/8 (TLR7/8) Cascade 30
Toll Like Receptor TLR6:TLR2 Cascade 29
Immune System_3 221
NOD1/2 Signaling Pathway_2 4
Nucleotide-binding domain, leucine rich repeat containi... 10
Toll-like Receptor Cascades 34
DDX58/IFIH1-mediated induction of interferon-alpha/beta_1 8
RIP-mediated NFkB activation via ZBP1 4
Toll Like Receptor 2 (TLR2) Cascade 29
Cytosolic sensors of pathogen-associated DNA _1 9
Signaling by NTRK1 (TRKA)_1 36
p75NTR signals via NF-kB 4
Nuclear Events (kinase and transcription factor activation)_1 20
ERK/MAPK targets_2 9
TCR signaling_2 16
Downstream TCR signaling_2 12
Calcineurin activates NFAT_1 7
ERKs are inactivated_5 4
Fc epsilon receptor (FCERI) signaling_2 36
FCERI mediated Ca+2 mobilization_2 14
FCERI mediated NF-kB activation_2 9

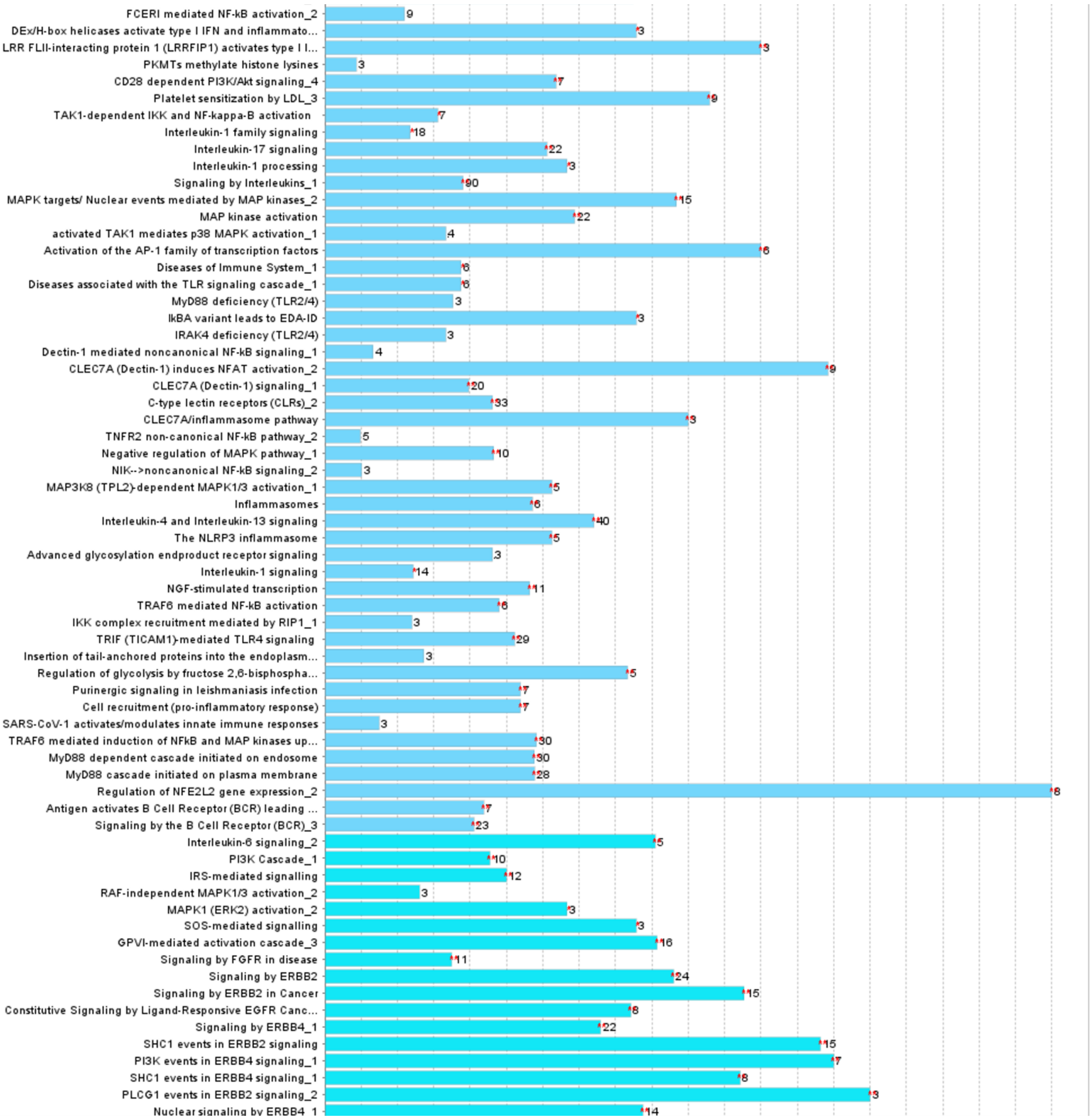
FCERI mediated NF-kB activation_2
DEx/H-box helicases activate type I IFN and inflammato...
LRR FLII-interacting protein 1 (LRRFIP1) activates type I I...
PKMTs methylate histone lysines
CD28 dependent PI3K/Akt signaling_4
Platelet sensitization by LDL_3
TAK1-dependent IKK and NF-kappa-B activation
Interleukin-1 family signaling
Interleukin-17 signaling
Interleukin-1 processing
Signaling by Interleukins_1
MAPK targets/ Nuclear events mediated by MAP kinases_2
MAP kinase activation
activated TAK1 mediates p38 MAPK activation_1
Activation of the AP-1 family of transcription factors
Diseases of Immune System_1
Diseases associated with the TLR signaling cascade_1
MyD88 deficiency (TLR2/4)
IkBA variant leads to EDA-ID
IRAK4 deficiency (TLR2/4)
Dectin-1 mediated noncanonical NF-kB signaling_1
CLEC7A (Dectin-1) induces NFAT activation_2
CLEC7A (Dectin-1) signaling_1
C-type lectin receptors (CLRs)_2
CLEC7A/inflammasome pathway
TNFR2 non-canonical NF-kB pathway_2
Negative regulation of MAPK pathway_1
NIK-->noncanonical NF-kB signaling_2
MAP3K8 (TPL2)-dependent MAPK1/3 activation_1
Inflammasomes
Interleukin-4 and Interleukin-13 signaling
The NLRP3 inflammasome
Advanced glycosylation endproduct receptor signaling
Interleukin-1 signaling
NGF-stimulated transcription
TRAF6 mediated NF-kB activation
IKK complex recruitment mediated by RIP1_1
TRIF (TICAM1)-mediated TLR4 signaling
Insertion of tail-anchored proteins into the endoplasm...
Regulation of glycolysis by fructose 2,6-bisphospha...
Purinergic signaling in leishmaniasis infection
Cell recruitment (pro-inflammatory response)
SARS-CoV-1 activates/modulates innate immune responses
TRAF6 mediated induction of NFkB and MAP kinases up...
MyD88 dependent cascade initiated on endosome
MyD88 cascade initiated on plasma membrane
Regulation of NFE2L2 gene expression_2
Antigen activates B Cell Receptor (BCR) leading ...
Signaling by the B Cell Receptor (BCR)_3
Interleukin-6 signaling_2
PI3K Cascade_1
IRS-mediated signalling
RAF-independent MAPK1/3 activation_2
MAPK1 (ERK2) activation_2
SOS-mediated signalling
GPVI-mediated activation cascade_3
Signaling by FGFR in disease
Signaling by ERBB2
Signaling by ERBB2 in Cancer
Constitutive Signaling by Ligand-Responsive EGFR Canc...
Signaling by ERBB4_1
SHC1 events in ERBB2 signaling
PI3K events in ERBB4 signaling_1
SHC1 events in ERBB4 signaling_1
PLCG1 events in ERBB2 signaling_2
Nuclear signaling by ERBB4_1
9
3
3
3
7
9
7
18
22
3
90
15
22
4
6
6
6
3
3
3
4
9
20
33
3
5
10
3
5
6
40
5
3
14
11
6
3
29
3
5
7
7
3
30
30
28
8
7
23
5
10
12
3
3
3
16
11
24
15
8
22
15
7
8
3
14

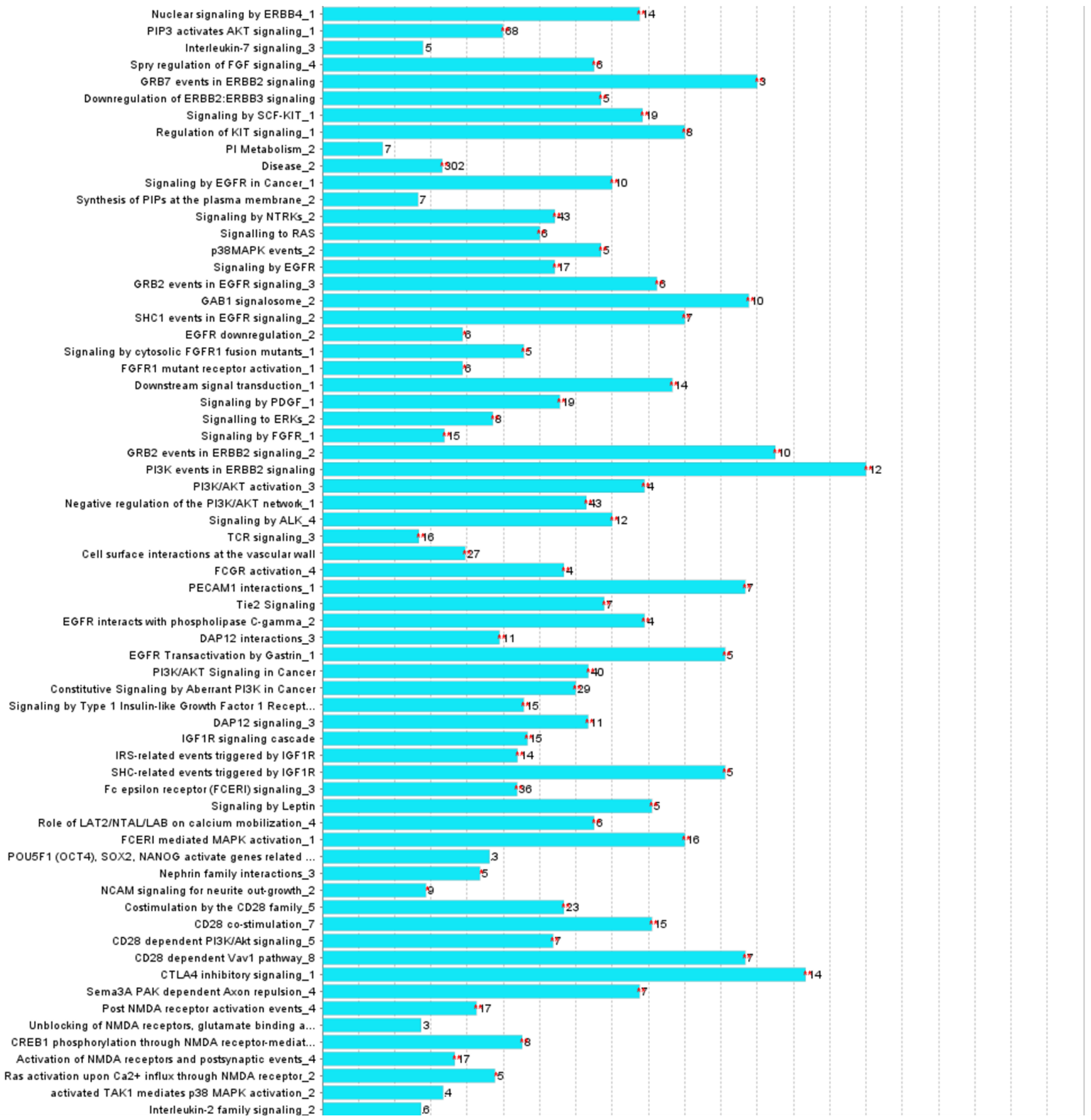

Nuclear signaling by ERBB4_1
14
PIP3 activates AKT signaling_1
68
Interleukin-7 signaling_3
5
Spry regulation of FGF signaling_4
6
GRB7 events in ERBB2 signaling
3
Downregulation of ERBB2:ERBB3 signaling
5
Signaling by SCF-KIT_1
19
Regulation of KIT signaling_1
8
PI Metabolism_2
7
Disease_2
302
Signaling by EGFR in Cancer_1
10
Synthesis of PIPs at the plasma membrane_2
7
Signaling by NTRKs_2
43
Signalling to RAS
6
p38MAPK events_2
5
Signaling by EGFR
17
GRB2 events in EGFR signaling_3
6
GAB1 signalosome_2
10
SHC1 events in EGFR signaling_2
7
EGFR downregulation_2
6
Signaling by cytosolic FGFR1 fusion mutants_1
5
FGFR1 mutant receptor activation_1
6
Downstream signal transduction_1
14
Signaling by PDGF_1
19
Signalling to ERKs_2
8
Signaling by FGFR_1
15
GRB2 events in ERBB2 signaling_2
10
PI3K events in ERBB2 signaling
12
PI3K/AKT activation_3
4
Negative regulation of the PI3K/AKT network_1
43
Signaling by ALK_4
12
TCR signaling_3
16
Cell surface interactions at the vascular wall
27
FCGR activation_4
4
PECAM1 interactions_1
7
Tie2 Signaling
7
EGFR interacts with phospholipase C-gamma_2
4
DAP12 interactions_3
11
EGFR Transactivation by Gastrin_1
5
PI3K/AKT Signaling in Cancer
40
Constitutive Signaling by Aberrant PI3K in Cancer
29
Signaling by Type 1 Insulin-like Growth Factor 1 Recept...
15
DAP12 signaling_3
11
IGF1R signaling cascade
15
IRS-related events triggered by IGF1R
14
SHC-related events triggered by IGF1R
5
Fc epsilon receptor (FCERI) signaling_3
36
Signaling by Leptin
5
Role of LAT2/NTAL/LAB on calcium mobilization_4
6
FCERI mediated MAPK activation_1
16
POU5F1 (OCT4), SOX2, NANOG activate genes related ...
3
Nephrin family interactions_3
5
NCAM signaling for neurite out-growth_2
9
Costimulation by the CD28 family_5
23
CD28 co-stimulation_7
15
CD28 dependent PI3K/Akt signaling_5
7
CD28 dependent Vav1 pathway_8
7
CTLA4 inhibitory signaling_1
14
Sema3A PAK dependent Axon repulsion_4
7
Post NMDA receptor activation events_4
17
Unblocking of NMDA receptors, glutamate binding a...
3
CREB1 phosphorylation through NMDA receptor-mediat...
8
Activation of NMDA receptors and postsynaptic events_4
17
Ras activation upon Ca2+ influx through NMDA receptor_2
5
activated TAK1 mediates p38 MAPK activation_2
4
Interleukin-2 family signaling_2
6

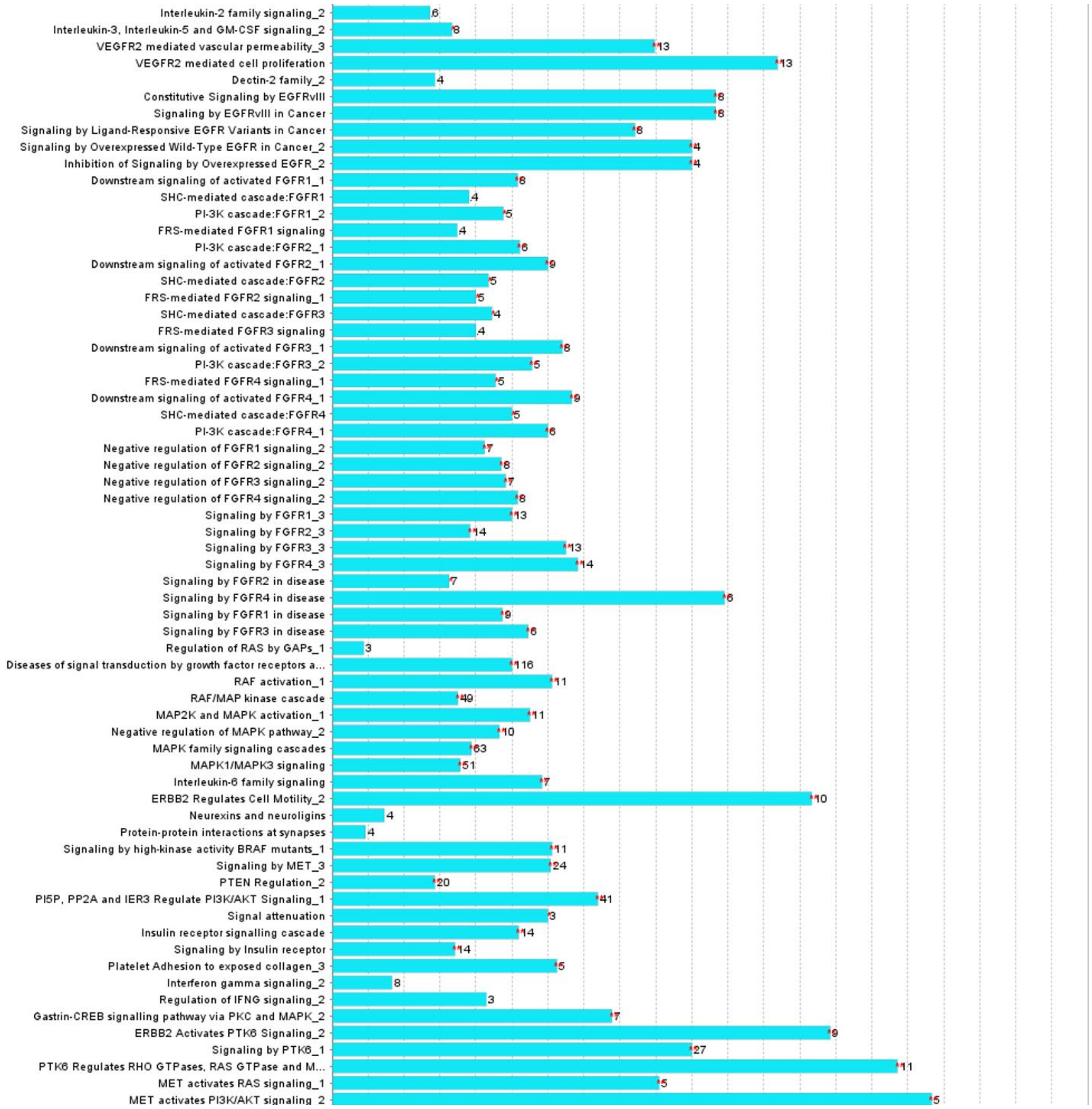

Interleukin-2 family signaling_2 6
Interleukin-3, Interleukin-5 and GM-CSF signaling_2 8
VEGFR2 mediated vascular permeability_3 13
VEGFR2 mediated cell proliferation 13
Dectin-2 family_2 4
Constitutive Signaling by EGFRvIII 8
Signaling by EGFRvIII in Cancer 8
Signaling by Ligand-Responsive EGFR Variants in Cancer 8
Signaling by Overexpressed Wild-Type EGFR in Cancer_2 4
Inhibition of Signaling by Overexpressed EGFR_2 4
Downstream signaling of activated FGFR1_1 8
SHC-mediated cascade:FGFR1 4
PI-3K cascade:FGFR1_2 5
FRS-mediated FGFR1 signaling 4
PI-3K cascade:FGFR2_1 6
Downstream signaling of activated FGFR2_1 9
SHC-mediated cascade:FGFR2 5
FRS-mediated FGFR2 signaling_1 5
SHC-mediated cascade:FGFR3 4
FRS-mediated FGFR3 signaling 4
Downstream signaling of activated FGFR3_1 8
PI-3K cascade:FGFR3_2 5
FRS-mediated FGFR4 signaling_1 5
Downstream signaling of activated FGFR4_1 9
SHC-mediated cascade:FGFR4 5
PI-3K cascade:FGFR4_1 6
Negative regulation of FGFR1 signaling_2 7
Negative regulation of FGFR2 signaling_2 8
Negative regulation of FGFR3 signaling_2 7
Negative regulation of FGFR4 signaling_2 8
Signaling by FGFR1_3 13
Signaling by FGFR2_3 14
Signaling by FGFR3_3 13
Signaling by FGFR4_3 14
Signaling by FGFR2 in disease 7
Signaling by FGFR4 in disease 6
Signaling by FGFR1 in disease 9
Signaling by FGFR3 in disease 6
Regulation of RAS by GAPs_1 3
Diseases of signal transduction by growth factor receptors a... 116
RAF activation_1 11
RAF/MAP kinase cascade 49
MAP2K and MAPK activation_1 11
Negative regulation of MAPK pathway_2 10
MAPK family signaling cascades 63
MAPK1/MAPK3 signaling 51
Interleukin-6 family signaling 7
ERBB2 Regulates Cell Motility_2 10
Neurexins and neuroligins 4
Protein-protein interactions at synapses 4
Signaling by high-kinase activity BRAF mutants_1 11
Signaling by MET_3 24
PTEN Regulation_2 20
PI5P, PP2A and IER3 Regulate PI3K/AKT Signaling_1 41
Signal attenuation 3
Insulin receptor signalling cascade 14
Signaling by Insulin receptor 14
Platelet Adhesion to exposed collagen_3 5
Interferon gamma signaling_2 8
Regulation of IFNG signaling_2 3
Gastrin-CREB signalling pathway via PKC and MAPK_2 7
ERBB2 Activates PTK6 Signaling_2 9
Signaling by PTK6_1 27
PTK6 Regulates RHO GTPases, RAS GTPase and M... 11
MET activates RAS signaling_1 5
MET activates PI3K/AKT signaling_2 5

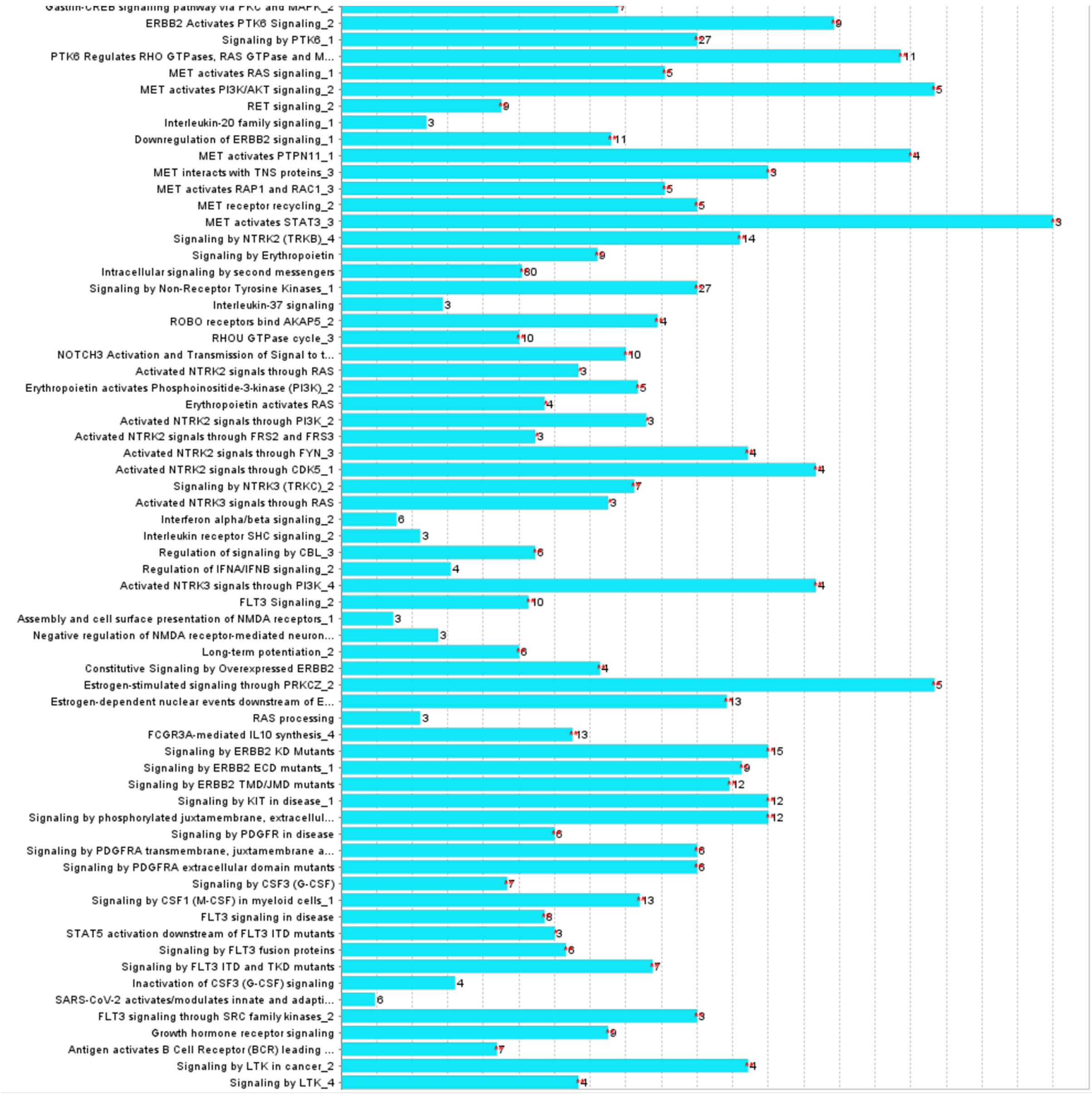
ERBB2 Activates PTK6 Signaling_2
9
Signaling by PTK6_1
27
PTK6 Regulates RHO GTPases, RAS GTPase and M...
11
MET activates RAS signaling_1
5
MET activates PI3K/AKT signaling_2
5
RET signaling_2
9
Interleukin-20 family signaling_1
3
Downregulation of ERBB2 signaling_1
11
MET activates PTPN11_1
4
MET interacts with TNS proteins_3
3
MET activates RAP1 and RAC1_3
5
MET receptor recycling_2
5
MET activates STAT3_3
3
Signaling by NTRK2 (TRKB)_4
14
Signaling by Erythropoietin
9
Intracellular signaling by second messengers
80
Signaling by Non-Receptor Tyrosine Kinases_1
27
Interleukin-37 signaling
3
ROBO receptors bind AKAP5_2
4
RHOU GTPase cycle_3
10
NOTCH3 Activation and Transmission of Signal to t...
10
Activated NTRK2 signals through RAS
3
Erythropoietin activates Phosphoinositide-3-kinase (PI3K)_2
5
Erythropoietin activates RAS
4
Activated NTRK2 signals through PI3K_2
3
Activated NTRK2 signals through FRS2 and FRS3
3
Activated NTRK2 signals through FYN_3
4
Activated NTRK2 signals through CDK5_1
4
Signaling by NTRK3 (TRKC)_2
7
Activated NTRK3 signals through RAS
3
Interferon alpha/beta signaling_2
6
Interleukin receptor SHC signaling_2
3
Regulation of signaling by CBL_3
6
Regulation of IFNA/IFNB signaling_2
4
Activated NTRK3 signals through PI3K_4
4
FLT3 Signaling_2
10
Assembly and cell surface presentation of NMDA receptors_1
3
Negative regulation of NMDA receptor-mediated neuron...
3
Long-term potentiation_2
6
Constitutive Signaling by Overexpressed ERBB2
4
Estrogen-stimulated signaling through PRKCZ_2
5
Estrogen-dependent nuclear events downstream of E...
13
RAS processing
3
FCGR3A-mediated IL10 synthesis_4
13
Signaling by ERBB2 KD Mutants
15
Signaling by ERBB2 ECD mutants_1
9
Signaling by ERBB2 TMD/JMD mutants
12
Signaling by KIT in disease_1
12
Signaling by phosphorylated juxtamembrane, extracellul...
12
Signaling by PDGFR in disease
6
Signaling by PDGFRA transmembrane, juxtamembrane a...
6
Signaling by PDGFRA extracellular domain mutants
6
Signaling by CSF3 (G-CSF)
7
Signaling by CSF1 (M-CSF) in myeloid cells_1
13
FLT3 signaling in disease
8
STAT5 activation downstream of FLT3 ITD mutants
3
Signaling by FLT3 fusion proteins
6
Signaling by FLT3 ITD and TKD mutants
7
Inactivation of CSF3 (G-CSF) signaling
4
SARS-CoV-2 activates/modulates innate and adapti...
6
FLT3 signaling through SRC family kinases_2
3
Growth hormone receptor signaling
9
Antigen activates B Cell Receptor (BCR) leading ...
7
Signaling by LTK in cancer_2
4
Signaling by LTK_4
4

Table 4S: Terms/signaling pathways in GA-related GIM (sorted by topological coefficient with p values corrected corrected with Benjamini-Hochberg

Term	Topological Coefficient	P value	UNIQUE_ID
**Cytochrome c-mediated apoptotic response**	1.0	0.03077941836978011	R-HSA:111461
**COPI-mediated anterograde transport**	1.0	0.032303979478631784	R-HSA:6807878
**Membrane Trafficking**	0.95	0.017147580936152578	R-HSA:199991
**Vesicle-mediated transport**	0.95	0.00996277877449441	R-HSA:5653656
**Laminin interactions**	0.9285714285714286	0.029830001939537696	R-HSA:3000157
**Apoptosis**	0.9166666666666666	0.032285379855300216	R-HSA:109581
**Programmed Cell Death**	0.9166666666666666	0.04872571128296693	R-HSA:5357801
**Loss of function of MECP2 in Rett syndrome**	0.8666666666666667	0.033001603260751265	R-HSA:9005891
**Pervasive developmental disorders**	0.8666666666666667	0.033001603260751265	R-HSA:9005895
**Loss of MECP2 binding ability to the NCoR/SMRT complex**	0.8666666666666667	0.032293221903259124	R-HSA:9022537
**Regulation of MECP2 expression and activity**	0.8666666666666667	0.018089301915738484	R-HSA:9022692
**Disorders of Nervous System Development**	0.8666666666666667	0.033001603260751265	R-HSA:9697154
**ER to Golgi Anterograde Transport**	0.84	0.017041728977493436	R-HSA:199977
**Asparagine N-linked glycosylation**	0.84	0.02818743056405486	R-HSA:446203
**Transport to the Golgi and subsequent modification**	0.84	0.03545172222555464	R-HSA:948021
**Platelet degranulation**	0.83	0.017316021878206047	R-HSA:114608
**Response to elevated platelet cytosolic Ca2+**	0.83	0.01790211946767316	R-HSA:76005
**Regulation of TLR by endogenous ligand**	0.83	0.03194047855949558	R-HSA:5686938

**Signaling by high-kinase activity BRAF mutants**	0.83	0.046466189073031405	R-HSA:6802948
**Signaling by BRAF and RAF1 fusions**	0.83	0.03641437116125538	R-HSA:6802952
**Intrinsic Pathway for Apoptosis**	0.75	0.03640015876644282	R-HSA:109606
**Apoptotic factor-mediated response**	0.75	0.02817738778075856	R-HSA:111471
**Cellular responses to stress**	0.75	0.005276693628432567	R-HSA:2262752
**Infectious disease**	0.75	0.03246340881112912	R-HSA:5663205
**Cellular responses to stimuli**	0.75	0.006796688699894478	R-HSA:8953897
**Viral Infection Pathways**	0.75	0.02974680918396593	R-HSA:9824446
**Extracellular matrix organization**	0.73	0.0322963648151965	R-HSA:1474244
**Integrin signaling**	0.70	0.02408799556758114	R-HSA:354192
**Signaling by PDGF**	0.70	0.04416119758411886	R-HSA:186797
**Cytoprotection by HMOX1**	0.70	0.04816227552410113	R-HSA:9707564
**MyD88:MAL(TIRAP) cascade initiated on plasma membrane**	0.69	0.04947705503115662	R-HSA:166058
**Toll Like Receptor TLR6:TLR2 Cascade**	0.69	0.04947705503115662	R-HSA:168188
**Mitotic Metaphase and Anaphase**	0.68	0.021731129328632234	R-HSA:2555396
**Mitotic Anaphase**	0.68	0.021905342301370867	R-HSA:68882
**rRNA modification in the nucleus and cytosol**	0.66	0.01716009127428512	R-HSA:6790901
**rRNA processing in the nucleus and cytosol**	0.66	0.010071008918313544	R-HSA:8868773
**Assembly of collagen fibrils and other multimeric structures**	0.60	0.03110394719900843	R-HSA:2022090
**ECM proteoglycans**	0.60	0.03283706291377266	R-HSA:3000178
**Collagen chain trimerization**	0.60	0.036675012554702416	R-HSA:8948216
**Cell Cycle**	0.59	0.018467376538773828	R-HSA:1640170
**Cell Cycle, Mitotic**	0.59	0.02168314406519144	R-HSA:69278
**DARPP-32 events**	0.58	0.02205908844509303	R-HSA:180024

**Platelet sensitization by LDL**	0.58	0.006716243284585191	R-HSA:432142
**NCAM signaling for neurite out-growth**	0.58	0.033035631927149575	R-HSA:375165
**mRNA 3'-end processing**	0.58	0.047197336404730485	R-HSA:72187
**Transport of Mature Transcript to Cytoplasm**	0.58	0.01733189514184999	R-HSA:72202
**Disorders of Developmental Biology**	0.58	0.033001603260751265	R-HSA:9675151
**mRNA Splicing - Major Pathway**	0.55	3.1770569039773987E-7	R-HSA:72163
**mRNA Splicing**	0.55	6.383198641071852E-7	R-HSA:72172
**GRB2:SOS provides linkage to MAPK signaling for Integrins**	0.55	0.03646864389235669	R-HSA:354194
**p130Cas linkage to MAPK signaling for integrins**	0.55	0.03646864389235669	R-HSA:372708
**Mitotic G2-G2/M phases**	0.53	0.04904359332420217	R-HSA:453274
**G2/M Transition**	0.53	0.04842934856210643	R-HSA:69275
**CTLA4 inhibitory signaling**	0.52	0.03043086994374417	R-HSA:389513
**Nuclear Envelope (NE) Reassembly**	0.52	0.018627279867871778	R-HSA:2995410
**Signaling by moderate kinase activity BRAF mutants**	0.51	0.045986472185865504	R-HSA:6802946
**Signaling by RAS mutants**	0.51	0.045986472185865504	R-HSA:6802949
**Paradoxical activation of RAF signaling by kinase inactive BRAF**	0.51	0.045986472185865504	R-HSA:6802955
**Signaling downstream of RAS mutants**	0.51	0.045986472185865504	R-HSA:9649948
**Signaling by RAF1 mutants**	0.51	0.03510154802392978	R-HSA:9656223
**M Phase**	0.51	0.021703782367382473	R-HSA:68886
**Aerobic respiration and respiratory electron transport**	0.50	0.03502850716909681	R-HSA:1428517
**snRNP Assembly**	0.50	0.03254398990402931	R-HSA:191859

**MASTL Facilitates Mitotic Progression**	0.50	0.017717009334540117	R-HSA:2465910
**Metabolism of proteins**	0.50	0.009207695633988296	R-HSA:392499
**Axon guidance**	0.50	0.016785594278522928	R-HSA:422475
**mRNA decay by 3' to 5' exoribonuclease**	0.50	0.04195902417660218	R-HSA:429958
**MAPK targets/ Nuclear events mediated by MAP kinases**	0.50	0.03284753842766893	R-HSA:450282
**TP53 Regulates Metabolic Genes**	0.50	0.04723898496996881	R-HSA:5628897
**Metabolism of amino acids and derivatives**	0.50	0.006803327675130781	R-HSA:71291
**Translation**	0.50	0.036856802790047424	R-HSA:72766
**Glycogen metabolism**	0.50	0.049312230020330465	R-HSA:8982491
**ERK/MAPK targets**	0.45	0.03194047855949558	R-HSA:198753
**Cellular response to chemical stress**	0.45	0.0236541804627481	R-HSA:9711123
**Cyclin A/B1/B2 associated events during G2/M transition**	0.44	0.041876234163061854	R-HSA:69273
**SRP-dependent cotranslational protein targeting to membrane**	0.42	0.0326069815374156	R-HSA:1799339
**Selenoamino acid metabolism**	0.42	0.025768346695481757	R-HSA:2408522
**RAF activation**	0.42	0.046466189073031405	R-HSA:5673000
**Transport of Mature mRNA derived from an Intron-Containing Transcript**	0.42	0.018627279867871778	R-HSA:159236
**Major pathway of rRNA processing in the nucleolus and cytosol**	0.40	0.025460446702795705	R-HSA:6791226
**rRNA processing**	0.40	0.017418965543868823	R-HSA:72312
**Platelet activation, signaling and aggregation**	0.38	0.036958991533182606	R-HSA:76002
**Integrin cell surface interactions**	0.38	0.04840319342321396	R-HSA:216083
**Processing of Capped Intron-**	0.38	1.2863864573279597E-7	R-HSA:72203

**Containing Pre-mRNA**			
**PP2A-mediated dephosphorylation of key metabolic factors**	0.38	0.032293221903259124	R-HSA:163767
**Regulation of glycolysis by fructose 2,6-bisphosphate metabolism**	0.38	0.026713025026585815	R-HSA:9634600
**Metabolism of non-coding RNA**	0.375	0.03254398990402931	R-HSA:194441
**Mitotic Prophase**	0.35	0.032184383060804116	R-HSA:68875
**ERKs are inactivated**	0.34	0.03077941836978011	R-HSA:202670
**Metabolism of carbohydrates**	0.33	0.04825103104814824	R-HSA:71387
**Disease**	0.32	0.04175717465660903	R-HSA:1643685
**Glycolysis**	0.31	0.030479583649003734	R-HSA:70171
**Glucose metabolism**	0.31	0.04723898496996881	R-HSA:70326
**Metabolism**	0.25	2.783842857413548E-4	R-HSA:1430728
**Deadenylation-dependent mRNA decay**	0.25	1.8880161142073435E-4	R-HSA:429914
**Metabolism of RNA**	0.24	1.653600516017647E-12	R-HSA:8953854
**Glutathione conjugation**	0.0	0.046466189073031405	R-HSA:156590
**Metabolism of nucleotides**	0.0	0.04890606133035483	R-HSA:15869
**HIV Life Cycle**	0.0	0.04157605624452897	R-HSA:162587
**Late Phase of HIV Life Cycle**	0.0	0.030698987338338448	R-HSA:162599
**Innate Immune System**	0.0	0.047184009545942	R-HSA:168249
**Initiation of Nuclear Envelope (NE) Reformation**	0.0	0.024990348646255302	R-HSA:2995383
**Scavenging by Class A Receptors**	0.0	0.024990348646255302	R-HSA:3000480
**Glycogen synthesis**	0.0	0.033001603260751265	R-HSA:3322077
**Transcriptional Regulation by TP53**	0.0	0.03194405399919193	R-HSA:3700989
**mRNA decay by 5' to 3' exoribonuclease**	0.0	0.017586923244728072	R-HSA:430039
**Smooth Muscle Contraction**	0.0	0.03943813508117212	R-HSA:445355

**Synthesis of GDP-mannose**	0.0	0.026335567668901057	R-HSA:446205
**Butyrate Response Factor 1 (BRF1) binds and destabilizes mRNA**	0.0	0.04730442120502561	R-HSA:450385
**Fructose metabolism**	0.0	0.032293221903259124	R-HSA:5652084
**Diseases of signal transduction by growth factor receptors and second messengers**	0.0	0.03543770700709452	R-HSA:5663202
**Respiratory electron transport**	0.0	0.017519376237775018	R-HSA:611105
**Signaling by PTK6**	0.0	0.020093446265703075	R-HSA:8848021
**Signaling by Non-Receptor Tyrosine Kinases**	0.0	0.020093446265703075	R-HSA:9006927
**Nervous system development**	0.0	0.017784086016500188	R-HSA:9675108
**M-decay: degradation of maternal mRNAs by maternally stored factors**	0.0	0.03675061929732784	R-HSA:9820841
**Respiratory syncytial virus (RSV) genome replication, transcription and translation**	0.0	0.04195902417660218	R-HSA:9820965
**Maturation of hRSV A proteins**	0.0	0.03077941836978011	R-HSA:9828806
**Mitochondrial protein degradation**	0.0	0.04848782603948083	R-HSA:9837999

Table S5: 330 out of 865 hub proteins with PPI >3 in GA-related GIM

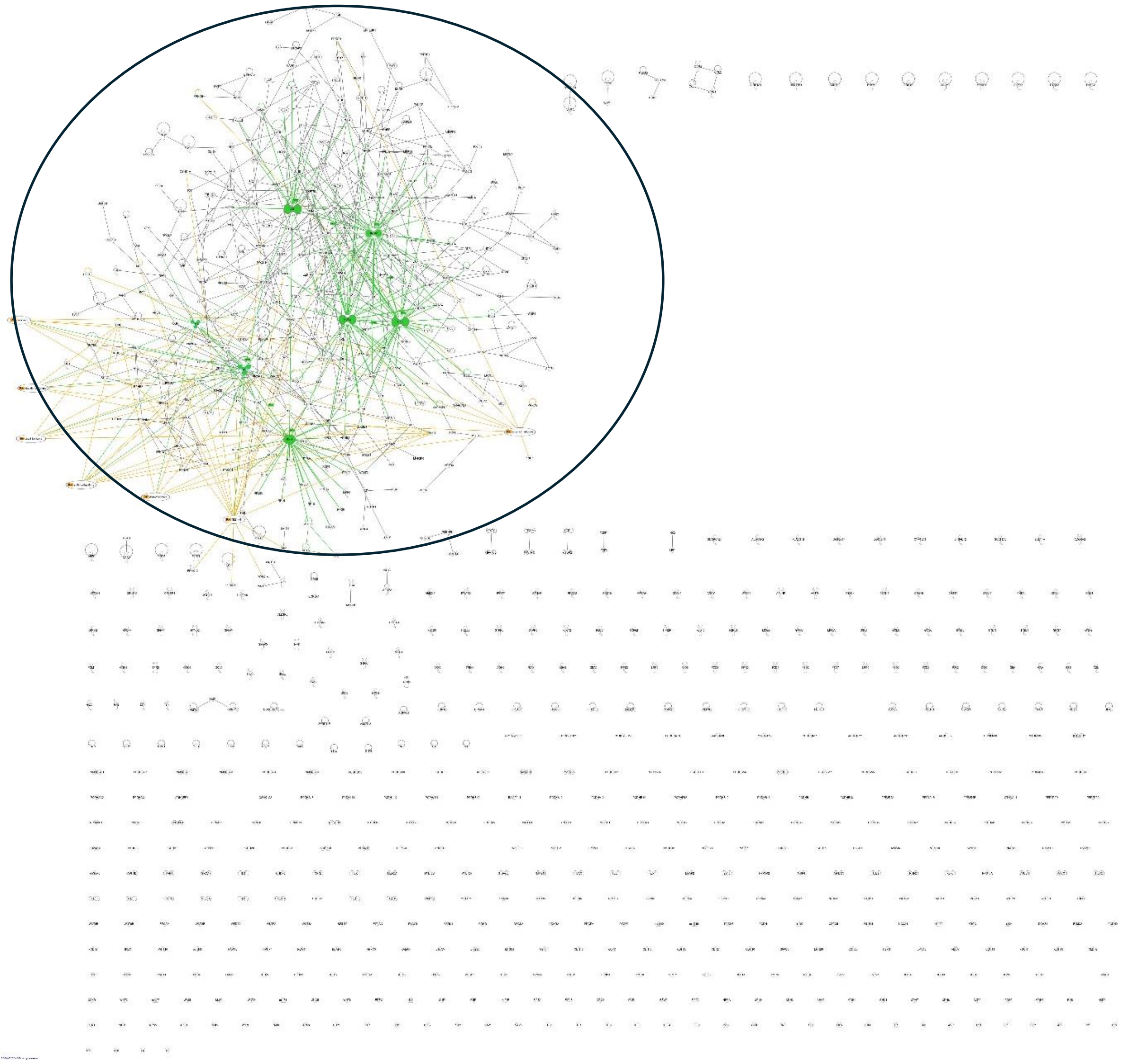

ABI1
ABL1
ACTB
ACTG1
ACTR2
ACTR3
AIRE
AKT1
AP2A2
AP2B1
AP2M1
AP2S1
APC

APP
AR
AREG
ARHGEF7
ARPC2
ARPC5
ARR3
ARRB1
ARRB2
ATF2
ATF4
ATM
ATR
AXIN1
AXL
BCAR1
BCL2
BCR
BMP7
BMPR2
BMPR1B
BRCA1
BRCA2
BTRC
CASP3
CCN2
CCND1
CCNH
CD44
CD40LG
CDC42
CDH1
CDH5
CDKN1A
CDKN1B
CDKN2A
CFL1
CHEK1
CHEK2
CHUK
COL1A1
CREB1
CREBBP
CRK
CSF1
CSNK1A1
CSNK1D
CSNK1G3

CSNK2A1
CSNK2A2
CSNK2B
CTBP1
CTNNA1
CTNNB1
CTNND1
CTTN
CUL4A
CUL4B
CXCL12
CXCR4
DCAF1
DDB1
DDB2
DDIT3
DNM1
DOCK1
DRD1
DVL1
DVL2
DVL3
E2F1
EEF1A1
EEF1E1
EFNA1
EFNA3
EFNA4
EFNA5
EFNB1
EFNB2
EGF
EGFR
EGR1
EIF2AK2
EIF2B1
EIF2S1
EIF2S2
EIF4A1
EIF4E
EIF4EBP1
EIF4G1
ELK1
ELMO1
EP300
EP400
EPHA2
EPHA3

EPHA4
EPHA6
EPHA7
EPHA10
EPHB1
EPHB2
ERBB2
ERBB3
ERBB4
ERCC1
ERCC2
ERCC3
ERCC4
ERCC5
ETS1
EZR
FANCA
FANCC
FANCD2
FANCE
FANCF
FANCG
FBXW11
FER
FKBP1A
FN1
FOS
FRMD6
FYN
GAB1
GH1
GHR
GNB1
GNB2
GNB3
GNG2
GNG4
GNG13
GNGT1
GPNMB
GRIN1
GRIN2B
GRK2
GRM1
GSK3B
H3-3A/H3-3B
HDAC1
HDAC2

HDAC3
HGF
HGFAC
HIF1A
HMGB1
HMGN1
HRAS
IGF1
IGF1R
IGFBP4
ILK
IRS1
ITGA2
ITGA2B
ITGB1
ITGB2
ITGB3
ITPR1
ITSN1
JUN
JUNB
KEAP1
KIT
KLF6
KRT19
LATS1
LCK
LEF1
LIMK1
LYN
MAFG
MAFK
MAML1
MAP2K4
MAP2K6
MAP3K1
MAP3K5
MAPK1
MAPK8
MAPK14
MAPKAPK2
MAX
MCL1
MDM2
MET
MKNK1
MLH1
MRE11

MSH2
MTOR
MYC
MYH9
MYL6
NBN
NCF2
NF2
NFATC1
NFATC2
NFE2L2
NFKB1
NFYC
NGEF
NOTCH1
NR2C2
NRG1
NRG3
PAK1
PAK3
PARP1
PCBD1
PDPK1
PGF
PHLPP1
PIK3CA
PIK3CG
PIK3R1
PIP5K1A
PLAUR
PLCB2
POR
PPP1CA
PPP2CA
PPP3CA
PPP3R1
PRKCA
PRKCB
PRKCD
PRKCE
PRKCG
PRKCH
PRKCI
PRKCQ
PRKCZ
PRKD1
PSEN2
PTEN

PTK2
PTPN11
PTTG1
PXN
RAC1
RAD50
RAD51
RAD9A
RASA1
RB1
RELA
RHEB
RHOA
RNF41
ROCK1
ROCK2
RPA1
RPL5
RPL6
RPL9
RPL11
RPL13
RPL15
RPL22
RPL23
RPL26
RPL38
RPS6
RPS7
RPS14
RPS23
RPS24
RPS6KA5
RPSA
SDC1
SDC2
SDCBP
SHC1
SIAH1
SIRT1
SKIL
SMAD1
SMAD2
SMAD4
SMARCA4
SMARCA5
SMARCE1
SNAI1

SOCS3
SP1
SPINT1
SRC
SREBF1
STAT1
STAT3
TAMALIN
TBL1X
TCF7L2
TIAM1
TLE1
TLE5
TP53
TP63
TREX1
TSC1
TSC2
TXN
USP7
VAV2
VCP
VEGFA
WASF2
XIAP
XPA
XPC
YES1
YY1

Table S6: Immunohistochemistry of the hub proteins and cell types (by scRNAseq) at Human Protein Atlas and RNA expression. Note: quantities of immunostaining from none (0), <25%, 25-75% and >75% shown under each protein. **, *: q<0.05, 0.01.**

Protein	Antibody	Diagnosis	Patient information	URL	Quantity	Cell types	RNA (Log2FC)
**EGFR**	CAB000035	Normal/GIM	Male, age 72, id: 301	v23.proteinatlas.org/ENSG00000146648-EGFR/tissue/stomach#img	25-75%	Goblet cells and fibroblasts	0.009
		GA	Female, age 50,id: 664	v23.proteinatlas.org/ENSG00000146648-EGFR/pathology/stomach+cancer#img	>75%		0.032
**SRC**	HPA030875	Normal/GIM	Female, age 81,id: 526	v23.proteinatlas.org/ENSG00000197122-SRC/tissue/stomach#img	25-75%	Goblet cells	0.027
		GA	Male, age 59, id: 2378	v23.proteinatlas.org/ENSG00000197122-SRC/pathology/stomach+cancer#img	>75%		0.497***
**PXN**	CAB003841	Normal/GIM	Male, age 56, id: 338	v23.proteinatlas.org/ENSG00000089159-PXN/tissue/stomach#img	0	Goblet cells, fibroblasts, T cells, and macrophages	0.276***
		GA	Female, age 86, id: 549	v23.proteinatlas.org/ENSG00000089159-PXN/pathology/stomach+cancer#img	>75%		0.371**
**JUN**	HPA066898 HPA066898	Normal/GIM	Female, age 57, id: 1467	v23.proteinatlas.org/ENSG00000177606-JUN/tissue/stomach#img	25-75%	Goblet cells, fibroblasts, plasma cells and T cells	0.256***
		GA	Male, age 54,id: 319	v23.proteinatlas.org/ENSG00000177606-JUN/pathology/stomach+cancer#img	>75%		-0.693***

**BRCA1**	CAB001946	Normal/GIM	Female, age 56, id: 2130	v23.proteinatlas.org/ENSG00000012048-BRCA1/tissue/stomach#img	<25%	Plasma cells and B cells	-0.020
		GA	Male, age 71,id: 703	v23.proteinatlas.org/ENSG00000012048-BRCA1/pathology/stomach+cancer#img	>75%		-0.012
**p53**	CAB039238	Normal/GIM	Female, age 57 id: 1467	v23.proteinatlas.org/ENSG00000141510-TP53/tissue/stomach#img	25-75%	Goblet cells, T cells, macrophages, B cells, and plasma cells	0.011
		GA	Male, age 48, id: 3044	v23.proteinatlas.org/ENSG00000141510-TP53/pathology/stomach+cancer#img	>75%		-0.046
**MDM2**	CAB000086	Normal/GIM	Female, age 56, id: 3233	v23.proteinatlas.org/ENSG00000135679-MDM2/tissue/stomach#img	>75%	Plasma cells, T cells, macrophages, goblet cells, fibroblasts	0.060
		GA	Male, age 59,id: 2473	v23.proteinatlas.org/ENSG00000135679-MDM2/pathology/stomach+cancer#img	>75%		0.065
**CTNNB1**	CAB001950	Normal/	Female, age 81, id: 526	V23.proteinatlas.org/ENSG00000168036-CTNNB1/tissue/stomach#img	25-75%	T cells, macrophages, B cells, plasma cells, fibroblasts and goblet cells	0.1134
		GA	Male, age 63, id: 1207	V23.proteinatlas.org/ENSG00000168036-CTNNB1/pathology/stomach+cancer#img	>75%		0.3326**
**CD44**	HPA005785	Normal/GIM	Female, age 56,id: 2130	v23.proteinatlas.org/ENSG00000026508-CD44/tissue/stomach#img	0	T cells, macrophages, B cells,	0.839***

		GA	Male, age 76, id: 2066	v23.proteinatlas.org/ENSG00000026508-CD44/pathology/stomach+cancer#img	0	plasma cells, fibroblasts, goblet cells	1.483***
